# Data on synthesis of methylene bisphosphonates and screening of their inhibitory activity towards HIV reverse transcriptase

**DOI:** 10.1016/j.dib.2016.07.039

**Published:** 2016-07-26

**Authors:** D.V. Yanvarev, A.N. Korovina, N.N. Usanov, O.A. Khomich, J. Vepsäläinen, E. Puljula, M.K. Kukhanova, S.N. Kochetkov

**Affiliations:** a Engelhardt Institute of Molecular Biology, Russian Academy of Sciences, Vavilova st.−32, Moscow, Russia; b School of Pharmacy, Biocenter Kuopio, University of Eastern Finland, Kuopio, Finland

## Abstract

Inorganic pyrophosphate (PPi) mimetics designed on a basis of methylenediphosphonic acid backbone are promising inhibitors of two key HIV replication enzymes, IN [1] and RT [2]. Herein, we present chemical synthesis of eleven methylenebisphosphonates (BPs) with their NMR and HRMS analysis synthesized via five different ways. Also, we present data on inhibition of HIV RT catalyzed phosphorolysis and polymerization by synthesized BPs using two methods based on denaturing urea PAGE. Tests were also performed for thymidine analogue mutations reverse transcriptase (TAM RT), which was expressed and purified for that. Structure–activity relationships and inhibitory activity data of synthesized BPs are presented in “Methylene bisphosphonates as the inhibitors of HIV RT phosphorolytic activity” [2].

**Specifications Table**

**Table:** 

Subject area	Chemistry, Biochemistry
More specific subject area	Chemical synthesis, purification, NMR and HRMS data, protein expression and purification, PAGE analysis
Type of data	Table, image, figure
How data was acquired	• High-resolution electrospray mass spectra were obtained on Applied Biosystems/MDS Sciex QSTAR XL (USA); • NMR spectra were recorded on 400 MHz (^1^H), 182 MHz (^31^P), and 92 MHz (^13^C) AMX III −400 Bruker spectrometer (USA); • All oligonucleotides were synthesized on an automatic ABI 3400 DNA synthesizer (Applied Biosystems, USA); • Radioactive products were detected using Typhoon FLA 9500 biomolecular imager “GE Healthcare” (UK) • pH measurements were performed using FEP30 Mettler Toledo (Switzerland) pH-meter with LE409-electrode.
Data format	Filtered and analyzed
Experimental factors	Starting compounds were either purchased or synthesized using already published synthetic protocols. The plasmid encoding TAM RT was a kind gift from Professor S.F.J. Le Grice.
Experimental features	Compounds were synthesized and their structure was identified by ^1^H, ^31^P, ^13^C and ^19^F NMR and confirmed by high resolution mass spectrometry. Compounds synthesized either here or earlier were tested as inhibitors of HIV RT catalyzed reactions.
Data source location	Engelhardt Institute of Molecular Biology, 32 Vavilov St., Moscow, Russia
Data accessibility	The data is included in this article.

## Value of the data

• The article describes the synthesis and physicochemical characterization of eleven new methylenebisphosphonates for biochemical research.
• The data possessed (validated) suppression of HIV RT catalyzed reactions by new methylenebisphosphonates in vitro and can be used for further design of HIV replication inhibitors.
• The data on inhibition of RT-pyrophosphorolysis and DNA-polymerization allow to deepen understanding of how HIV RT interacts with small molecule competitive inhibitors.

## 1. Data

The data presented here describe synthesis and physicochemical characterization of methylenebisphosphonates (BPs) of the following five different types: substituted hydroxymethylene BPs, α-aminomethylene BPs, β-aminomethylene BPs, α-alcoxymethylene BPs, and bis-alkylated BPs. We also present protocols for HIV reverse transcriptase purification and screening of synthesized BPs as its inhibitors *in vitro* and PAGE analysis of RT-catalyzed reactions.

## 2 Experimental design, materials and methods

All reagents were purchased from Acros Organics or Aldrich and used without drying or purification. Column chromatography was performed on Kieselgel (40–63 μm, Merck, Germany). TLC was carried out on Kieselgel 60 F_254_ precoated plates (Merck, Germany).

The inhibitor concentrations were measured by UV absorption according to molar extinction coefficients and compared with ^1^H NMR using the known concentrations of D1-*tert*-butanol in D_2_O. All pH measurements were conducted on FEP-30 Mettler Toledo pH-meter (Switzerland).

High-resolution mass spectra (HRMS) were registered in a positive ion mode on a Bruker Daltonics micrOTOF-Q II instrument using electrospray ionization (ESI). Interface capillary voltage: 4500 V; mass range from m/z 50 to 3000; external calibration (Electrospray Calibrant Solution, Fluka); nebulizer pressure: 0.4 bar; flow rate: 3 µL/min; dry gas: nitrogen (4 L/min); interface temperature: 200 °C.

NMR spectra were recorded on 400 MHz (^1^H), 182 MHz (^31^P), and 92 MHz (^13^C) AMX III −400 Bruker spectrometer. Chemical shifts are reported in parts per million (ppm) using tetramethylsilane (^1^H), tert-butanol-d_1_ (^13^C), and 85% H_3_PO_4_ (^31^P) as external standards. ^13^C and ^31^P NMR spectra were proton-decoupled unless otherwise specified.

Radioactive triphosphate [γ-^32^P]-ATP (molar activity 6000 Ci/mM) was a kind gift from Dr. Yu. S. Skoblov. HIV-1 RT_wt_ (21,500 U/ml) was purchased from “CalBioChem” (USA). Thermostable inorganic pyrophosphatase and T4 polynucleotide kinase were purchased from “New England BioLabs” (USA). All oligonucleotides were synthesized by the phosphoramidite method on an automatic ABI 3400 DNA synthesizer (Applied Biosystems, USA) under conditions recommended by manufacturer and purified by electrophoresis in a 20% polyacrylamide/7 M urea gel.

### 2.1 Chemistry

**Fig. 1:**
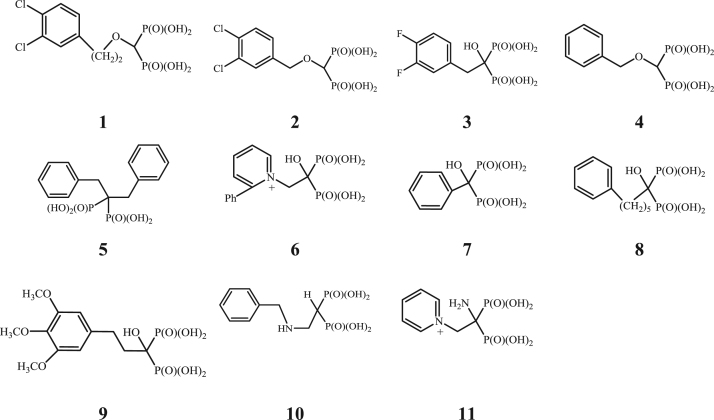
The bisphosphonates synthesized for this publication.

**Fig. 2:**
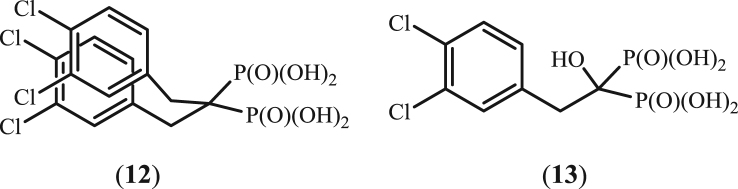
Bisphosphonates synthesized previously and studied as HIV RT inhibitors.

Bisphosphonates were prepared according to the following methods: (**3**), (**7**–**9**) (method A), (**1**), (**2**), (**4**) (method B), (**10**) by the method C, (**5**) D, (**6**) E and (**11**) F. For ^1^H, ^13^C, ^31^P, ^14^N, ^15^N NMR spectra images see Supplementry document Fig. 1, Fig. 2.

The bisphosphonates synthesized by **Method A** (**3**,**7**–**9**).

.

The acyl chlorides were prepared by 4–6 h refluxing of 5–7 g of the corresponding acid with 2 equivalents of thyonylchloride and 50 µL dimethylformamide in 8–15 mL of dry dichloromethane. After all volatile solvents were removed by vacuum evaporation then vacuum distillation at 0.8–1.5 mm Hg was applied (0.1 mm Hg in case of **9**) to give 75–80% of the target compounds as we described earlier [1].

After distillation acyl chlorides (3 mmol) were solved under Ar in dry benzene (10 mL) and was added dropwise on ice bath to a solution of 3 mmol triethylphosphite in dry benzene (10 mL) under vigorous stirring. Stirring was continued for additional 2–3 h at 5 °C then mixture of 3 mmol of diethylphosphite and 0.3 mmol diisopropylamine was added and stirring was continued for 4–5 h at 5 °C. All volatile component of reaction mixture were removed by vacuum evaporation and tetraesters **3**, **8**, **9** were purified by silica gel column chromatography with CHCl_3_–MeOH (0–15% MeOH) as an eluent. Solvents were removed in vacuo the residue was solved in dry chloroform and ethyl groups were routinely removed using 5 equivalents of TMSBr overnight at room temperature followed by methanolysis for 2 h.

#### 2.1.1 1-Hydroxy-2-(3,4-difluorophenyl)-ethylidene-1,1-bisphosphonate (3)

After methanolysis solvents were removed *in vacuo*, the residue was dissolved in 3 ml of water then 1 М NaOH was added until pH 7 was reached. Solution was lyophilized to give 850 mg of (**3**)•(2Na^+^) as white powder (yield 78% according to acyl chloride).

^1^H NMR (400 MHz; D_2_O, pH 1): *δ*=7.31 (m, *J*=12.2 Hz, *J*=8.1 Hz, *J*=1.9 Hz), 7.12 (m), 3.17 (t, *J*=12.6 Hz).

^13^C{^1^H} NMR (101 MHz; D_2_O, pH 1): *δ*=152.86 (dd, *J*_1_=52.0 Hz, *J*_2_=12.6), 150.93 (dd, *J*_1_=52.0 Hz, *J*_2_=12.6), 138.17 (broad s), 130.42 (d, *J*=3.0 Hz), 122.76 (d, *J*=16.6 Hz), 119.02 (d, *J*=16.6 Hz), 77.0 (t, *J*=128.2 Hz), 41.3 (s).

^31^Р{^1^H} NMR (162 MHz; D_2_O, pH 1): *δ*=18.65 (s).

^19^F NMR (471 MHz, D_2_O, pH 1) *δ* −139.01 (d, *J*=21.7 Hz, 1F), −140.90 (d, *J*=21.6 Hz, 1F).

HREIMS: calculated for C_8_H_10_F_2_O_7_P_2_ [М+H]^+^ 317.9870; found: 317.9867.

#### 2.1.2 1-Hydroxy-1-phenylmethylidene-1,1-bisphosphonate (7)

After methanolysis solvents were removed *in vacuo*, the residue was dissolved in 5 ml of water then 1 М KOH was added until pH 5 was reached. Clear solution was lyophilized to give 938 mg of (**7**)•(2 K^+^) as white powder (yield 91% according to acyl chloride).

^1^H NMR (400 MHz; D_2_O, pH 5): *δ*=7.69 (d, *J*_*HH*_=7.3 Hz, 2 H, Ph), 7.40 (dd, *J*_*HH*_=7.3 Hz, 2H, Ph), 7.36 (d, *J*_*HH*_=7.3 Hz, 1H, *p*-Ph).

^13^C{^1^H} NMR (101 MHz; D_2_O, pH 5): *δ*=140.09 (s), 131.41 (s), 131.07 (s), 129.42 (s), 78.86 (t, *J*^1^_*PC*_ = 145.2 Hz, P**C**P).

^31^Р{^1^H} NMR (162 MHz; D_2_O, pH 5): *δ*=16.02 (s).

HREIMS: calculated for C_7_H_10_O_7_P_2_ [М+H]^+^ 267.9902; found: 267.9897.

#### 2.1.3 1-Hydroxy-5-phenylhexylidene-1,1-bisphosphonate (8)

After methanolysis solvents were removed *in vacuo*, the residue was suspended in 6 ml of water then 1М KOH was added until pH 9 was reached. Clear solution was lyophilized to give 760 mg of (**8**)•(3 K^+^) as white powder (yield 61% according to acyl chloride).

^1^H NMR (400 MHz; D_2_O, pH 9): *δ*=d 7.35-7.20 (m, Ph, 5H), 2.63 (t, *J*=7.1 Hz, CH_2_–PCP, 2H), 2.08–1.96 (m, CH2–PCP, 2H), 1.66–1.57 (m, –(CH_2_)_3_–, 6H).

^31^Р{^1^H} NMR (162 MHz; D_2_O, pH 9): *δ*=18.5 (s).

^13^C{^1^H} NMR (101 MHz; D_2_O, pH 9, *t*-BuOD 30.3 ppm): *δ*=144.4 (s), 129.6 (s), 126.8 (s), 74.1 t (*J*_*PC*_=147 Hz), 35.2 (s), 33.9 (s), 32.0 (s), 30.1 (s), 29.9 (s).

HREIMS: calculated for C_12_H_20_O_7_P_2_ [М+H]+ 338.0684; found: 338.0680.

#### 2.1.4 1-Hydroxy-3-(3,4,5-trimethoxyphenyl)-propylidene-1,1-bisphosphonate (9)

After methanolysis solvents were removed *in vacuo*, the residue was dissolved in 5 ml of water then 1 М NaOH was added until pH 6 was reached. Solution was lyophilized to give 825 mg of (**9**)•(2Na^+^) as white powder (yield 64% according to acyl chloride).

^1^H NMR (400 MHz; D_2_O, pH 6): *δ*=6.72 (s, *m*-Ph, 2H), 3.83 (s, *m*-OCH_3_, 6H), 3.71 (s, *p*-OCH_3_, 3H), 2.88–2.82 (m, 2H, **C**H_2_–Ph), 2.25–2.10 (m, 2H, **C**H_2_–PCP).

^31^Р{^1^H} NMR (162 MHz; D_2_O, pH 6): *δ*=18.83 (s).

^13^C{^1^H} NMR (101 MHz; D_2_O, pH 6, *t*-BuOD 30.3 ppm): *δ*=155.07 (s, *m*-Ph), 142.97 (s, *p*-Ph), 137.44 (s, *ipso*-Ph), 108.79 (s, *o*-Ph), 76.74 (t, *J*_PC_=134.0 Hz, P-C-P), 63.61 (s, *p*-O**C**H_3_), 58.84 (s, *m*-O**C**H_3_).

HREIMS: calculated for C_12_H_20_O_10_P_2_ [М+H]^+^ 386.0532; found: 386.0535.

The bisphosphonates synthesized by **Method B** (**1**, **2**, **4**).

.

#### 2.1.5 Tetraethyl diazomethylenediphosphonate

The suspension of *t*-BuOK (2.68 g, 24 mmol) in toluene (15 mL) was cooled using an ice bath (0–5 °С). A solution of tetraethyl methylenediphosphonate (5.76 g, 20 mmol) in toluene (15 mL) was added dropwise to the reaction flask. Resulting viscous mixture was stirred for 20 min, where upon a solution of *p*-toluenesulfonyl azide (4.33 g, 22 mmol) in toluene (20 mL) was added dropwise while the temperature was kept below 5 °С. The mixture turned intensely yellow and a pale yellow solid began to precipitate. The ice bath was removed and stirring was continued for 3 h at 25 °С. The solid was separated by centrifugation; solvent was evaporated under reduced pressure. The residue was chromatographed on a silica gel column (benzene-ethyl acetate, 2:1) to afford 1.9 g (30%) of the desired product as yellow oil.

^1^H NMR (400 MHz, CDCl_3_): *δ*=1.34 (t, ^3^*J*_*Н–Н*_=7.0 Hz, 12Н, С**Н**_**3**_СН_2_О), 4.16 (m, 8Н, СН_3_С**Н**_**2**_О).

^13^C{^1^H} NMR (101 MHz; CDCl_3_): *δ*=16.2 (t, ^3^*J*_*С–Р*_ = 3.4 Hz, **С**Н_3_СН_2_О), 38.8 (t, ^1^*J*_*С–Р*_ = 204.1 Hz, Р–**С**N_2_–Р), 63.4 (t, ^2^*J*_*С–Р*_ = 2.7 Hz, СН_3_**С**Н_2_О).

^31^Р{^1^H} NMR (162 MHz; CDCl_3_): *δ*=11.9 (s).

^15^N{^1^H} inverse gated NMR (40.6 MHz; CDCl_3_): *δ*=−127.6 (t, ^3^*J*_*N–Р*_=3.6 Hz, С=N^+^=**N**^**-**^), −28.0 (s, С=**N**^**+**^=N^−^).

HREIMS: calculated for С_9_Н_20_N_2_О_6_P_2_ [М+H]^+^ 315.0869; found: 315.0873.

#### 2.1.6 General procedure for tetraethyl alkoxymethylenediphosphonate

To a solution of tetraethyl diazomethylenediphosphonate (0.5 g, 1.6 mmol) and alcohol (4.8 mmol) in 15 mL of anhydrous toluene Cu(OTf)_2_ (5.8 mg, 0.016 mmol) was added. The mixture was heated under reflux for 16 h. Solvent was evaporated under reduced pressure; the residue was chromatographed on a silica gel column, eluted with a CH_2_Cl_2_–MeOH (0–3% MeOH) to afford the desired product.

##### 2.1.6.1 *Tetraethyl benzyloxymethylenediphosphonate*

^1^H NMR (400 MHz; CDCl_3_): *δ*=1.22 (td, ^3^*J*_*Н–Н*_=7.1 Hz, ^4^*J*_*Н–Р*_=4.7 Hz, 12Н, С**Н**_**3**_СН_2_О), 3.96 (t, ^2^*J*_*Н–Р*_=17.2 Hz, 1Н, Р–С**Н**(OBn)–Р), 4.11 (m, 8Н, СН_3_С**Н**_**2**_О), 4.73 (s, 1Н, OC**H**_**2**_Ph), 7.23 (m, 5Н, **Ph**). ^13^C{^1^H} NMR (101 MHz; CDCl_3_): *δ*=16.2 (d, ^**3**^*J*_*С–Р*_=2.2 Hz, **С**Н_3_СН_2_О), 63.1 (m, СН_3_**С**Н_2_О), 71.6 (t, ^1^*J*_*С–Р*_=156.5 Hz, Р–**С**Н(OBn)–Р), 75.5 (t, ^3^*J*_*С–Р*_=5.0 Hz, O**C**H_2_Ph), 136.3 (**Ph**). ^31^Р{^1^H} NMR (162 MHz; CDCl_3_): *δ*=16.1 (s).

HREIMS: calculated for С_16_Н_28_О_7_P_2_ [М+Na]^+^ 417.1202; found: 417.1191.

##### 2.1.6.2 *Tetraethyl 3,4-dichlorobenzyloxymethylenediphosphonate*

^1^H NMR (400 MHz; CDCl_3_): *δ*=1.33 (td, ^3^*J*_*Н–Н*_=7.1 Hz, ^4^*J*_*Н–Р*_=2.8 Hz, 12Н, С**Н**_**3**_СН_2_О), 4.00 (t, ^2^*J*_*Н–Р*_=17.2 Hz, 1Н, Р–С**Н**(OAr)–Р), 4.21 (m, 8Н, СН_3_С**Н**_**2**_О), 4.78 (s, 1Н, OC**H**_**2**_Ar), 7.21 (dd, ^3^*J*_*Н–Н*_=8.2 Hz, ^4^*J*_*Н–Н*_=1.9 Hz, 1H, *o*-Ar), 7.39 (d, ^3^*J*_*Н–Н*_=8.2 Hz, 1H, *m*-Ar), 7.49 (d, ^4^*J*_*Н–Н*_=1.9 Hz, 1H, *o*-Ar). ^13^C{^1^H} NMR (101 MHz; CDCl_3_): *δ*=16.6 (**С**Н_3_СН_2_О), 63.5 (СН_3_**С**Н_2_О), 72.5 (t, ^1^*J*_*С–Р*_=157.0 Hz, Р–**С**Н(OAr)–Р), 74.5 (t, ^3^*J*_*С–Р*_=5.2 Hz, O**C**H_2_Ar), 127.7, 130.4, 130.5, 132.3, 132.7, 137.1. ^31^Р{^1^H} NMR (162 MHz; CDCl_3_): *δ*=14.3 (s).

HREIMS: calculated for C_16_H_26_O_7_P_2_Cl_2_ [М+Н]^+^=463.0609; found 463.0613.

##### 2.1.6.3 *Tetraethyl 3,4-dichlorophenethoxymethylenediphosphonate*

^1^H NMR (400 MHz; CDCl_3_): *δ*=1.29 (dt, ^4^*J*_*Н–Р*_=8.6 Hz, ^3^*J*_*Н–Н*_=7.1 Hz, 12Н, С**Н**_**3**_СН_2_О), 2.86 (t, ^3^*J*_*Н–Н*_=6.4 Hz, 2H, ArC**H**_**2**_CH_2_), 3.88 (t, ^2^*J*_*Н–Р*_=17.5 Hz, 1Н, Р–С**Н**(OR)–Р), 3.96 (t, ^3^*J*_*Н–Н*_=6.4 Hz, 2H, ArCH_2_C**H**_**2**_), 4.12 (m, 8Н, СН_3_С**Н**_**2**_О), 7.07 (dd, ^3^*J*_*Н–Н*_=8.2 Hz, ^4^*J*_*Н–Н*_=2.1 Hz, 1H, *o*-Ar), 7.31 (d, ^3^*J*_*Н–Н*_=8.2 Hz, 1H, *m*-Ar), 7.35 (d, ^4^*J*_*Н–Н*_=2.1 Hz, 1H, *o*-Ar). ^13^C{^1^H} NMR (101 MHz; CDCl_3_): *δ*=16.5 (**С**Н_3_СН_2_О), 35.5 (Ar**C**H_2_CH_2_), 63.4 (СН_3_**С**Н_2_О), 73.7 (t, ^1^*J*_*С–Р*_=157.2 Hz, Р–**С**Н(OR)–Р), 74.6 (t, ^3^*J*_*С–Р*_=4.7 Hz, ArCH_2_**C**H_2_), 128.6, 130.2, 130.4, 131.1, 132.2, 139.0. ^31^Р{^1^H} NMR (162 MHz; CDCl_3_): *δ*=14.1 (s).

HREIMS: calculated for C_17_H_28_O_7_P_2_Cl_2_ [М+Н]^+^=477.0765; found 477.0771.

#### 2.1.7 General procedure for alkoxymethylenediphosphonic acids

The mixture of tetraethyl alkoxymethylenediphosphonate (20 mg) and TMSCl (0.2 mL) was placed in a sealed tube and heated at 120 °С for 8 h. TMSCl was evaporated under reduced pressure; the residue was quenched with aq. MeOH. Solvent was evaporated under reduced pressure; product was obtained with quantitative yield.

##### 2.1.7.1 3,4-dichlorobenzyloxymethylenediphosphonic acid (1)

^1^H NMR (400 MHz; D_2_O): *δ*=3.72 (t, ^2^*J*_*Н–Р*_=15.8 Hz, 1Н, Р–С**Н**(OAr)–Р), 4.71 (s, 1Н, OC**H**_**2**_Ar), 7.32 (dd, ^3^*J*_*Н–Н*_=8.3 Hz, ^4^*J*_*Н–Н*_=1.9 Hz, 1 H, *o*-Ar), 7.45 (d, ^3^*J*_*Н–Н*_=8.3 Hz, 1 H, *m*-Ar), 7.60 (d, ^4^*J*_*Н–Н*_=1.9 Hz, 1 H, *o*-Ar).

^13^C{^1^H} NMR (101 MHz; D_2_O): *δ*=74.1 (t, ^3^*J*_*С–Р*_=4.8 Hz, O**C**H_2_Ar), 75.7 (t, ^1^*J*_*С–Р*_=138.7 Hz, Р–**С**Н(OAr)–Р), 128.5, 130.6, 130.7, 132.4, 132.8, 138.6.

^31^Р{^1^H} NMR (162 MHz; D_2_O): *δ*=11.9 (s).

HREIMS: calculated for C_8_H_10_Cl_2_O_7_P_2_ [М−H]^−^ 348.9218; found: 348.9219.

##### 2.1.7.2 3,4-dichlorophenethoxymethylenediphosphonic acid (2)

^1^H NMR (400 MHz; D_2_O): *δ*=3.00 (t, ^3^*J*_*Н–Н*_ = 7.6 Hz, 2H, ArC**H**_**2**_CH_2_), 3.67 (t, ^2^*J*_*Н–Р*_ = 15.0 Hz, 1Н, Р–С**Н**(OR)–Р), 3.98 (t, ^3^*J*_*Н–Н*_ = 7.6 Hz, 2H, ArCH_2_C**H**_**2**_), 7.33 (dd, ^3^*J*_*Н–Н*_ = 8.3 Hz, ^4^*J*_*Н–Н*_=1.8 Hz, 1H, *o*-Ar), 7.51 (d, ^3^*J*_*Н–Н*_ = 8.3 Hz, 1H, *m*-Ar), 7.58 (d, ^4^*J*_*Н–Н*_ = 1.8 Hz, 1H, *o*-Ar).

^13^C{^1^H} NMR (101 MHz; D_2_O): *δ*=35.1 (Ar**C**H_2_CH_2_), 73.9 (t, ^3^*J*_*С–Р*_=4.3 Hz, ArCH_2_**C**H_2_), 77.5 (t, ^1^*J*_*С–Р*_=130.6 Hz, Р–**С**Н(OR)–Р), 129.3, 129.6, 130.6, 131.2, 131.7, 139.9.

^31^Р{^1^H} NMR (162 MHz; D_2_O): *δ*=12.7 (s).

HREIMS: calculated for C_9_H_12_Cl_2_O_7_P_2_ [М-H]^-^ 362.9377; found: 362.9374.

##### 2.1.7.3 *Benzyloxymethylenediphosphonic acid (4)*

^1^H NMR (400 MHz; D_2_O): *δ*=3.88 (t, ^2^*J*_*Н–Р*_=15.6 Hz, 1Н, Р–С**Н**(OBn)–Р), 4.87 (s, 1Н, OC**H**_**2**_Ph), 7.55 (m, 5Н, **Ph**).

^13^C{^1^H} NMR (101 MHz; D_2_O): *δ*=75.4 (t, ^3^*J*_*С–Р*_=2.0 Hz, O**C**H_2_Ph), 76.0 (t, ^1^*J*_*С–Р*_=134.7 Hz, Р–**С**Н(OBn)–Р), 128.50 (s), 128.9 (s), 129.1 (s) 138.3 (s).

^31^Р{^1^H} NMR (162 MHz; D_2_O): *δ*=12.3 (s).

HREIMS: calculated for C_8_H_12_O_7_P_2_ [М−H]^−^ 282.0058; found: 295.0023.

The bisphosphonate synthesized by **Method C** (**10**).

.

#### 2.1.8 *Vinylidenediphosphonic acid (VDPA)*

VDPA was prepared by thermal dehydration of tetrasodium salt of etidronic acid followed by partial deionization of tetrasodium VDPA by CO_2_ gas [3]. The thermal conditions and dehydration time were optimized (350 °C, 5 h) to get 97–100% conversion of etidronate. Reaction was monitored by ^31^P NMR analysis, which revealed VDPA and PP_i_ to be the main reaction products (>90%).

Tetrasodium etidronate was heated 5 h at 350 °C in muffle furnace. After the reaction was completed and cooled to room temperature, the residue was dissolved in a minimal amount of water at 20 °C, and insoluble materials were filtered off. The filtrate was diluted twofold with water and a CO_2_ flow was passed through at +5 °C up to pH 6. The solution was left at this temperature for additional 5–6 h and the precipitated NaHCO_3_ was filtered off. The residue was crystallized from glacial acetic acid and final purity of disodium VDPA was >95%. Crystals were diluted with water and VDPA concentration measured by ^1^H-NMR. This solution was used for syntheses presented below.

##### 2.1.8.1 2-N-benzyl-2-aminoethylidene-1,1-bisphosphonate (10)

A solution of disodium VDPA (1 eq) in 5 ml of 95% acetic acid and benzylamine (2 eq) was stirred at 80 °C till homogenous syrup formation. Then reaction mixture was sealed in a glass tube and heated at 120 °C for 5 h. The reaction mixture was poured into 50% water/ethanol mixture, acidified with HCl to pH 1 and allowed to crystallize at 5 °C. The precipitated zwitterionic bisphosphonate was of >95% purity according to ^1^H, and ^31^P NMR data.

^1^H NMR (400 MHz; D_2_O, pH 8): *δ*=7.39–7.28 (m, 5 H, Ph), 4.32 (s, 2H, Ph–**CH**_**2**_), 3.39 (dt, *J*^3^*_PH_*=15.1 Hz, *J*^2^*_HH_*=6.5 Hz, 2H, PCP–**CH**_**2**_), 2.05 (tt, *J*^2^*_PH_*=20.0 Hz, *J*^2^*_HH_*=6.5 Hz, 1H, PC**H**P).

^13^C{^1^H} NMR (101 MHz; D_2_O, pH 8): *δ*=135.3 (s), 132.1 (s), 131.9 (s), 131.6 (s), 48.9 (s), 37.7 (t, *J*^1^*_PC_*=110.1 Hz).

^31^Р{^1^H} NMR (162 MHz; D_2_O, pH 8): *δ*=16.6 (s).

HREIMS: calculated for C_9_H_15_NO_6_P_2_ [М+H]^+^ 295.0374; found: 295.0374.

The bisphosphonate synthesized by **Method D** (5).

.

#### 2.1.9 ***1,1-dibenzyl-methylene-1,1-bisphosphonic acid (5)***

A solution of 1.2 mL (4 mmol) tetraisopropyl methylenebisphosphonate in dry THF (5 mL) stirred under the Ar atmosphere at 0 °C was added to 400 mg (10 mmol) of NaH (60% suspension in oil) in dry THF (5 mL). Stirring was continued for 1 h at, and then for 3 h at rt. A solution of 1.27 g (10 mmol) benzylchloride in THF (5 mL) was added to the resulted carbanion solution and stirred overnight at rt. The reaction was quenched with saturated NH_4_Cl (20 mL), extracted with DCM (3×50 mL), dried (Na_2_SO_4_) and concentrated *in vacuo*. The target bisphosphonate was purified as it was previously reported [3], by column chromatography on silica gel, eluting with EtOAc/MeOH gradient (0→25% MeOH). The tetraisopropyl ester was refluxed with 48% HBr (4 mL) for 2 h followed by co-evaporation with water (3×15 mL) and drying *in vacuo* in desiccator over P_2_O_5_ and KOH to give target (**5**) as viscous oil. Yield 0.96 g as free acid (67% according to tetraisopropyl methylenebisphosphonate).

^1^H NMR (400 MHz; D_2_O, pH 5): *δ*=7.5 (d, *J*=6.7 Hz, 6H), 7.3–7.2 (m, 4H), (t, *J*=16.3 Hz, 4H).

^13^C{^1^H} NMR (101 MHz; D_2_O, pH 5): *δ*=141.0 (s), 134.5 (s), 130.3 (s), 129.1 (s), 50.1 (t, *J*=113.1 Hz), 41.2 (t, *J*=3.4 Hz).

^31^Р{^1^H} NMR (162 MHz; D_2_O, pH 5): *δ*=22.5 (s).

HREIMS: calculated for C_15_H_18_O_6_P_2_ [М+H]^+^ 356.0578; found: 356.0573.

The bisphosphonate synthesized by **Method E** (6).

.

#### 2.1.10 ***1-Hydroxy-2-(2-phenylpyridine-1-yl)ethylidene-1,1-bisphosphonic acid (6)***

Ethylbromoacetate (6 mmol) was added to a solution of the substituted pyridine (5 mmol) in ether (15 mL) and the reaction mixture was stirred overnight at rt to give the substituted pyridinium bromides as white precipitates by the end of reaction. To improve the yield, supernatant was kept for 2 h at 0 °C and the formed crystals were added to ones formed during the course of reaction. The crystals were suspended in 4 ml of 2 M HCl and refluxed for 2 h, then solvent was removed *in vacuo* and the residue was re-evaporated with 5 ml of water. Yield was >95% and purity was >99% according to ^1^H-NMR spectrum.

The obtained *o*-substituted pyridiniumacetic acid (2 mmol) was dissolved in the mixture of phosphorous acid (2 mmol) and methanesulfonic acid (5 mL) in dry benzene (10 mL) and was refluxed for 10 min till the mixture became homogenous. The reaction mixture was cooled to 50 °C and a solution of POCl_3_ (3 mmol) in dry benzene (10 mL) was added dropwise for 30 min, maintaining the temperature below 70 °C, then the reaction mixture was stirred with reflux for addition 5 h. The viscous yellowish reaction mixture was cooled to ~40 °C and pooled into the ice-cooled 3N HCl (50 mL) under vigorous stirring and then refluxed for 2 h. The water layer was separated, dried *in vacuo*, and re-evaporated with water (2×8 mL), then dissolved in 3–4 mL of boiling water; pH changed to 4 by adding 2 M KOH and the solution was kept for ~24 h at +5 °C till the bisphosphonate (**6**) crystallized. Yield 68% as white crystals of dipotassium salt.

^1^H NMR (400 MHz, D_2_O. pH 2) *δ* 9.18 (d, *J*=5.6 Hz, 1H), 8.47 (t, *J*=7.7 Hz, 1H), 8.04–7.80 (m, 1H), 7.59 (s, 3H), 5.31 – 4.98 (m, 1H).

^31^Р{^1^H} NMR (162 MHz, D_2_O. pH 2) *δ* 8.83.

^13^C{^1^H} NMR (101 MHz; D_2_O, pH 2): *δ* 159.90, 148.91, 137.04, 135.12, 132.61, 131.90, 126.80, 76.01 ( t, *J*=134.0 Hz).

HREIMS: calculated for C_13_H_16_NO_7_P_2_ [М+H]^+^ 360.0402; found: 360.0405.

The bisphosphonate synthesized by **Method F** (**11**).

.

#### 2.1.11 ***1-Amino-2-pyridine-1-yl-ethylidene-1,1-bisphosphonic acid (11)***

Bromoacetonitrile (6 mmol) was added to a solution of the pyridine (5 mmol) in ether (15 mL) and the reaction mixture refluxed for 6 h to give the substituted pyridinium bromides as white precipitates after cooling to rt. The precipitates were filtered, washed with ether (2×10 mL) and dried *in vacuo*. Yield was >96%.

Herein, we optimized the synthesis of 1-amino-BPs published earlier [4] by changing ionic liquid and temperature conditions. A suspension of nitrile (2 mmol), phosphorous acid (2 mmol) and methanesulfonic acid (5 mL) in dry benzene (10 mL) was refluxed for 40 min till the mixture became homogenous. Reaction mixture was cooled to 60–65 °C and a solution of POCl_3_ (3 mmol) in dry benzene (10 mL) was added dropwise for 30 min, maintaining the temperature below 70 °C, then the reaction mixture was stirred with reflux for additional 12 h. The viscous yellowish reaction mixture was cooled to ~40 °C and pooled into the ice-cooled 3N HCl (50 mL) under vigorous stirring and then refluxed for 2 h. The water layer was separated, concentrated in vacuo to a volume of ~3 mL and kept for ~24 h at +5 °C till the bisphosphonate (11) crystallized. Yield 72% as white crystals.

^1^H NMR (500 MHz, D_2_O) *δ* 8.98 (d, *J*=6.1 Hz, 2H), 8.62 (t, *J*=7.9 Hz, 1H), 8.09 (t, *J*=7.2 Hz, 2H), 5.24 (t, *J*=9.8 Hz, 2H).

^31^Р{^1^H} NMR (202 MHz, D_2_O) *δ* 8.83.

^13^C{^1^H} NMR (126 MHz, D_2_O) *δ* 146.94, 145.99, 127.68, 61.45, 58.04, 57.07, 56.09.

HREIMS: calculated for C_7_H_13_N_2_O_6_P_2_ [М+H]^+^ 283.0243; found: 283.0240.

### 2.2 RT inhibition

#### 2.2.1 [5′-^32^P]-labeled primer–template complex preparation

Synthetic 42-nt oligonucleotide (5′-CCA GTT AGC GTA GTC AAG GCT CGA GAC TAC AGG AAT TGA CGG-3′) and 19-nt oligonucleotide (5′-CCG TCA ATT CCT GTA GTC T-3′) were used to obtain the primer–template complex. The reaction mixture (30 μl) contained 70 mM Tris–HCl (pH 7.6), 10 mM MgCl_2_, 5 mM dithiothreitol, 50 pmol of 19-nt primer, 5U T4 polynucleotide kinase, and 100 μCi of [γ-^32^P]rATP. After 1 h of incubation at 37 °C, T4 polynucleotide kinase was inactivated by heating at 65 °C for 20 min. To obtain the primer–template complex, [5′-^32^P]-primer was annealed with a 1.5-excess 42-nt DNA template by heating at 65 °C for 5 min and cooling to room temperature. This complex was purified using an illustra^™^ microspin G-25 column from “GE Healthcare” (UK).

#### 2.2.2 Expression and purification of ТАМ reverse transcriptase enzyme

The plasmid p6HRT encoding His-tagged TAM HIV-1 reverse transcriptase (RT; M41L, D67N, K70R, T215Y, K219Q) was a kind gift from Professor S.F.J. Le Grice. The expression cassette contained also HIV-1 protease gene allowing formation of p66/p51 heterodimer. The protein was expressed in Rosetta (DE3) *E. coli* strain. TAM RT was purified using standard Ni-NTA agarose procedure [5] (Lysis buffer: 25 mM Tris–HCl, 350 mM NaCl, 5 mM β-mercaptoethanol, 0,1% Triton X-100, 10% glycerol, 1 mM PMSF, and protease inhibitor cocktail), followed by two steps of the dialysis (D1: 25 mM Tris–HCl, 350 mM NaCl, 5 mM β-mercaptoethanol, 10% glycerol; D2: 25 mM Tris–HCl, 350 mM NaCl, 5 mM β-mercaptoethanol, 50% glycerol), yielding 2 μg of p66/p51 heterodimer per liter of cell culture. The enzyme activity was 5500 U/mL.

#### 2.2.3 Inhibition of PP- and ATP-mediated phosphorolysis

**Fig. 3:**
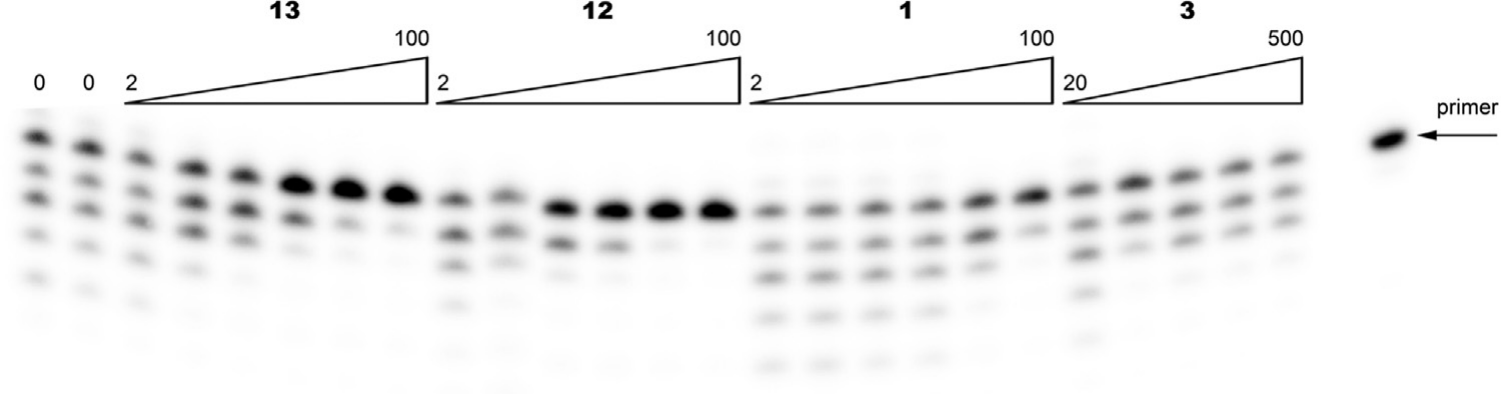
PAGE of RT catalyzed pyrophosphorolysis inhibited by 2, 5, 10, 20, 50, and 100 μM of BPs **1**, **12**, **13** and 20, 50, 100, 200, and 500 μM of BP **3**.

**Fig. 4:**
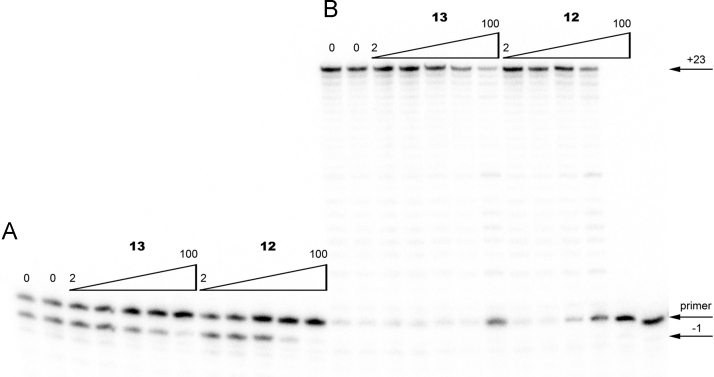
PAGE of RT catalyzed (A) pyrophosphorolysis inhibited by 2, 5, 20, 50, and 100 μM of BPs **12** and **13**; (B) elongation inhibited by 20, 50, 100, 200, and 500 μM of BPs **12** and **13**.

The [5′-^32^P]-primer–template complexes (10 nM) were incubated with 0.5U HIV RT and 300 μM PPi or 3 mM ATP in case of PP- and ATP-mediated phosphorolysis respectively in 10 μl of the reaction buffer (50 mM Tris–HCl pH 8.0 or pH 7.5 for PP- and ATP-mediated phosphorolysis, respectively, 10 mM MgCl_2_, 60 mM KCl) in the presence of increasing concentrations of an bisphosphonate inhibitors (Figs. 3 and 4A) at 37 °C for 30 min.

#### 2.2.4 Inhibition of RT polymerase activity

The [5′-^32^P]-primer–template complexes (10 nM) were incubated with 0.5U HIV RT and 1 μM of dATP, dTTP, dGTP, and dCTP in 10 μl of the reaction buffer (50 mM Tris–HCl pH 8.0, 10 mM MgCl_2_, 60 mM KCl) in the presence of increasing concentrations of an bisphosphonate inhibitors (Fig. 4B) at 37 °C for 30 min.

#### 2.2.5 Quantitative analysis of the RT inhibition

The reactions containing [5′-^32^P]-primer–template complexes were stopped by 5 μl of stop solution, containing formamide, 10% of EDTA and 1 mg/ml each of bromphenol blue and xylene cyanol. 5 μl aliquot of the reaction was loaded onto 15% denaturing urea gel and subjected to electrophoresis (2 h, 2400 V). Radioactive products were detected using Typhoon FLA 9500 biomolecular imager “GE Healthcare” (UK). Spots on radioautograph images were interpreted using Opti-Quant software (Packard Inc., USA) and IC_50_ were obtained graphically.

## Author contributions

DY and SK planned the project and designed the research, JW and EP performed the physical–chemical experiments, MK, AK and NU performed biochemical measurements and enzymes purification, DY and OK conducted chemical synthesis and purification, DY, SK and MK wrote the paper, and all co-authors commented on the paper.

## Appendix A Supplementary material

Supplementary material contain ^1^H, ^13^C, ^31^P, ^14^N, ^15^N NMR data for all new and intermediate BPs and IUPAC International Chemical Identifiers (InChI Keys)
