# Supplementary material for: Data on synthesis of methylene bisphosphonates and screening of their inhibitory activity towards HIV reverse transcriptase
Source: Data Brief. 2016 Jul 26;8:1157–67. doi: 10.1016/j.dib.2016.07.039 (PMC4978200; doi:10.1016/j.dib.2016.07.039)
Supplement: Supplementary file 2 — Supplementary material contain 1H, 13C, 31P, 14N, 15N NMR data for all new and intermediate BPs and IUPAC International Chemical Identifiers (InChI Keys) [file mmc2.pdf]

SUPPORTING MATERIALS
FOR DATA IN BRIEF 2016

Data on synthesis of methylene bisphosphonates and screening of their inhibitory activity towards HIV reverse transcriptase

D.V. Yanvarev[a,#,||], A.N. Korovina[a,||], N.N. Usanov[a], O.A. Khomich[a], J. Vepsäläinen[b], E. Puljula[b], M. K. Kukhanova[a], and S.N. Kochetkov[a]

[a] Engelhardt Institute of Molecular Biology, Russian Academy of Sciences, Vavilova st.-32, Moscow, Russia
[b] School of Pharmacy, Biocenter Kuopio, University of Eastern Finland, Kuopio, Finland
# Corresponding author, contact email: *JDmitry@hotmail.com*
|| These authors contributed equally to this work

BP №	**Structure**	**IUPAC International Chemical Identifier** (InChI Key)	Page number
1	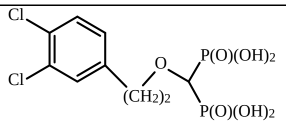	OEWVMFPQVDRUDR-UHFFFAOYSA-N	S3
2	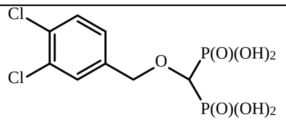	OBANHCGDRQOQPQ-UHFFFAOYSA-N	S9
3	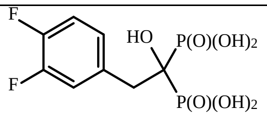	OQJRJVAZUXDROX-UHFFFAOYSA-N	S15
4	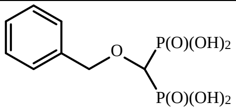	NDWDZLZLXYCQQA-UHFFFAOYSA-N	S19
5	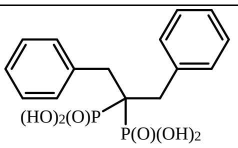	HOXDKQBBVAXHCK-UHFFFAOYSA-N	S25
6	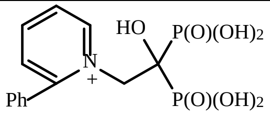	QPQPLHKVKCOFRT-UHFFFAOYSA-N	S31
9	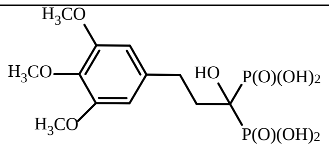	RSQJUCZOAHNZBI-UHFFFAOYSA-N	S35
11	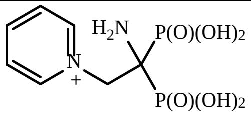	OEVAWJQRISOENR-UHFFFAOYSA-N	S38
	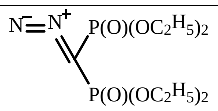	GWFURKNOQXNBFT-UHFFFAOYSA-N	S41-S46
	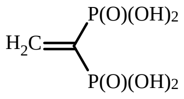	LUHPUPVJIVTJOE-UHFFFAOYSA-N	S47-S48

kr9975

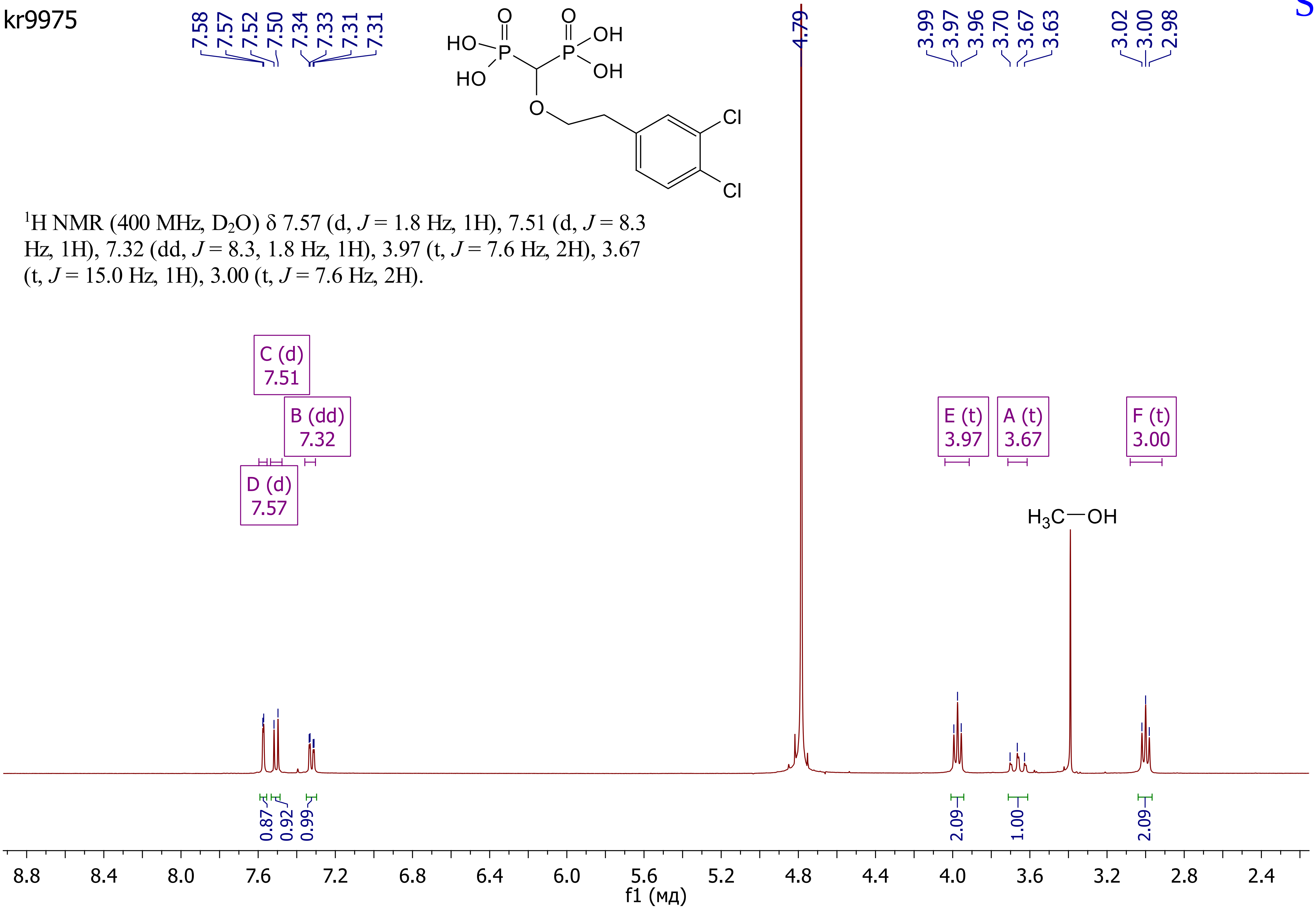

^{1}H NMR (400 MHz, D_2O) δ 7.57 (d, *J* = 1.8 Hz, 1H), 7.51 (d, *J* = 8.3 Hz, 1H), 7.32 (dd, *J* = 8.3, 1.8 Hz, 1H), 3.97 (t, *J* = 7.6 Hz, 2H), 3.67 (t, *J* = 15.0 Hz, 1H), 3.00 (t, *J* = 7.6 Hz, 2H).

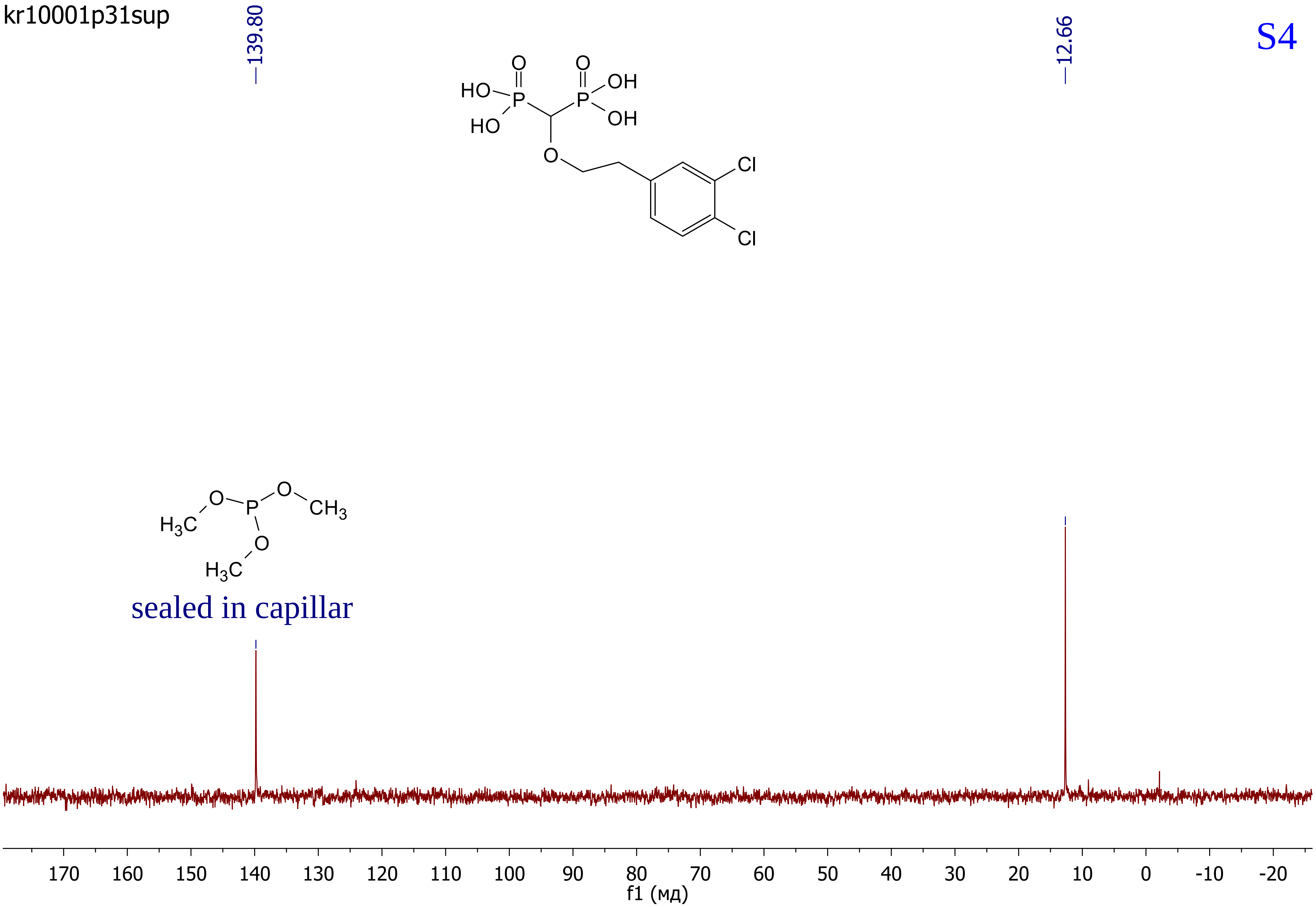
kr10001p31sup
139.80
12.66
HO
HO
OH
OH
O
O
O
P
P
Cl
Cl
H_3C
O
P
O
CH_3
O
H_3C
sealed in capillar
170 160 150 140 130 120 110 100 90 80 70 60 50 40 30 20 10 0 -10 -20
f1 (мд)

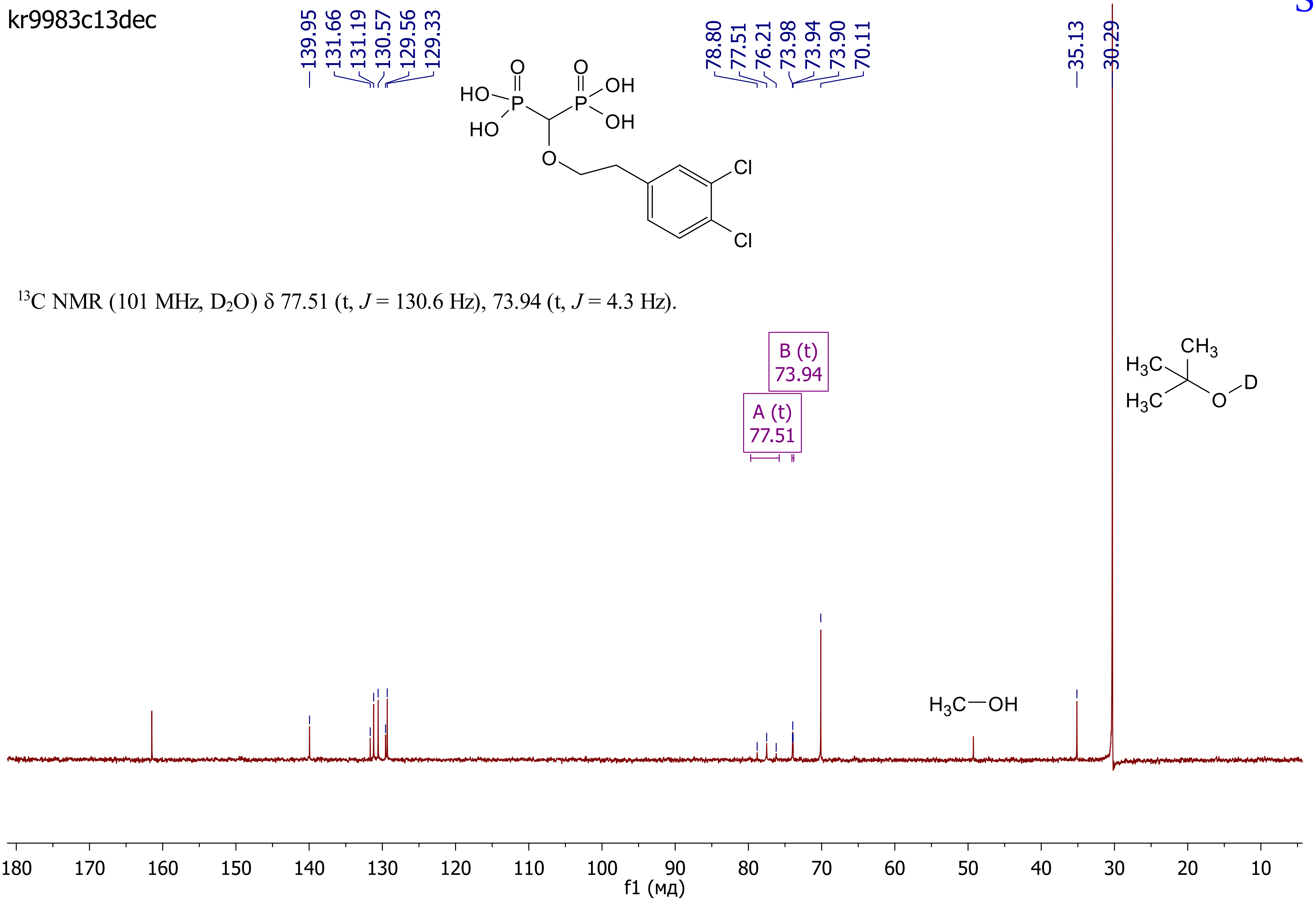

^{13}C NMR (101 MHz, D_2O) δ 77.51 (t, *J* = 130.6 Hz), 73.94 (t, *J* = 4.3 Hz).

kr9913

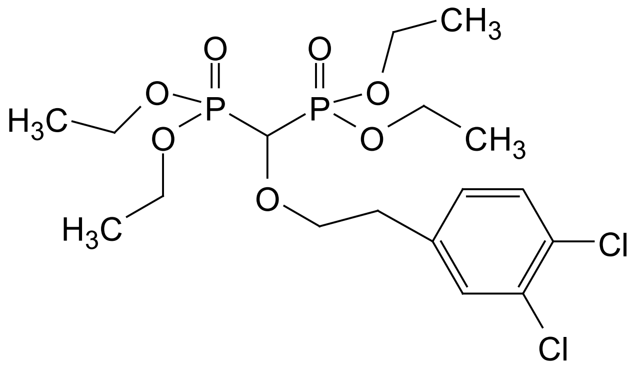

^{1}H NMR (400 MHz, CDCl$_3$) δ 7.35 (d, *J* = 2.0 Hz, 1H), 7.31 (d, *J* = 8.2 Hz, 1H), 7.07 (dd, *J* = 8.2, 2.1 Hz, 1H), 3.96 (t, *J* = 6.4 Hz, 1H), 3.88 (t, *J* = 17.5 Hz, 1H), 2.86 (t, *J* = 6.4 Hz, 1H), 1.29 (dt, *J* = 8.6, 7.1 Hz, 1H).

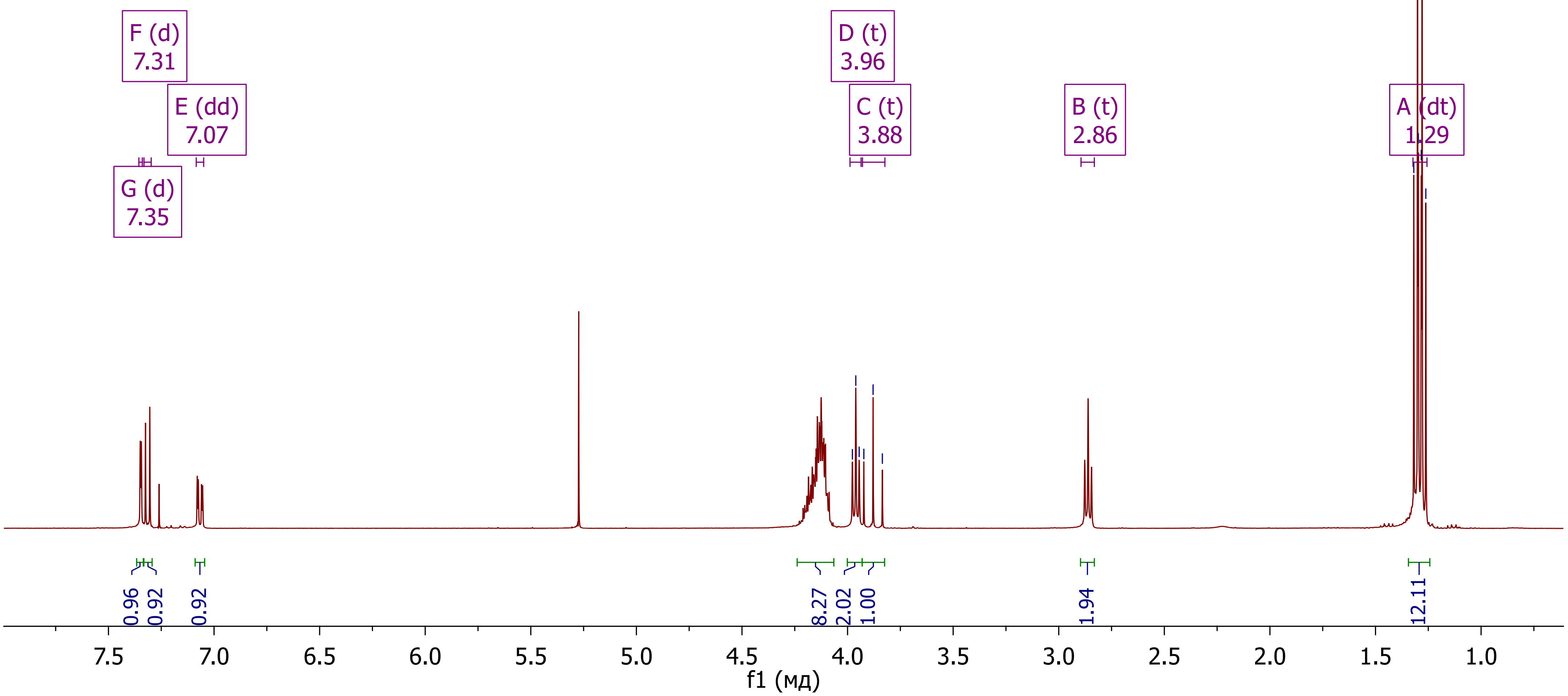

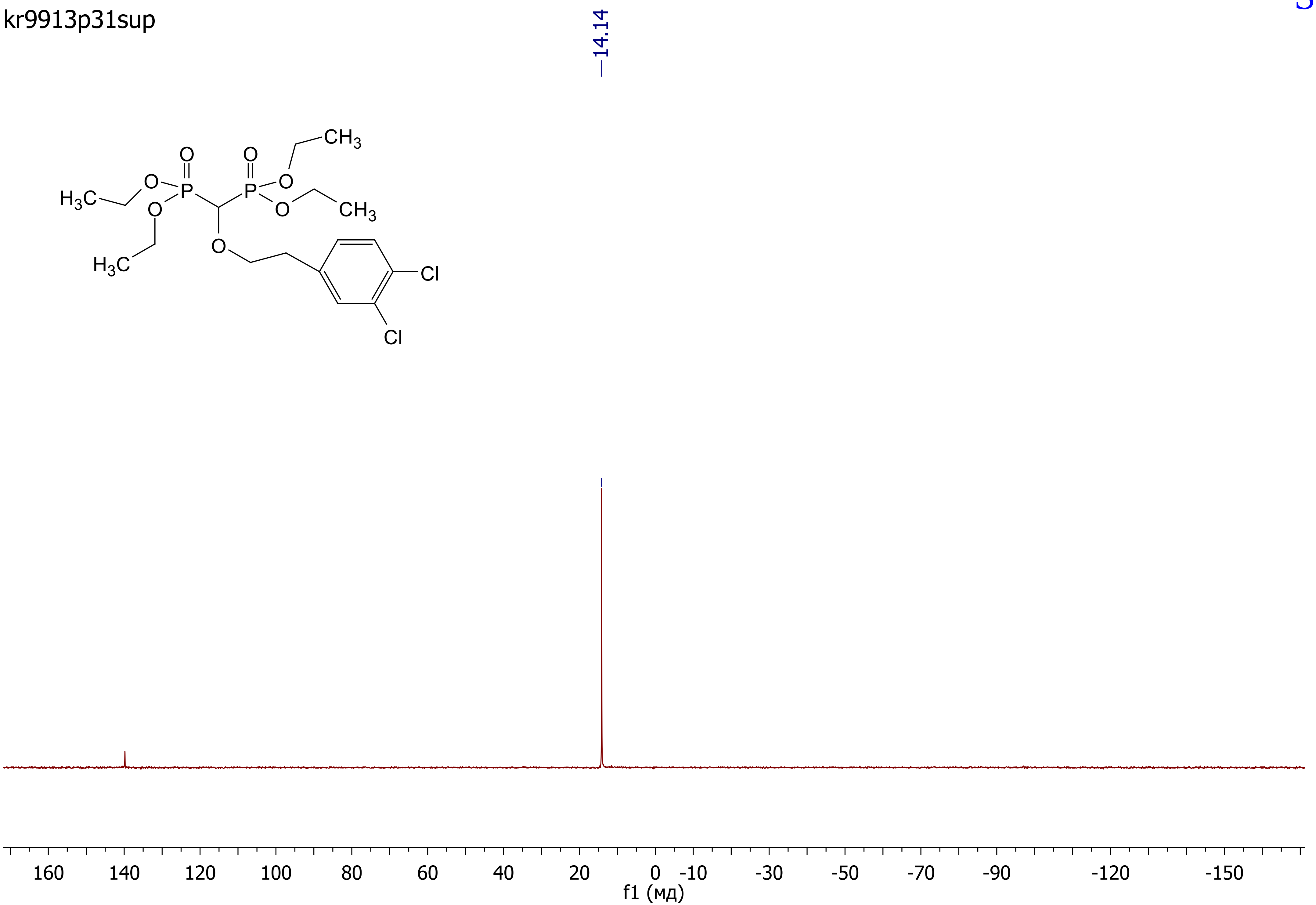
kr9913p31sup
14.14
H3C
O
P
O
O
P
O
O
CH3
CH3
H3C
O
Cl
Cl
160
140
120
100
80
60
40
20
0
-10
-30
-50
-70
-90
-120
-150
f1 (мд)

kr9913c13dec

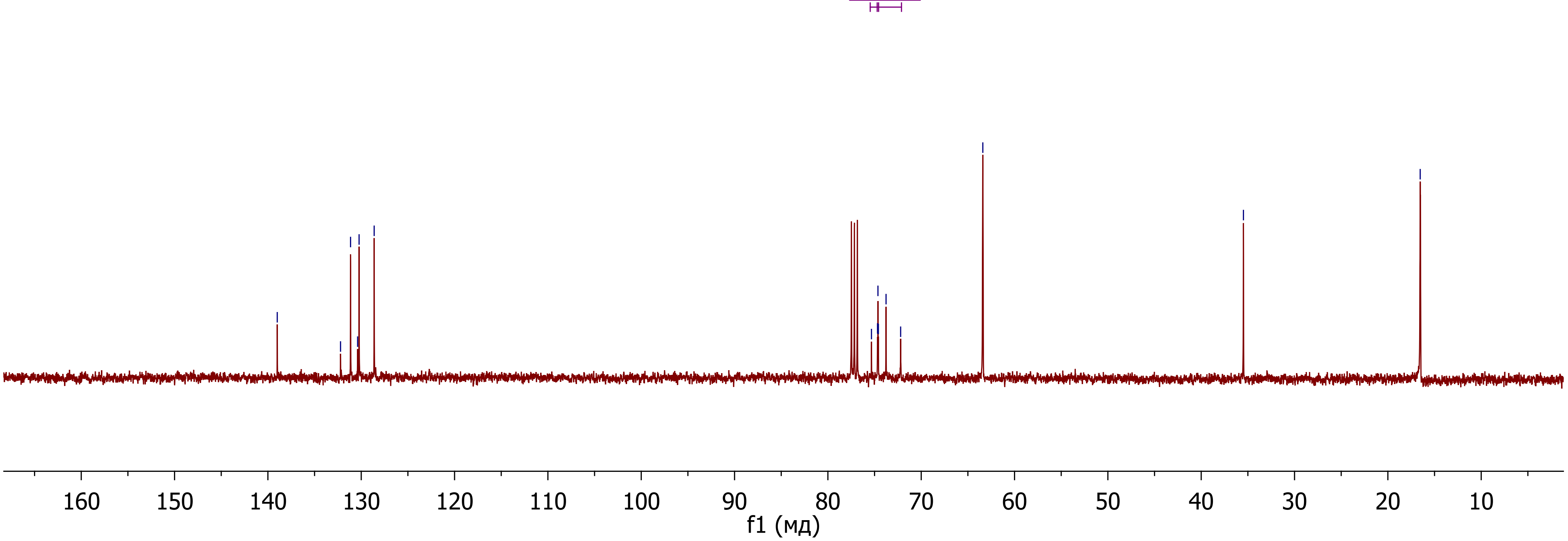

^{13}C NMR (101 MHz, $CDCl_3$) δ 74.64 (t, *J* = 4.7 Hz), 73.77 (t, *J* = 157.2 Hz).

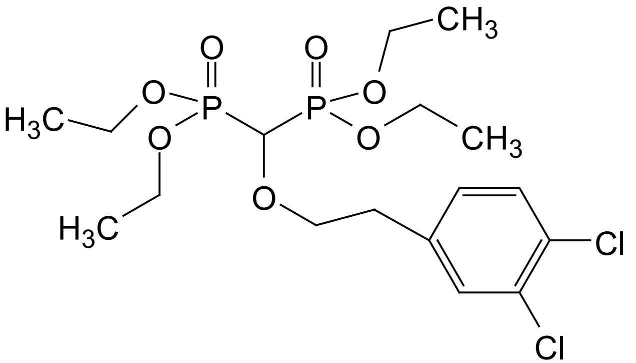

kr9990

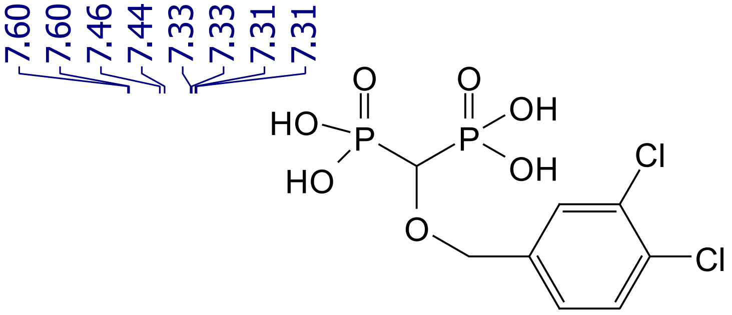

^{1}H NMR (400 MHz, D_2O) δ 7.60 (d, J = 1.8 Hz, 1H), 7.45 (d, J = 8.3 Hz, 1H), 7.32 (dd, J = 8.3, 1.9 Hz, 1H), 3.72 (t, J = 15.8 Hz, 1H).

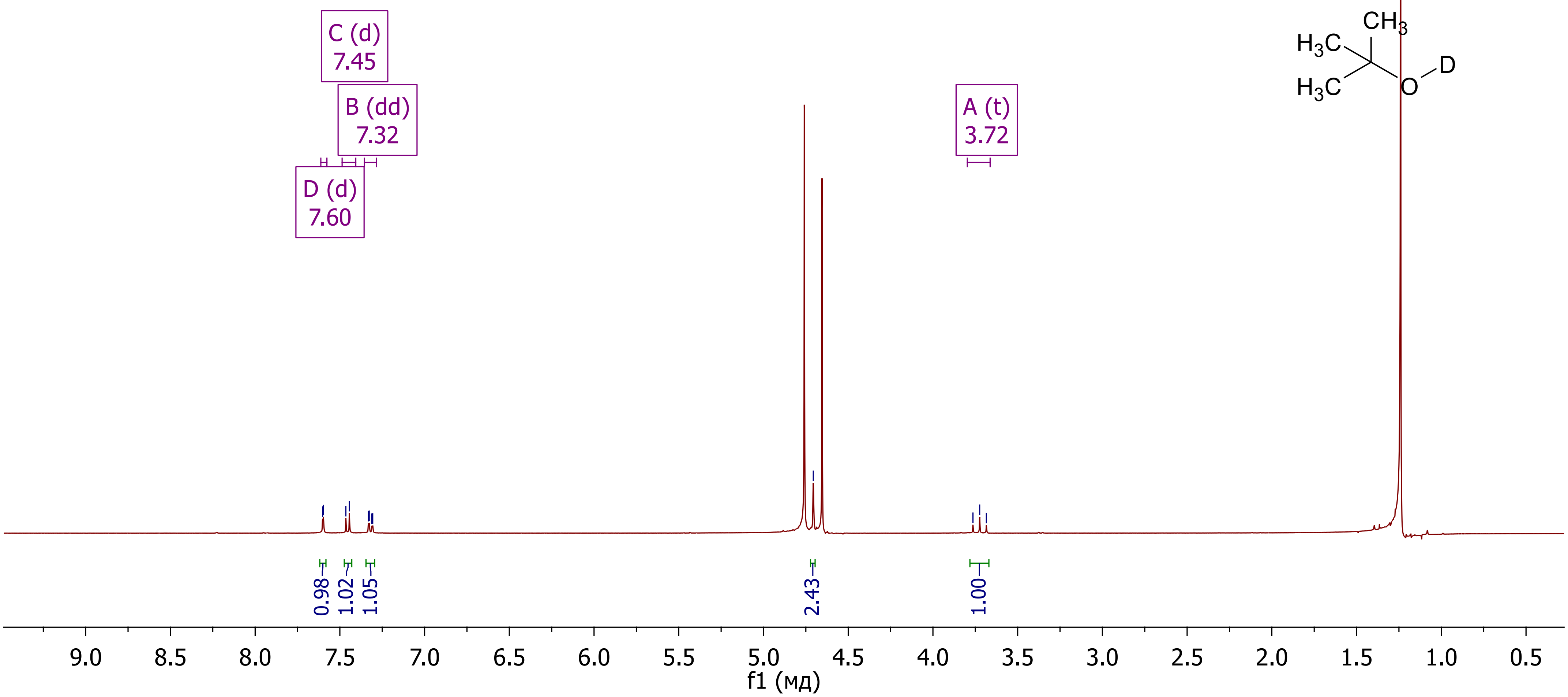

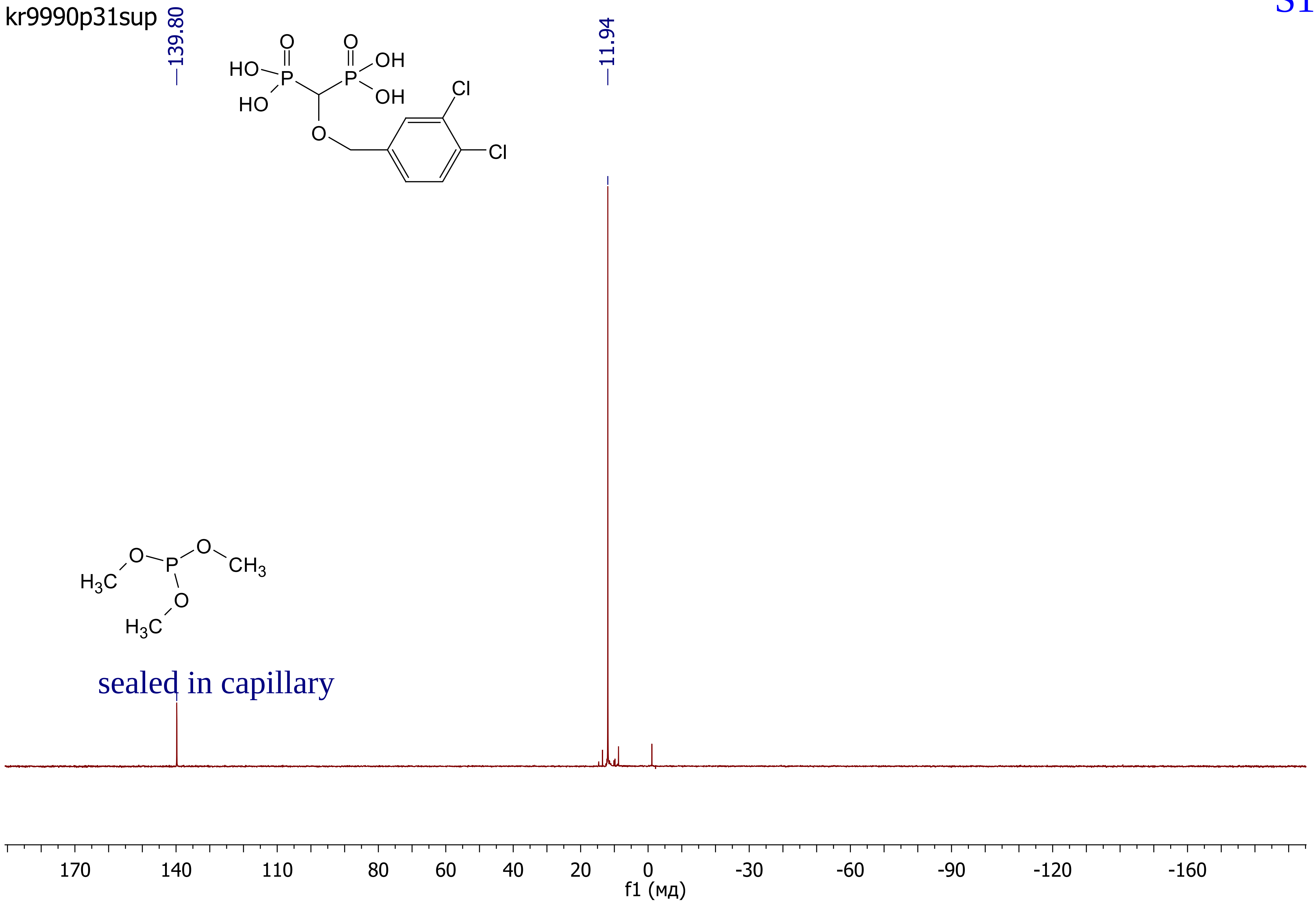
kr9990p31sup
139.80
11.94
sealed in capillary
170
140
110
80
60
40
20
0
-30
-60
-90
-120
-160
f1 (мд)

kr9990c13dec

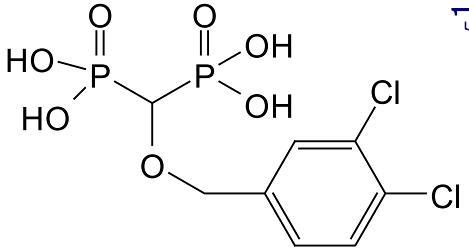

^{13}C NMR (101 MHz, D_2O) δ 75.70 (t, J = 138.7 Hz), 74.05 (t, J = 4.8 Hz).

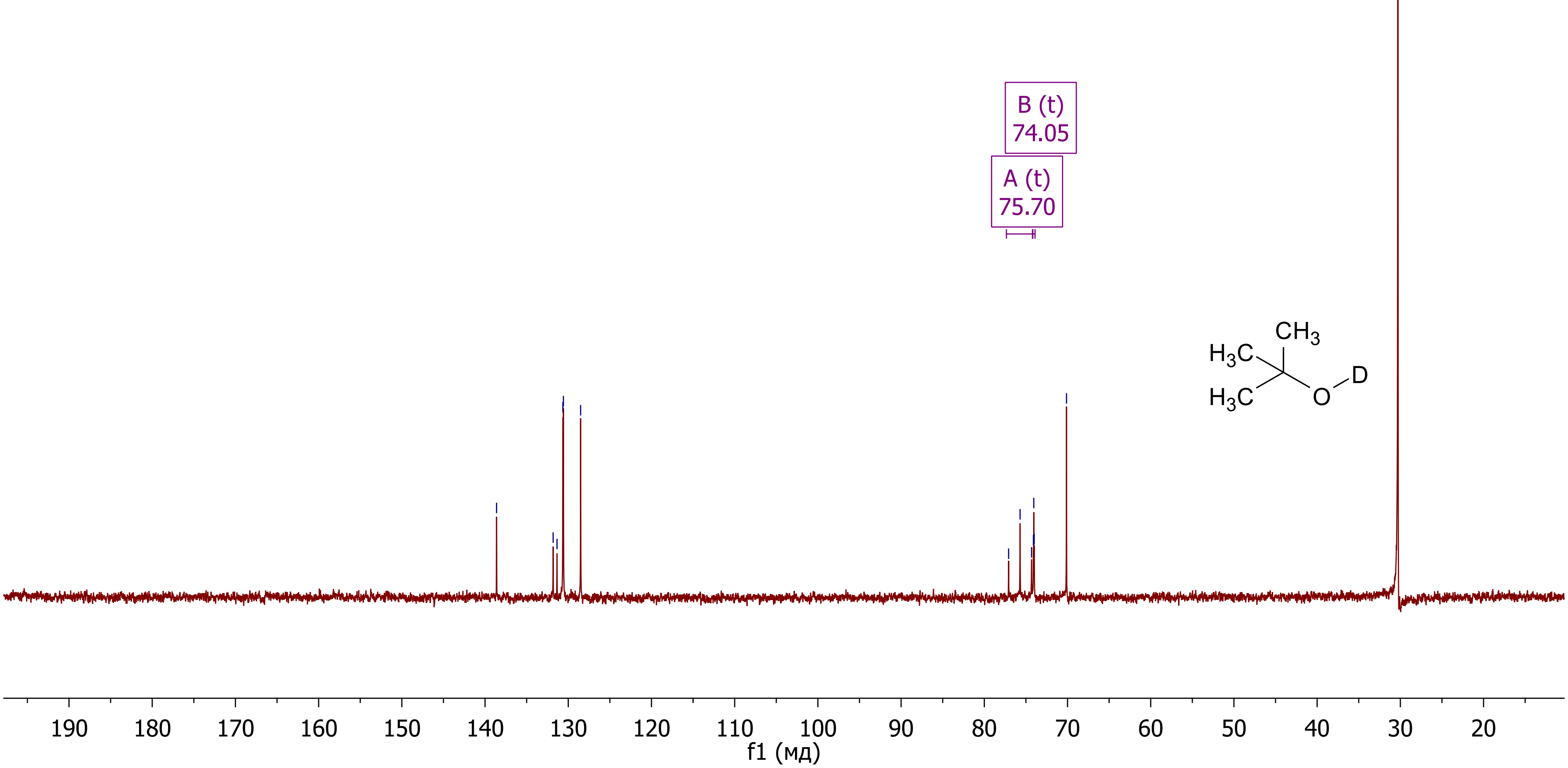

kr9943

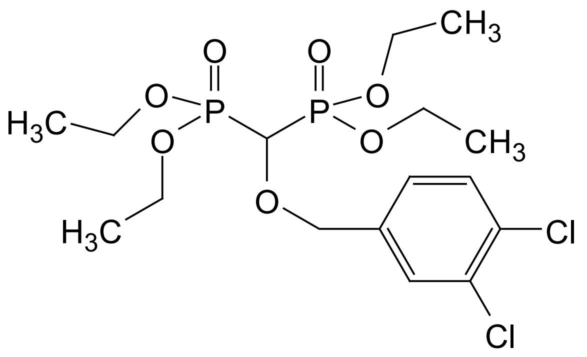

^{1}H NMR (400 MHz, $CDCl_3$) δ 7.51 (d, J = 1.9 Hz, 1H), 7.42 (d, J = 8.2 Hz, 1H), 7.23 (dd, J = 8.2, 1.9 Hz, 1H), 4.02 (t, J = 17.2 Hz, 1H), 1.35 (td, J = 7.1, 2.8 Hz, 1H).

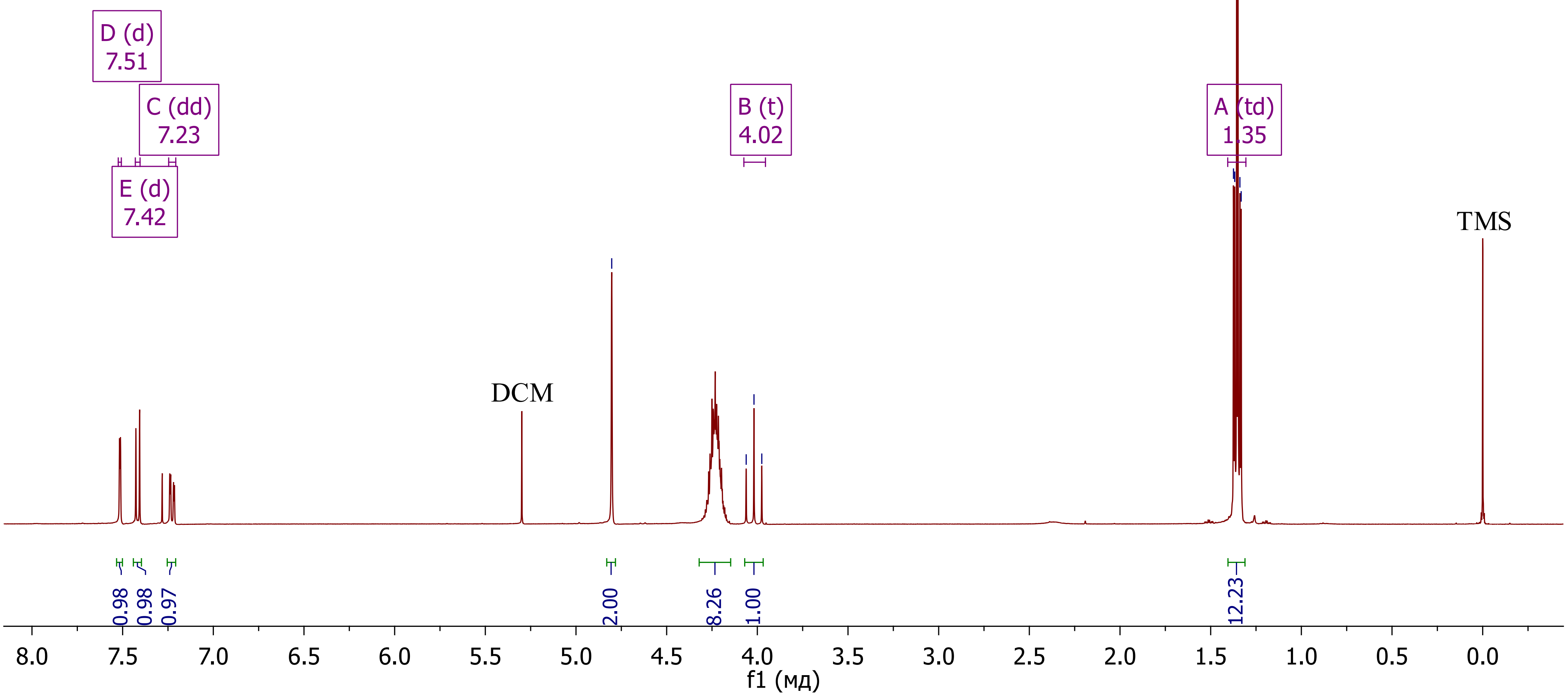

kr9943p31sup

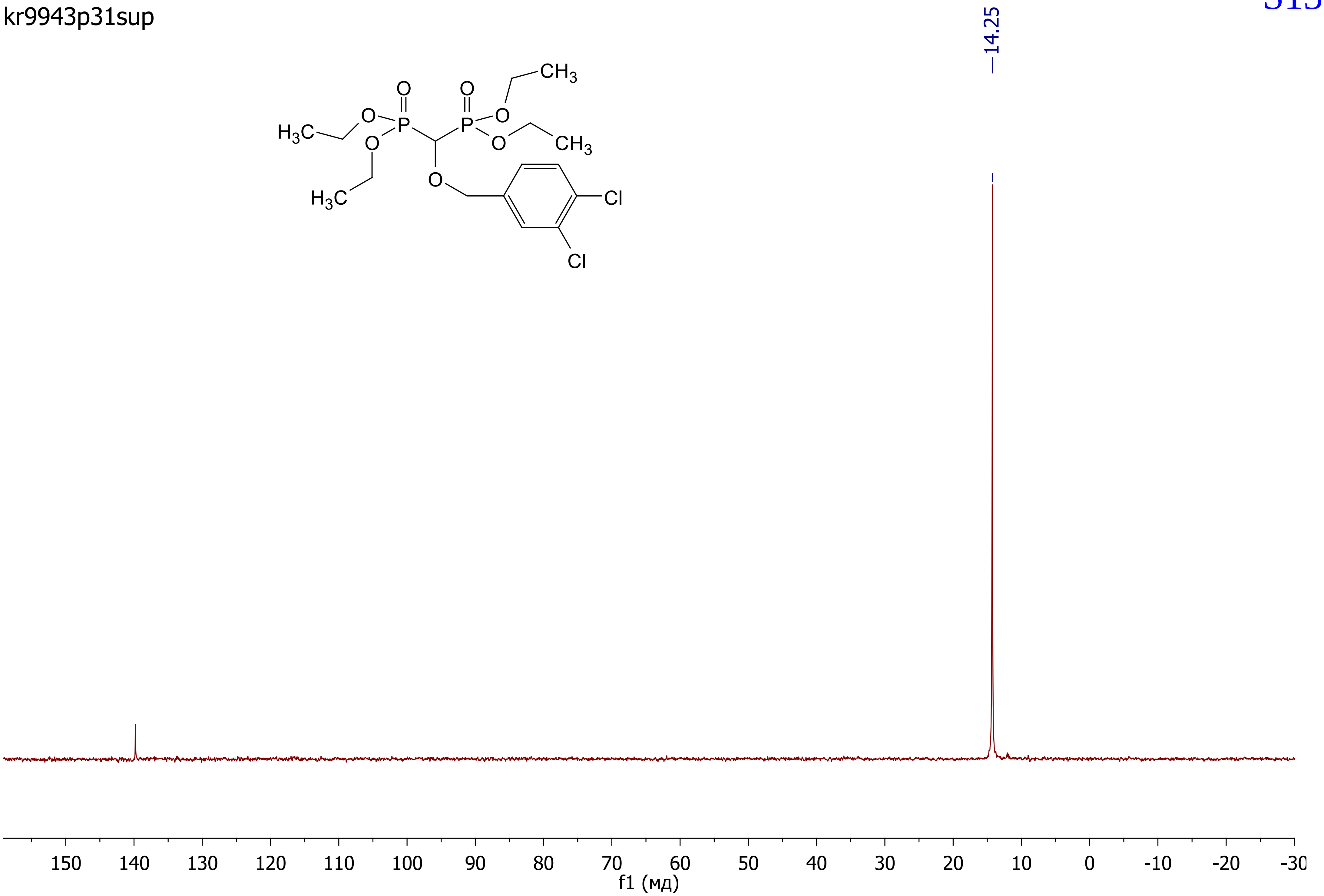

^{13}C NMR (101 MHz, $CDCl_3$) δ 74.39 (t, *J* = 5.2 Hz), 72.52 (t, *J* = 157.0 Hz).

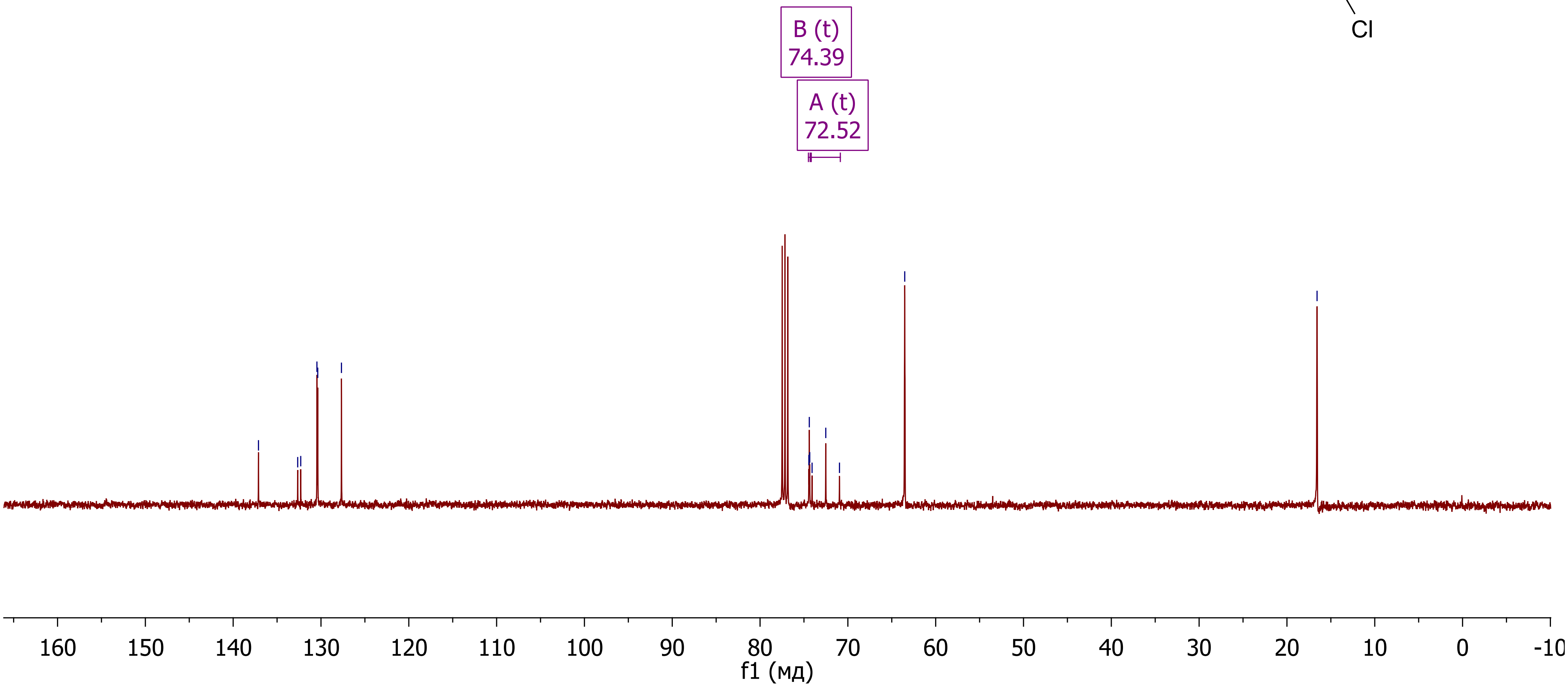

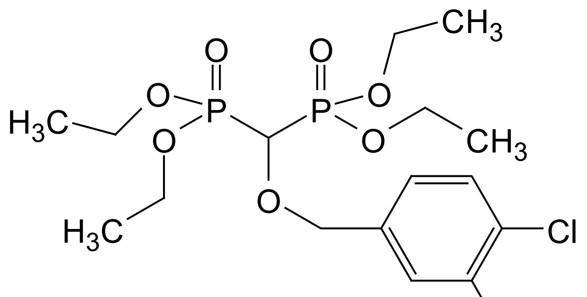

BP-3

50_F2Ph-C1-PCP-OH
solvent - D2O
pulse sequence - zgpr
number of scans - 8

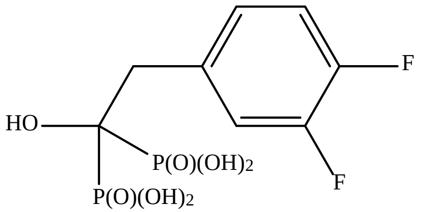

^{1}H NMR (500 MHz, Deuterium Oxide) δ 7.29 (dd, J = 8.1, 1.9 Hz, 1H), 7.18 – 7.05 (m, 4H), 3.18 (t, J = 12.6 Hz, 2H).

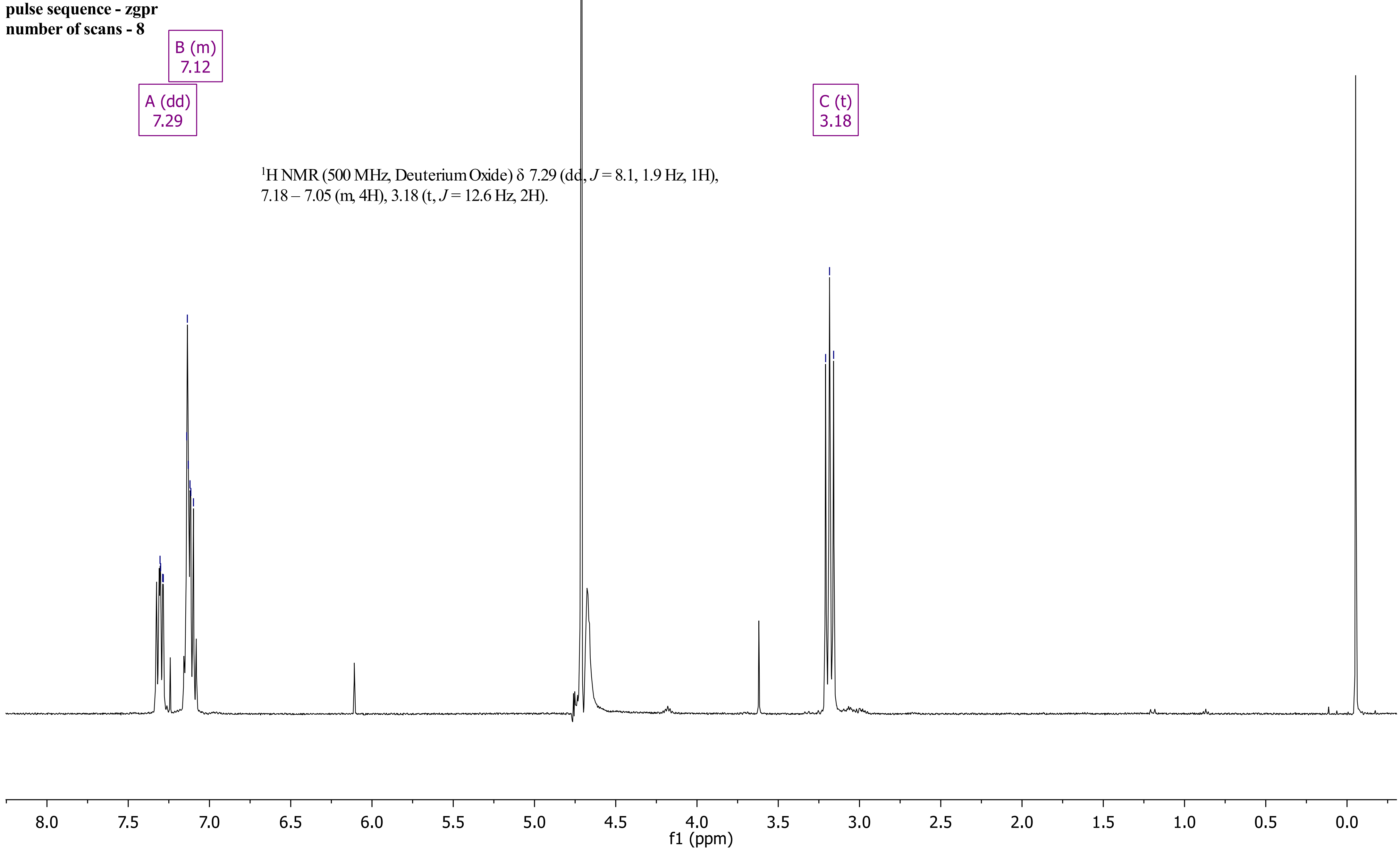

BP-3

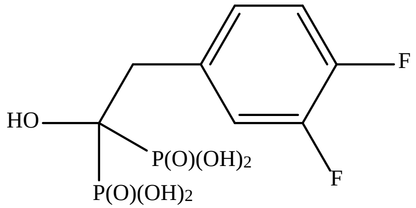

50_F2Ph-C1-PCP-OH
solvent - H2O+D2O
pulse sequence - zgdc
number of scans - 100

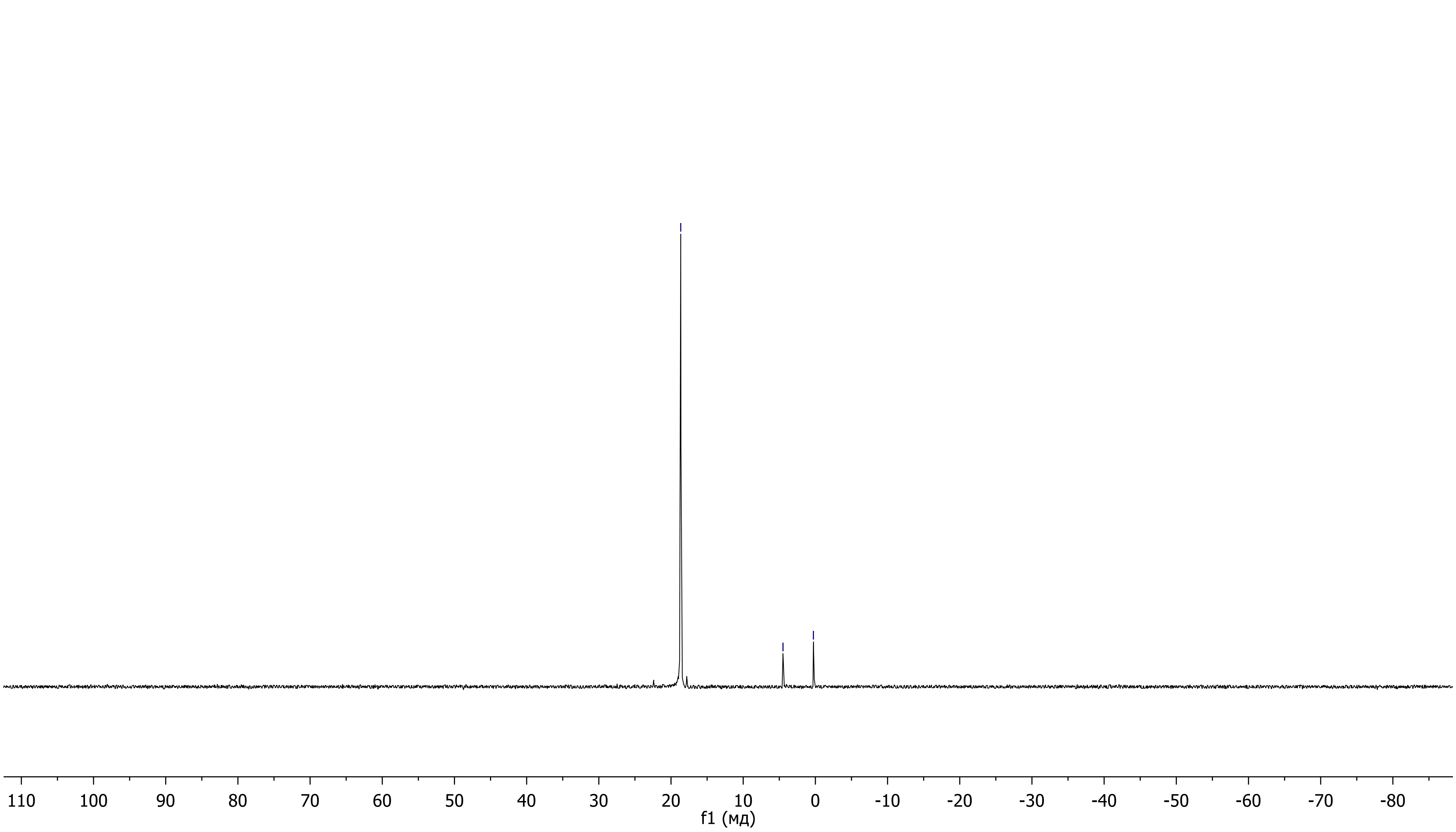

BP-3

50_F2Ph-C1-PCP-OH
solvent - H2O+D2O
pulse sequence - zgfhigqn
number of scans - 100

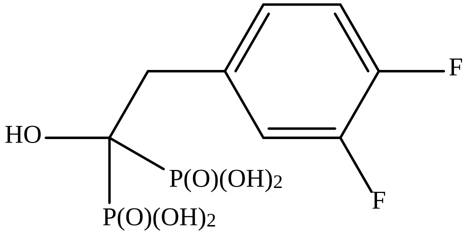

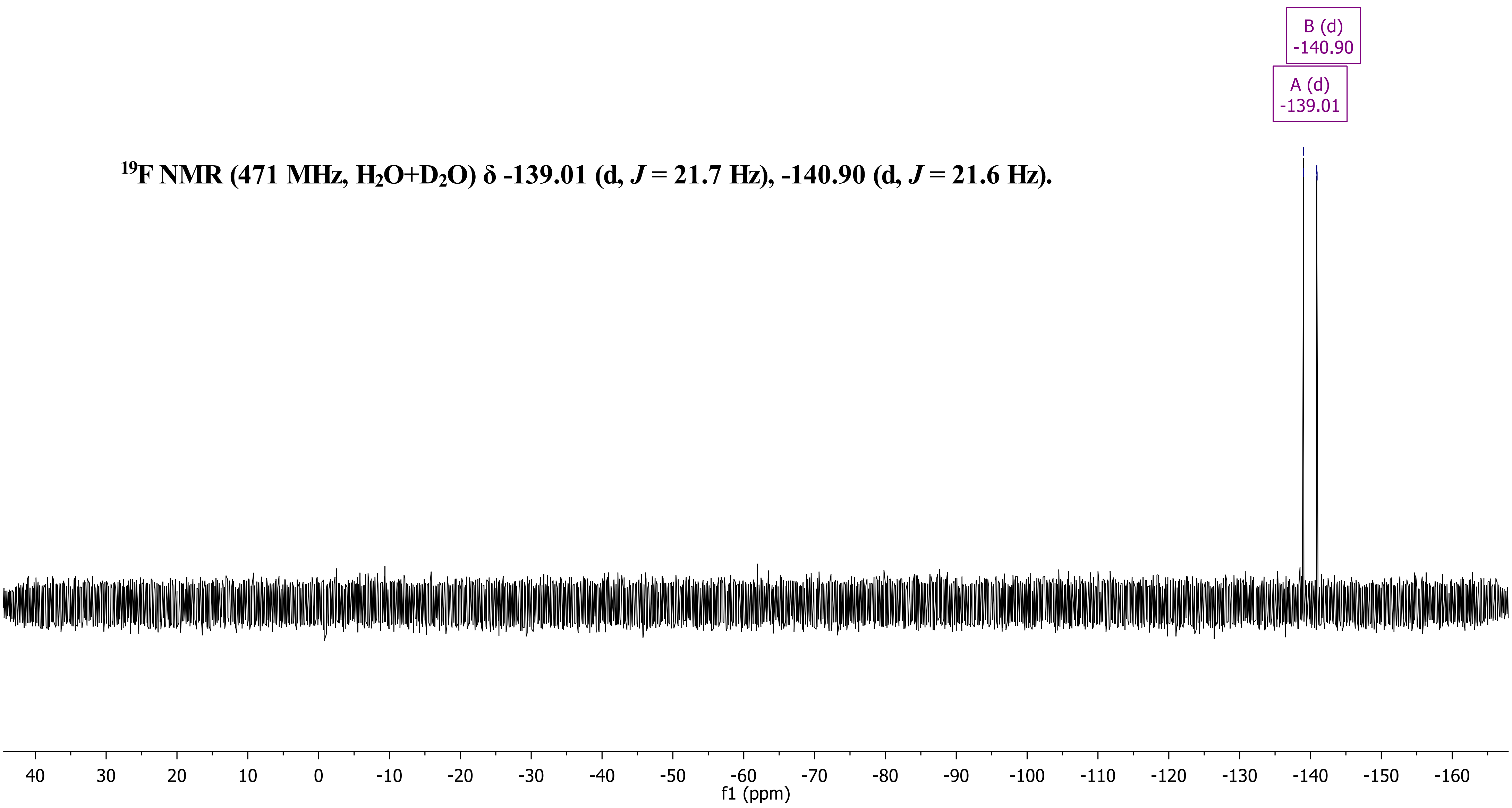

^{19}F NMR (471 MHz, H_2O+D_2O) δ -139.01 (d, *J* = 21.7 Hz), -140.90 (d, *J* = 21.6 Hz).

BP-3

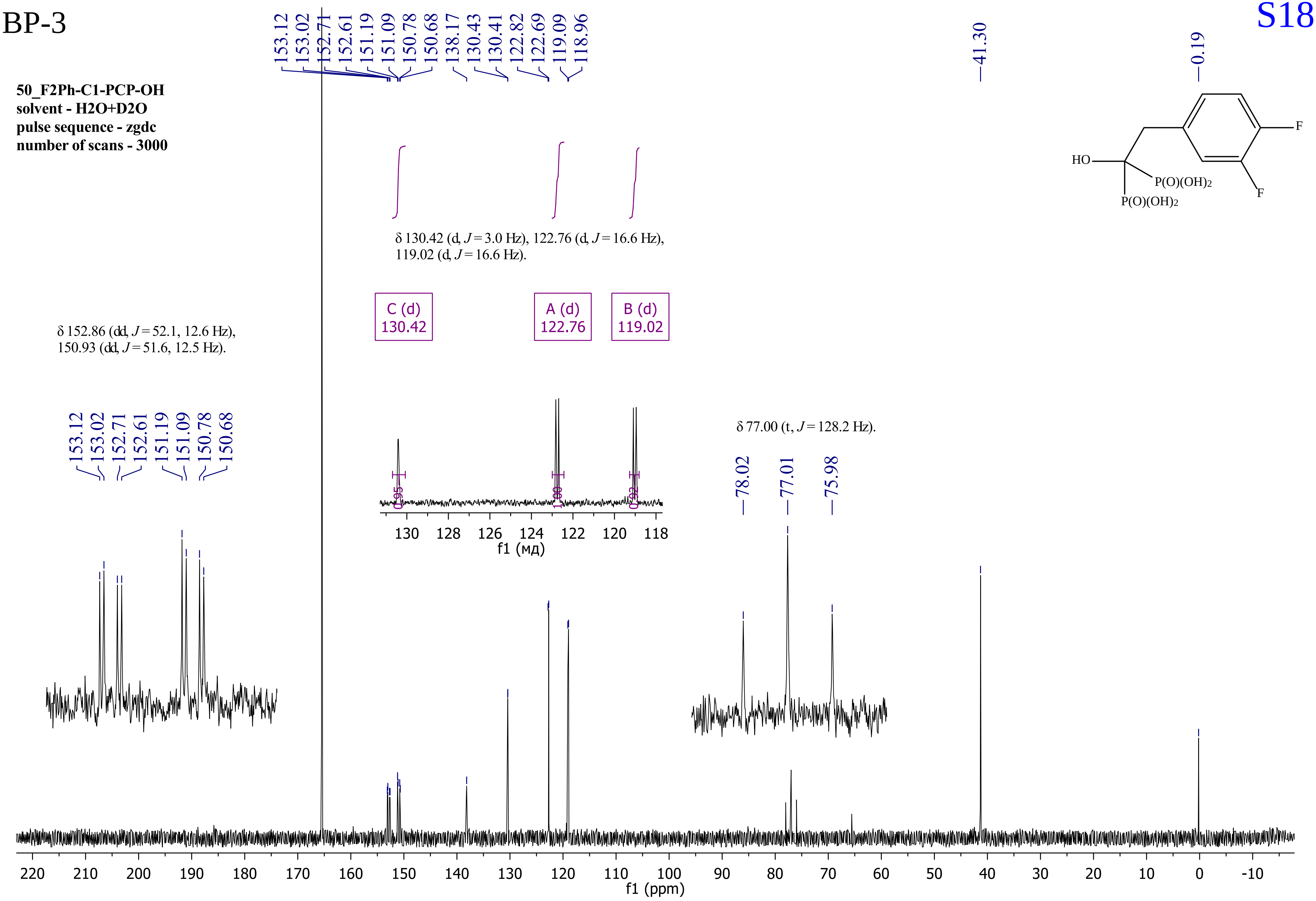
50_F2Ph-C1-PCP-OH
solvent - H2O+D2O
pulse sequence - zgdc
number of scans - 3000
153.12
153.02
152.71
152.61
151.19
151.09
150.78
150.68
138.17
130.43
130.41
122.82
122.69
119.09
118.96
41.30
0.19
δ 130.42 (d, J = 3.0 Hz), 122.76 (d, J = 16.6 Hz), 119.02 (d, J = 16.6 Hz).
C (d) 130.42
A (d) 122.76
B (d) 119.02
δ 152.86 (dd, J = 52.1, 12.6 Hz), 150.93 (dd, J = 51.6, 12.5 Hz).
δ 77.00 (t, J = 128.2 Hz).
78.02
77.01
75.98
0.95
1.00
0.92
f1 (мд)
f1 (ppm)
HO
P(O)(OH)2
P(O)(OH)2
F
F

kr9976

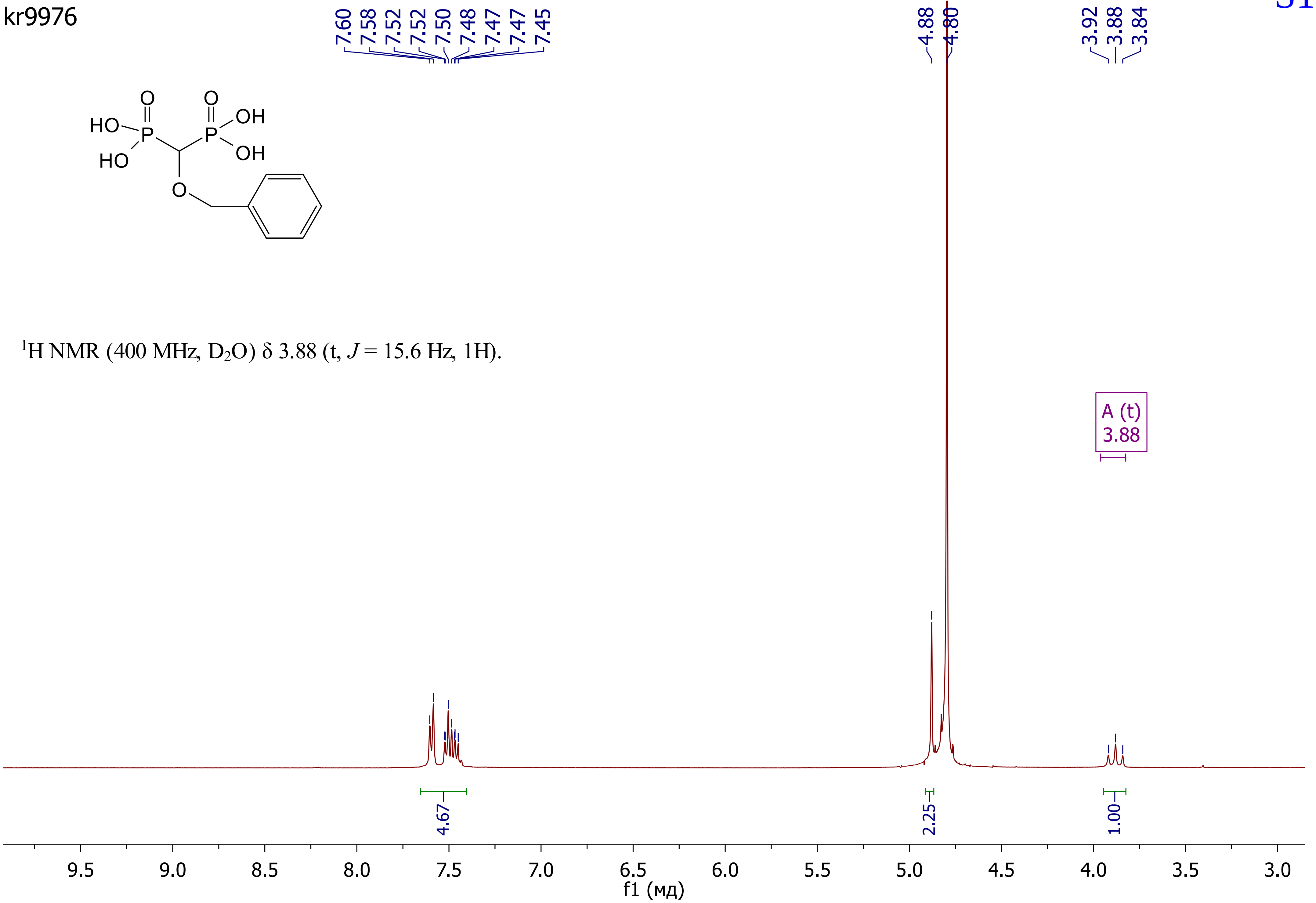

^{1}H NMR (400 MHz, D_2O) δ 3.88 (t, J = 15.6 Hz, 1H).

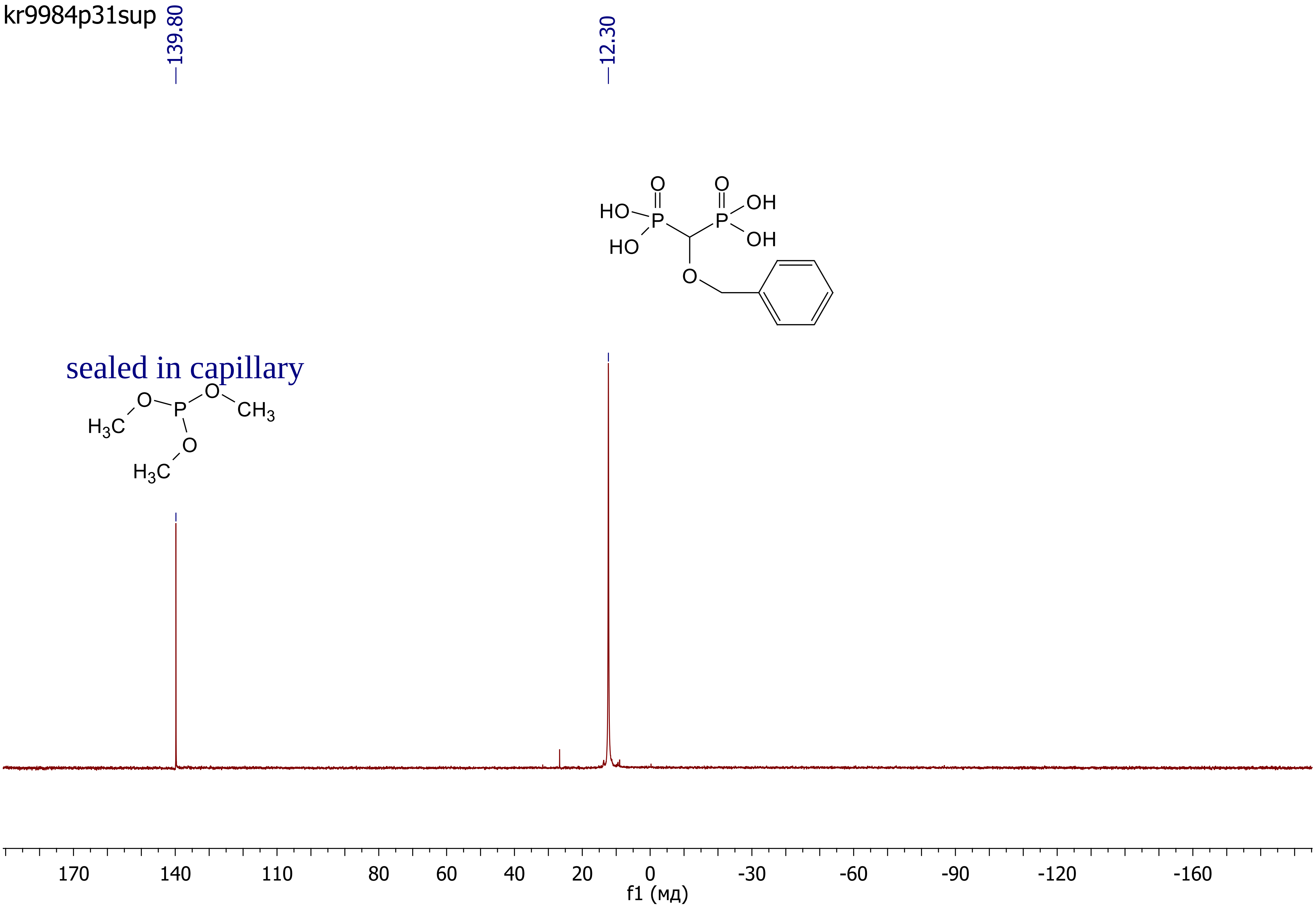
kr9984p31sup
139.80
12.30
sealed in capillary
HO
HO
O
O
P
P
OH
OH
O
O
P
O
H3C
CH3
H3C
170
140
110
80
60
40
20
0
-30
-60
-90
-120
-160
f1 (мд)

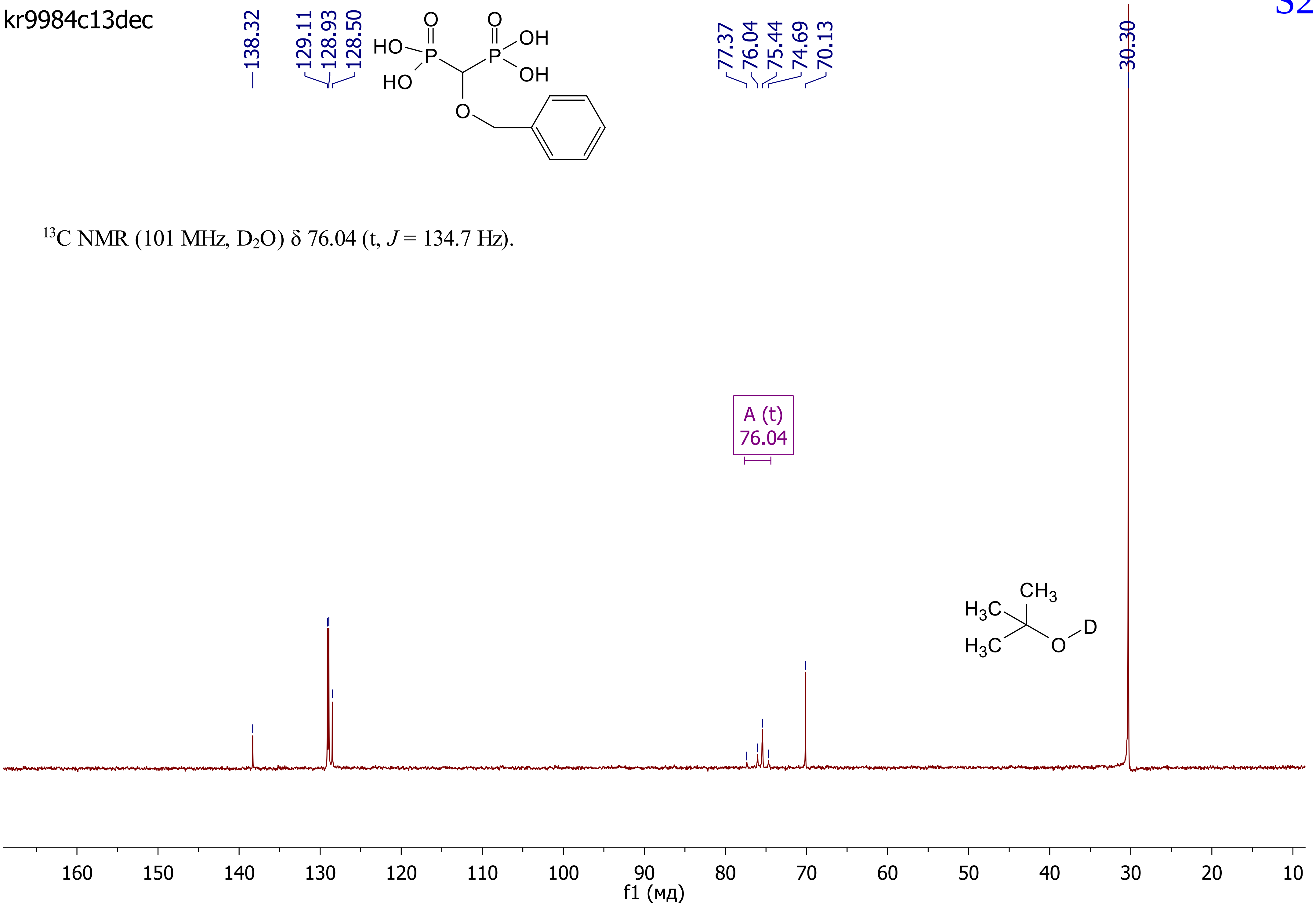

^{13}C NMR (101 MHz, D_2O) δ 76.04 (t, *J* = 134.7 Hz).

kr9906

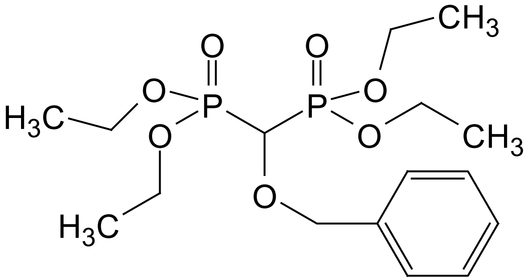

^{1}H NMR (400 MHz, CDCl$_3$) δ 3.96 (t, J = 17.2 Hz, 1H), 1.22 (td, J = 7.1, 4.7 Hz, 1H).

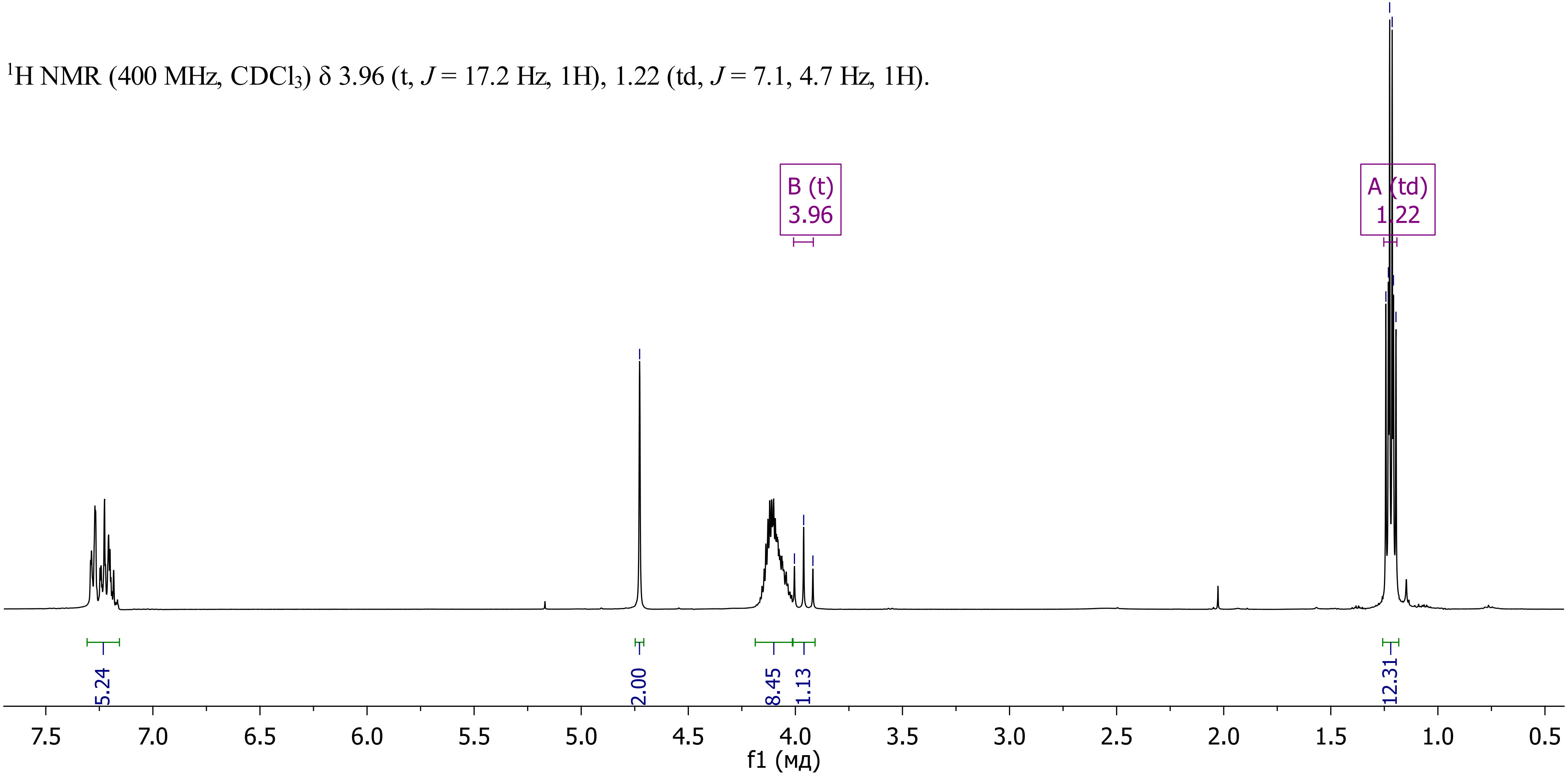

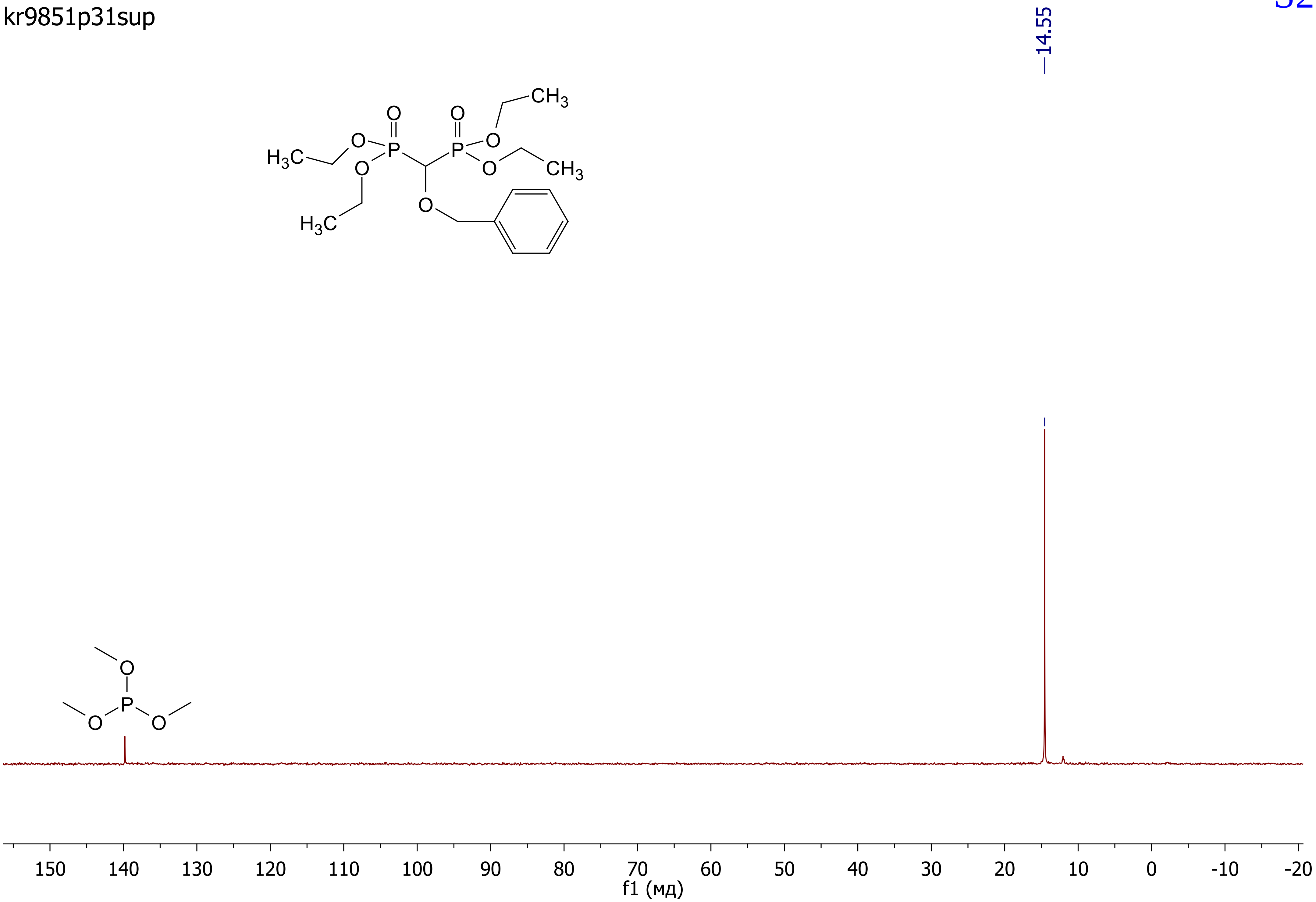
kr9851p31sup
14.55
O
O
H3C
O
P
P
O
CH3
O
O
CH3
H3C
O
O
P
O
O
150
140
130
120
110
100
90
80
70
60
50
40
30
20
10
0
-10
-20
f1 (мд)

kr9906c13dec

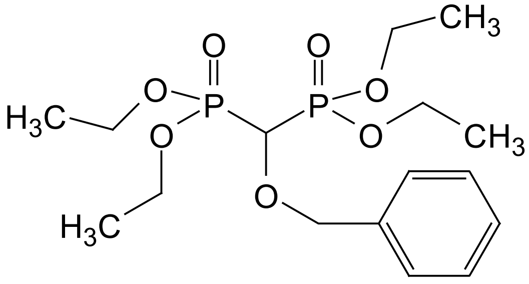

^{13}C NMR (101 MHz, $CDCl_3$) δ 75.46 (t, J = 5.0 Hz), 71.57 (t, J = 156.5 Hz), 63.06 (d, J = 14.7 Hz), 16.20 (d, J = 2.2 Hz).

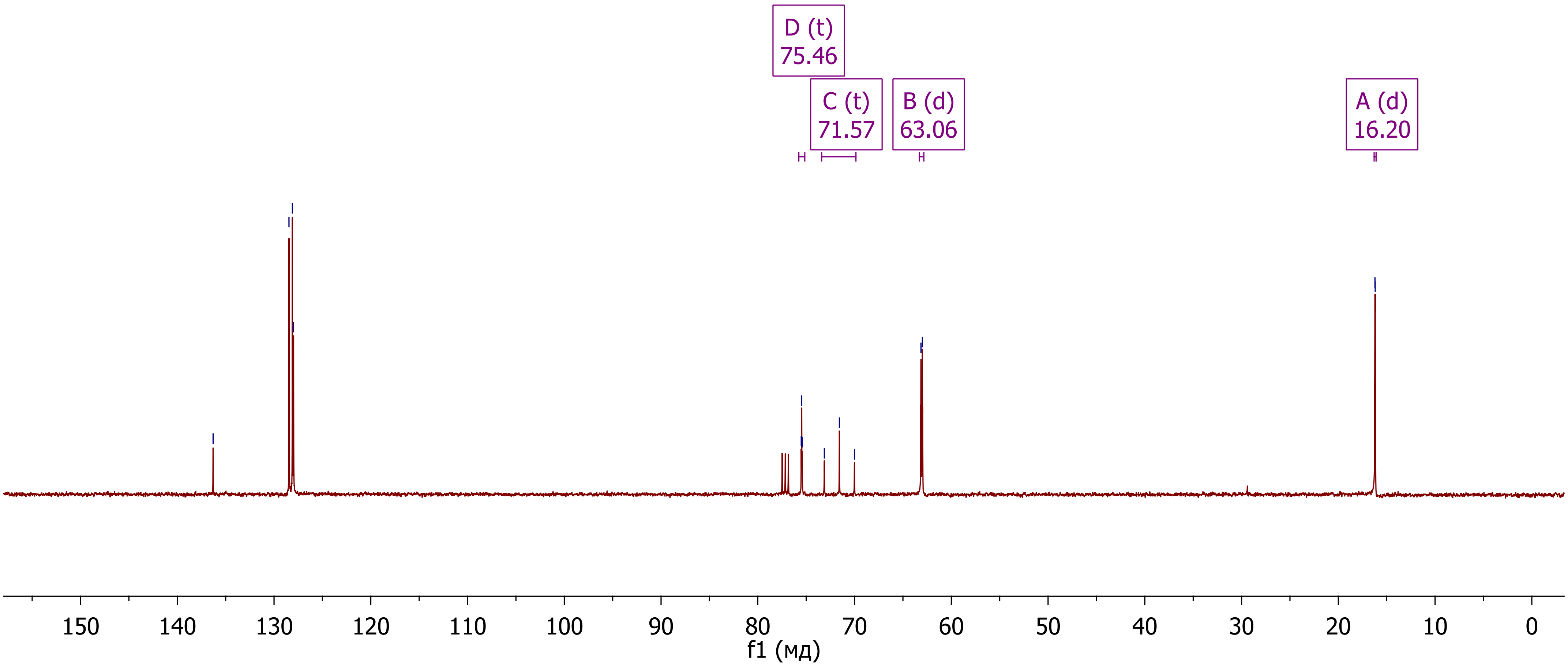

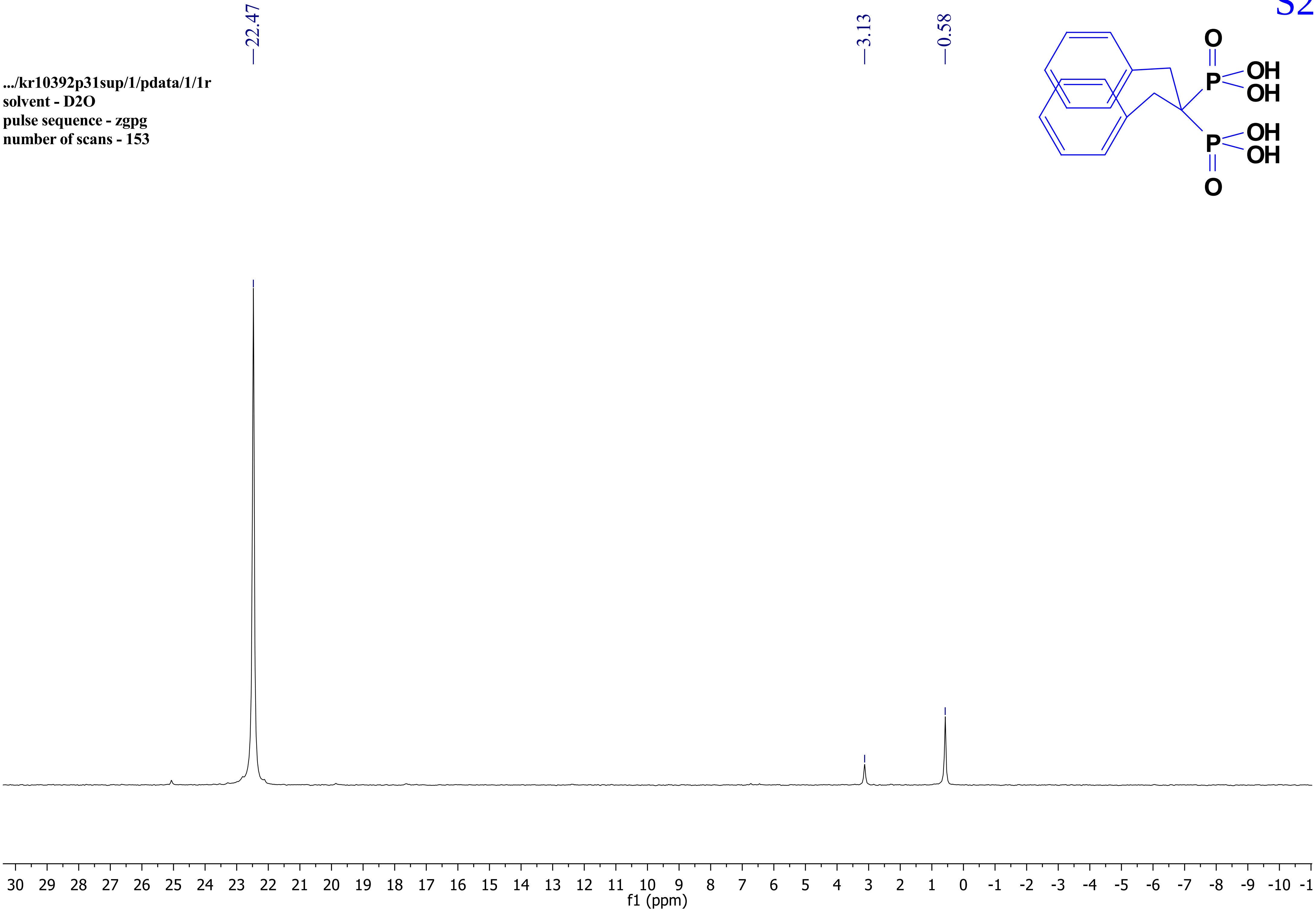
22.47
3.13
0.58
.../kr10392p31sup/1/pdata/1/1r
solvent - D2O
pulse sequence - zgpg
number of scans - 153
O
P
OH
OH
OH
OH
P
O
30 29 28 27 26 25 24 23 22 21 20 19 18 17 16 15 14 13 12 11 10 9 8 7 6 5 4 3 2 1 0 -1 -2 -3 -4 -5 -6 -7 -8 -9 -10 -1
f1 (ppm)

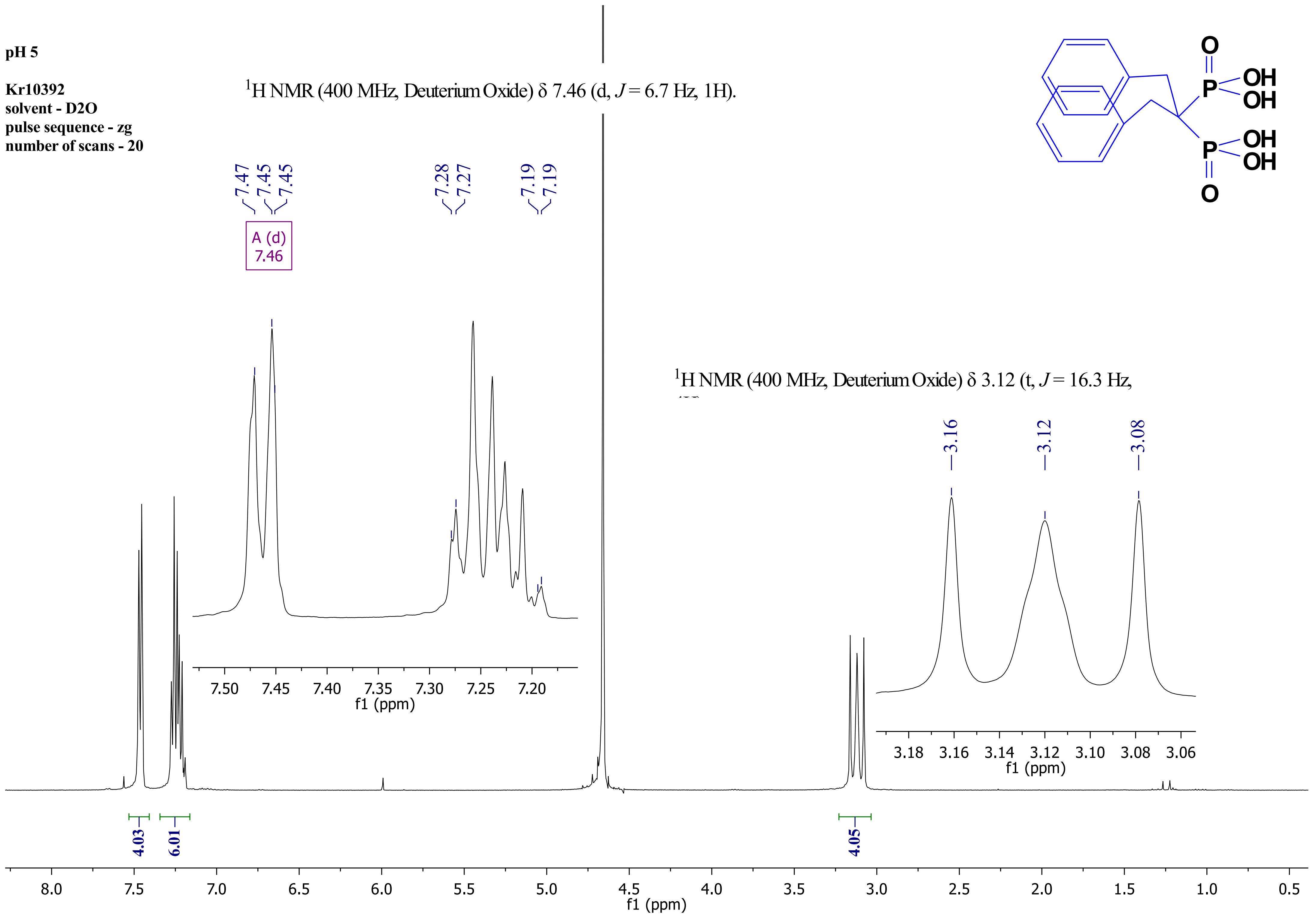
pH 5
Kr10392
solvent - D2O
pulse sequence - zg
number of scans - 20
^{1}H NMR (400 MHz, Deuterium Oxide) δ 7.46 (d, J = 6.7 Hz, 1H).
7.47
7.45
7.45
7.28
7.27
7.19
7.19
A (d)
7.46
^{1}H NMR (400 MHz, Deuterium Oxide) δ 3.12 (t, J = 16.3 Hz,
3.16
3.12
3.08
4.03
6.01
4.05
f1 (ppm)
O
P
OH
OH
P
OH
OH
O

kr10392-c13dec-2DAYS
D2O
num of scans: 22042

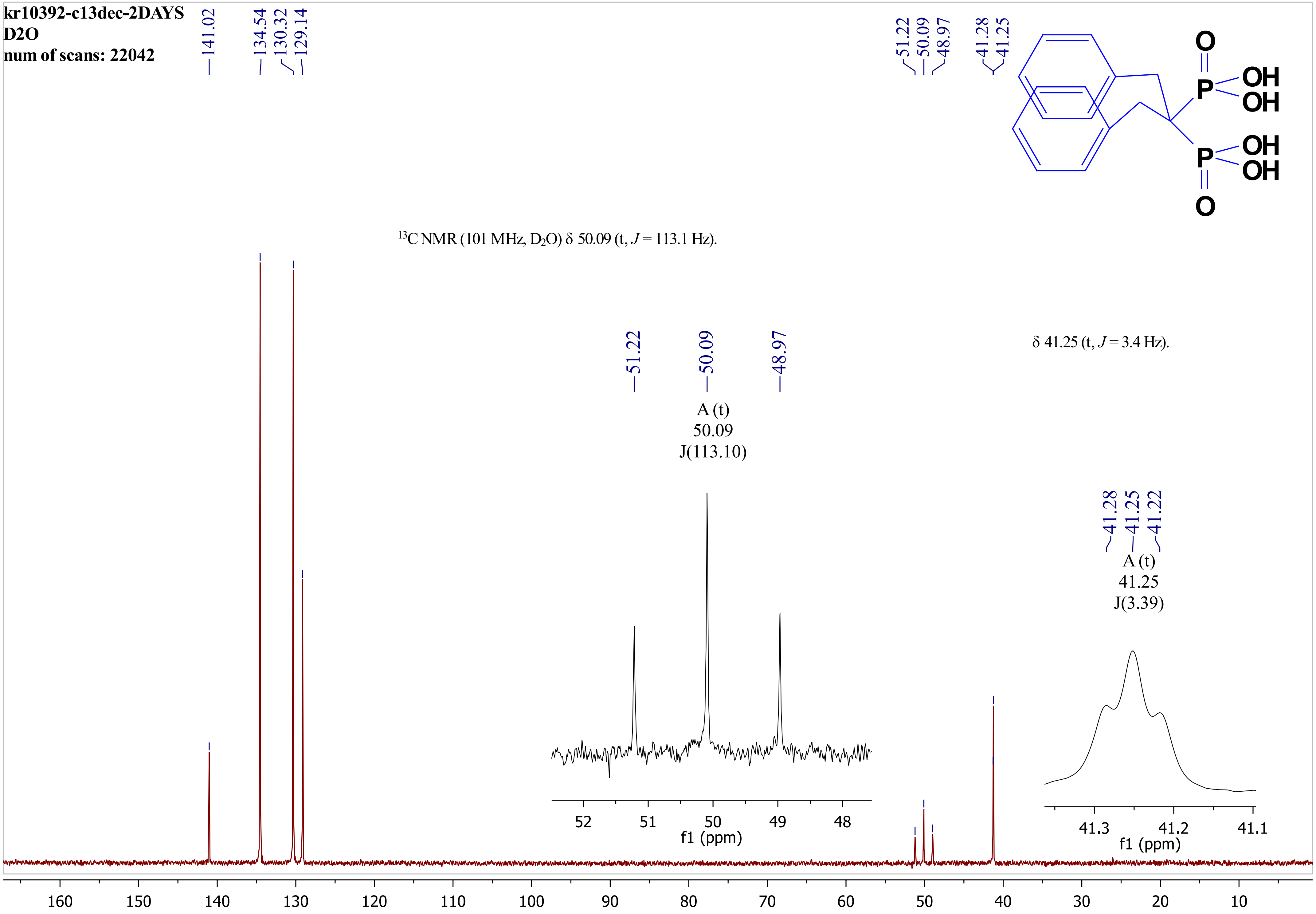

kr9714
solvent - CDCl3
pulse sequence - zg
number of scans - 8

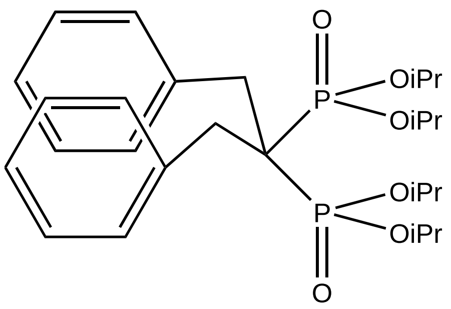

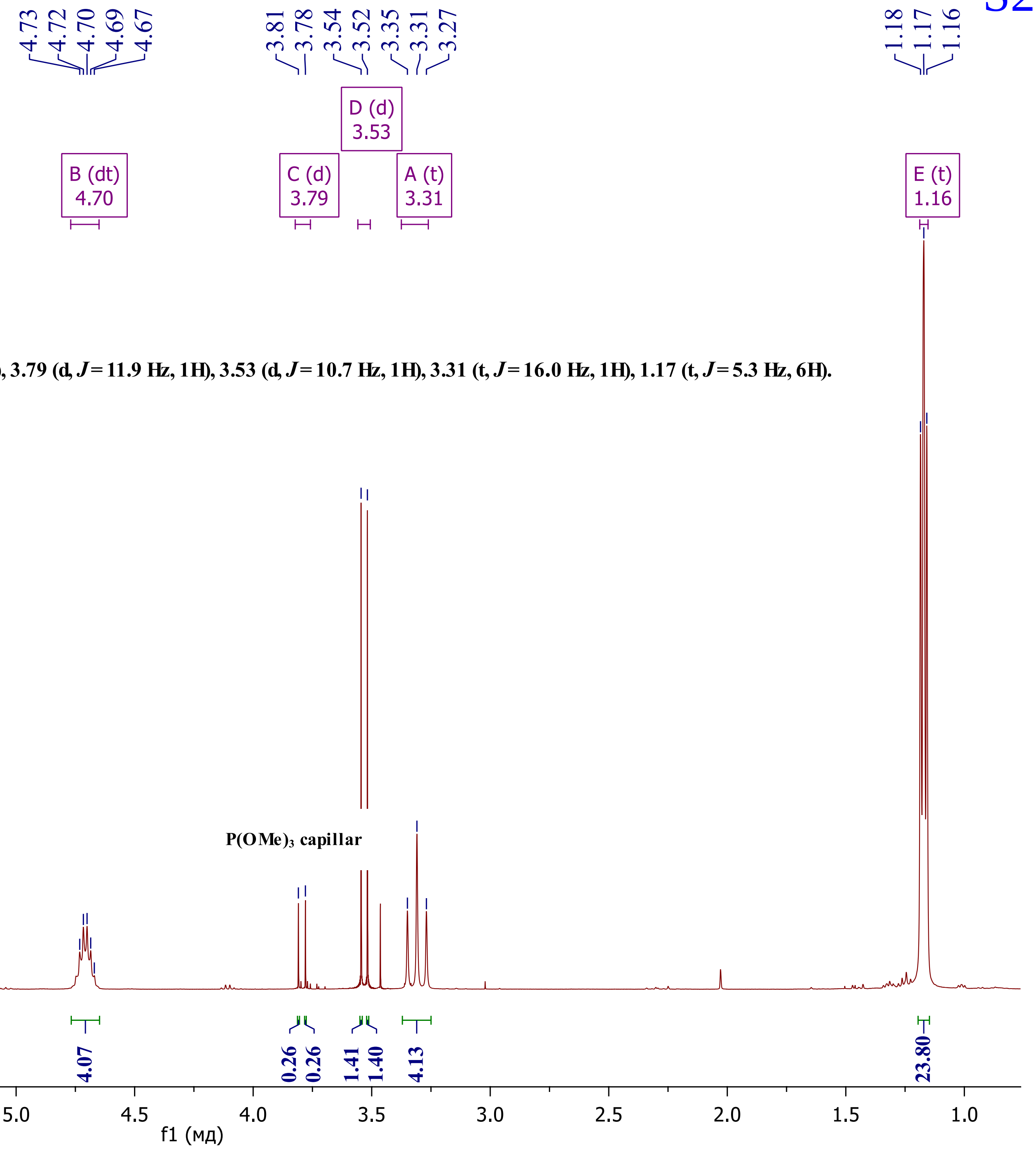

^{1}H NMR (400 MHz, $CDCl_3$) δ 4.70 (dt, J = 12.3, 6.1 Hz, 1H), 3.79 (d, J = 11.9 Hz, 1H), 3.53 (d, J = 10.7 Hz, 1H), 3.31 (t, J = 16.0 Hz, 1H), 1.17 (t, J = 5.3 Hz, 6H).

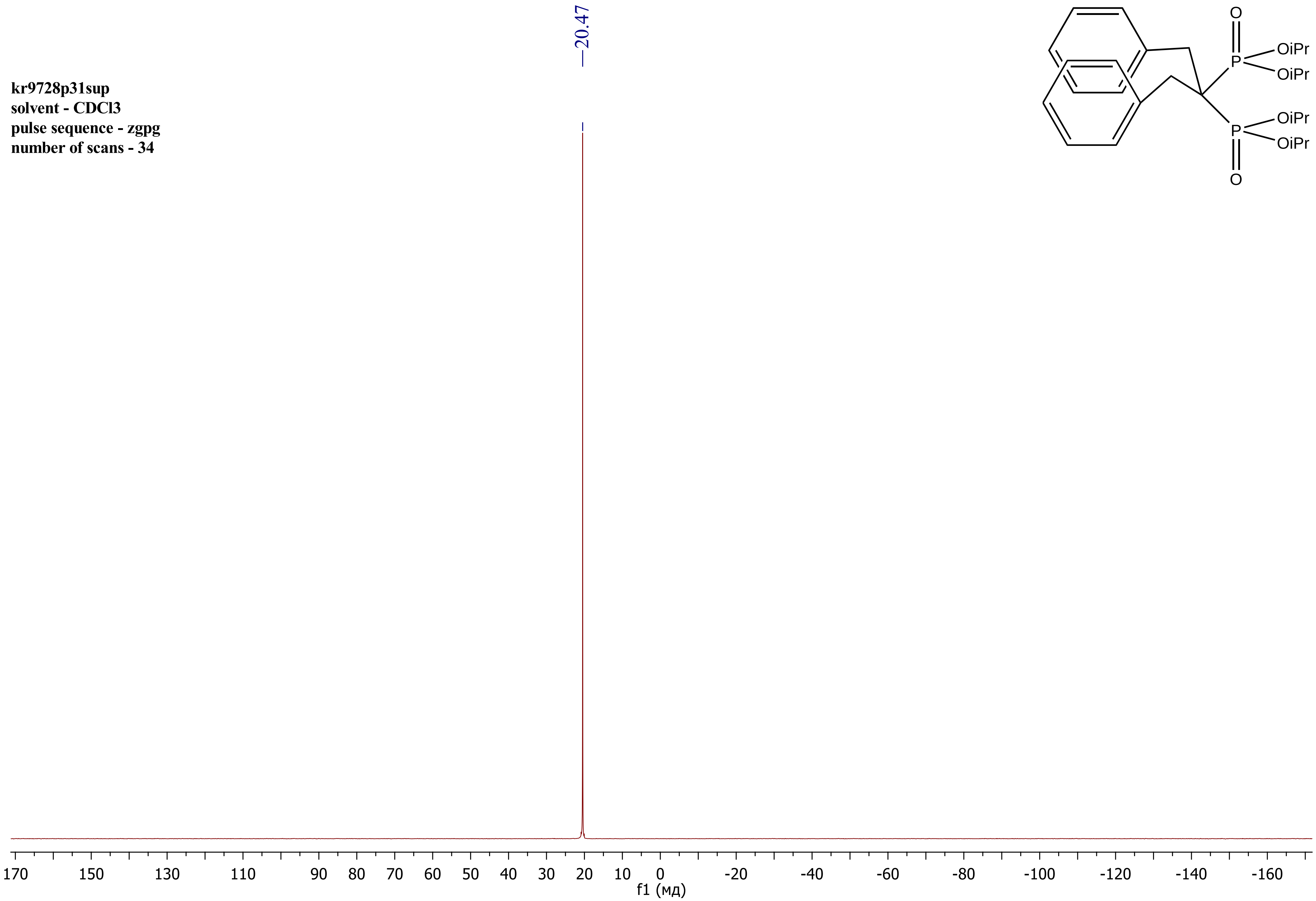
kr9728p31sup
solvent - CDCl3
pulse sequence - zgpg
number of scans - 34
20.47
170 150 130 110 90 80 70 60 50 40 30 20 10 0 -20 -40 -60 -80 -100 -120 -140 -160
f1 (мд)
O
OiPr
OiPr
P
OiPr
OiPr
P
O

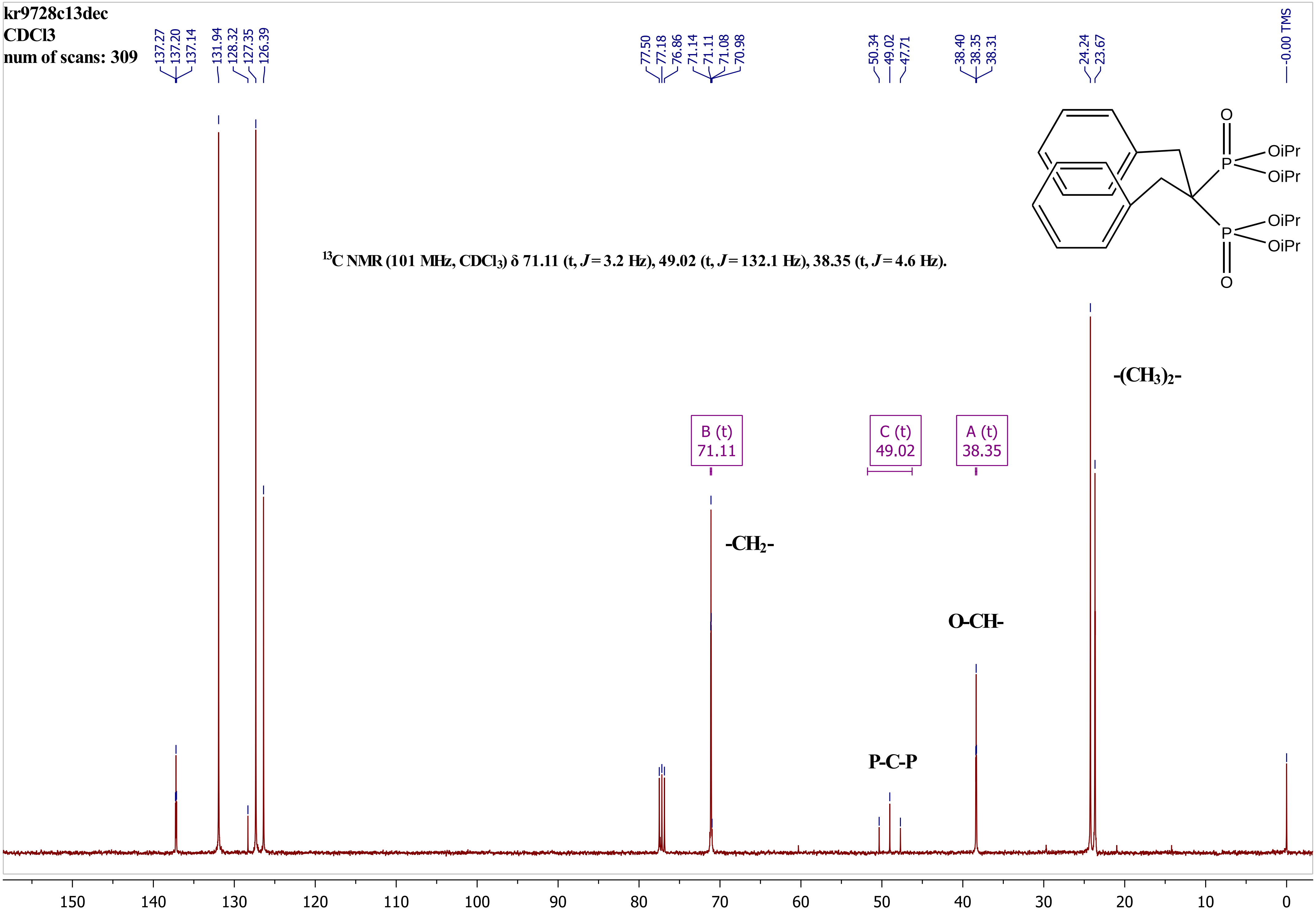
kr9728c13dec
CDCl3
num of scans: 309
137.27
137.20
137.14
131.94
128.32
127.35
126.39
77.50
77.18
76.86
71.14
71.11
71.08
70.98
50.34
49.02
47.71
38.40
38.35
38.31
24.24
23.67
-0.00 TMS
O
P
OiPr
OiPr
OiPr
OiPr
P
O
13C NMR (101 MHz, CDCl3) δ 71.11 (t, J = 3.2 Hz), 49.02 (t, J = 132.1 Hz), 38.35 (t, J = 4.6 Hz).
-(CH3)2-
B (t)
71.11
C (t)
49.02
A (t)
38.35
-CH2-
O-CH-
P-C-P
150
140
130
120
110
100
90
80
70
60
50
40
30
20
10
0

BP-6

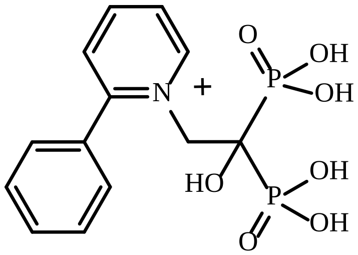

pulse sequence - zgpg
number of scans - 90

Kr 10.433
^{1}H
D_2O, pH 2

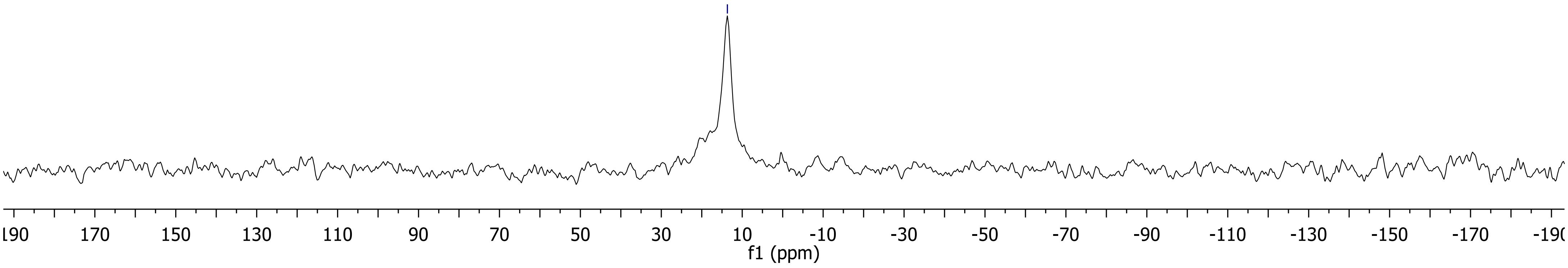

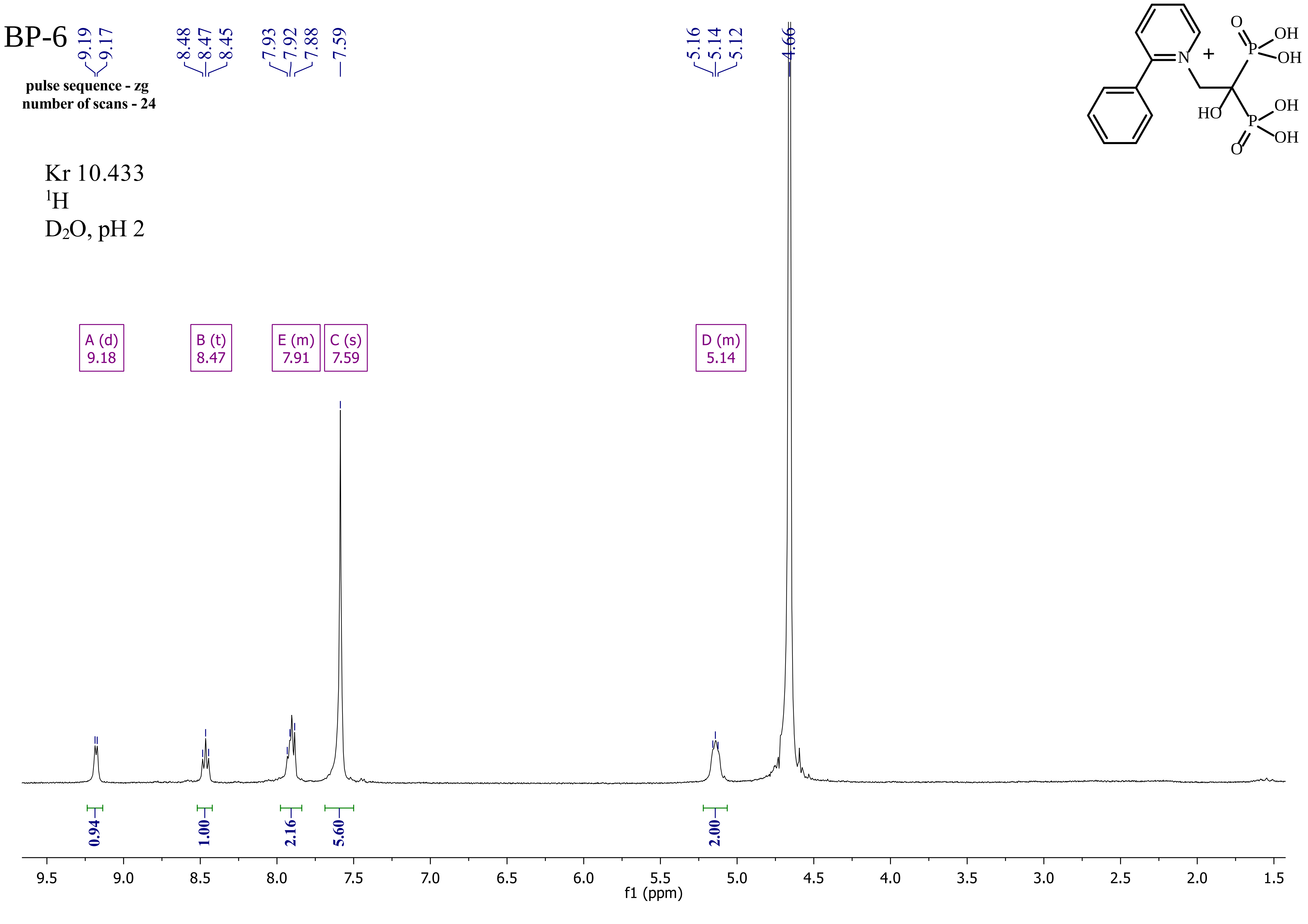

BP-6
9.19
9.17
8.48
8.47
8.45
7.93
7.92
7.88
7.59
5.16
5.14
5.12
4.66
pulse sequence - zg
number of scans - 24
Kr 10.433
¹H
D₂O, pH 2
A (d)
9.18
B (t)
8.47
E (m)
7.91
C (s)
7.59
D (m)
5.14
0.94
1.00
2.16
5.60
2.00
9.5
9.0
8.5
8.0
7.5
7.0
6.5
6.0
5.5
5.0
4.5
4.0
3.5
3.0
2.5
2.0
1.5
f1 (ppm)
N
+
O
P
OH
OH
HO
OH
P
OH
O

Kr-7158; D2O; pH~1
1H

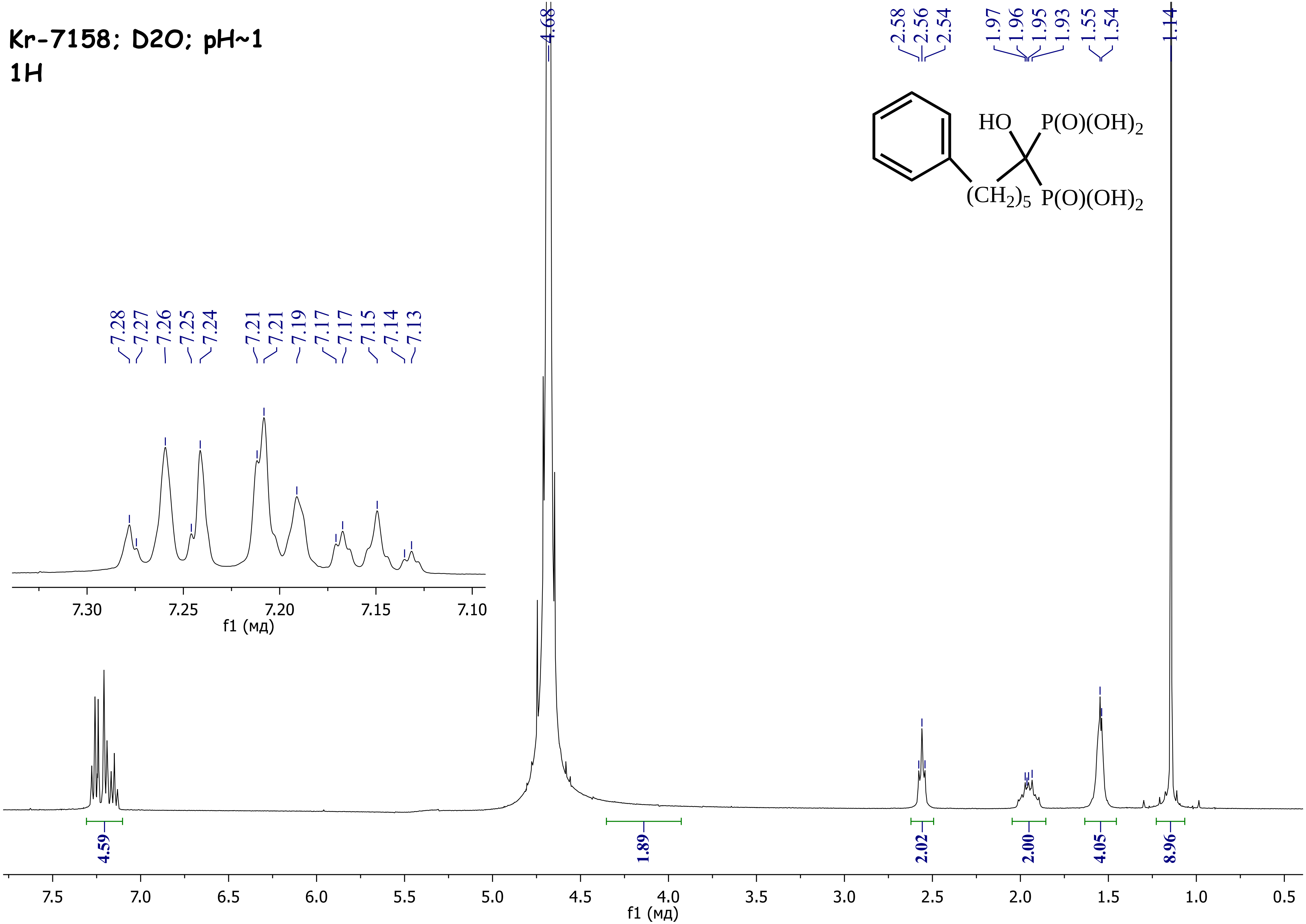

Kr 7158 D2O pH 1

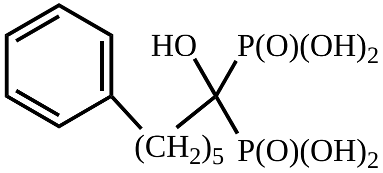

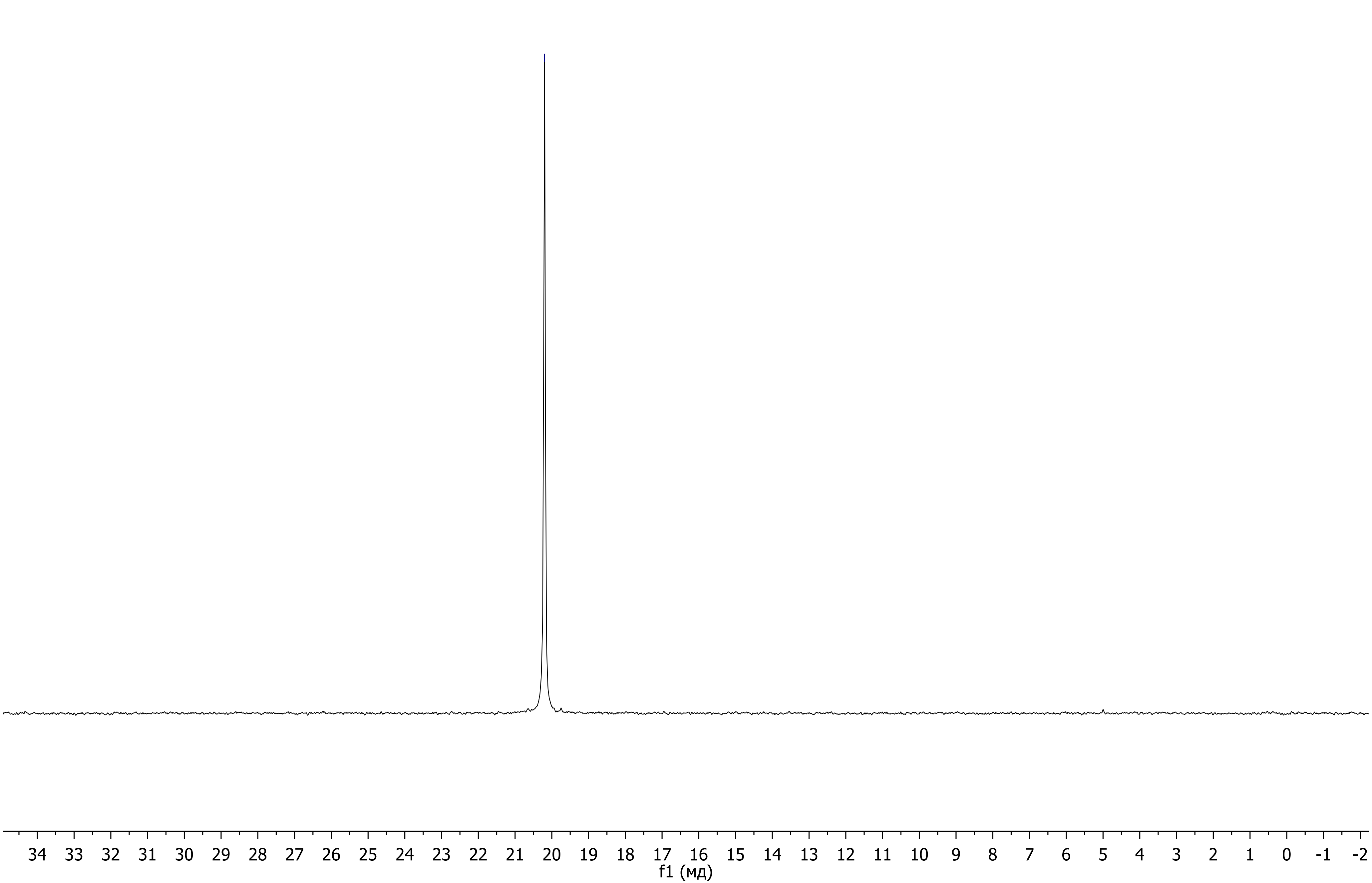

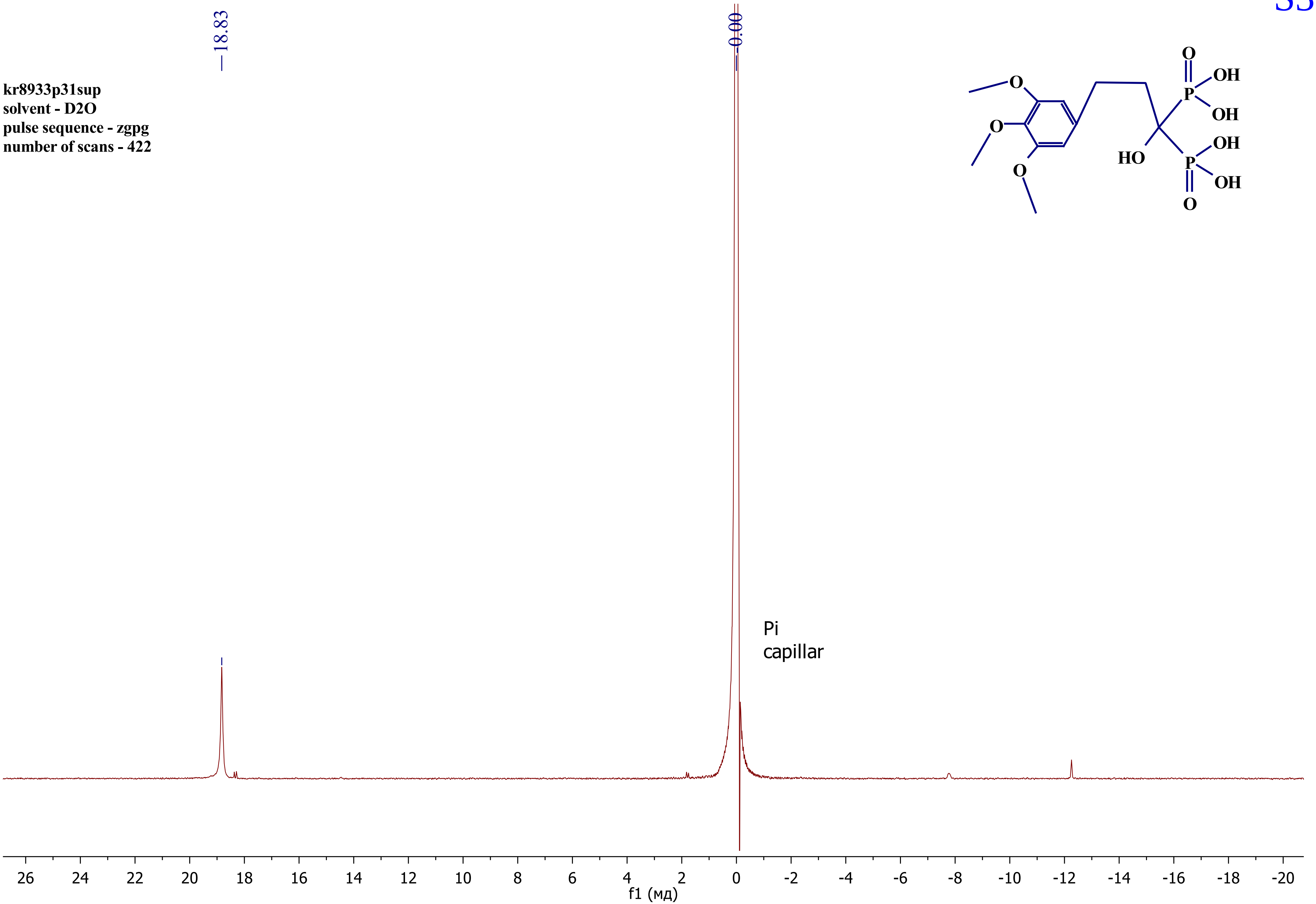
18.83
0.00
kr8933p31sup
solvent - D2O
pulse sequence - zgpg
number of scans - 422
O
OH
P
OH
O
O
HO
OH
P
OH
O
O
Pi
capillar
26 24 22 20 18 16 14 12 10 8 6 4 2 0 -2 -4 -6 -8 -10 -12 -14 -16 -18 -20
f1 (мд)

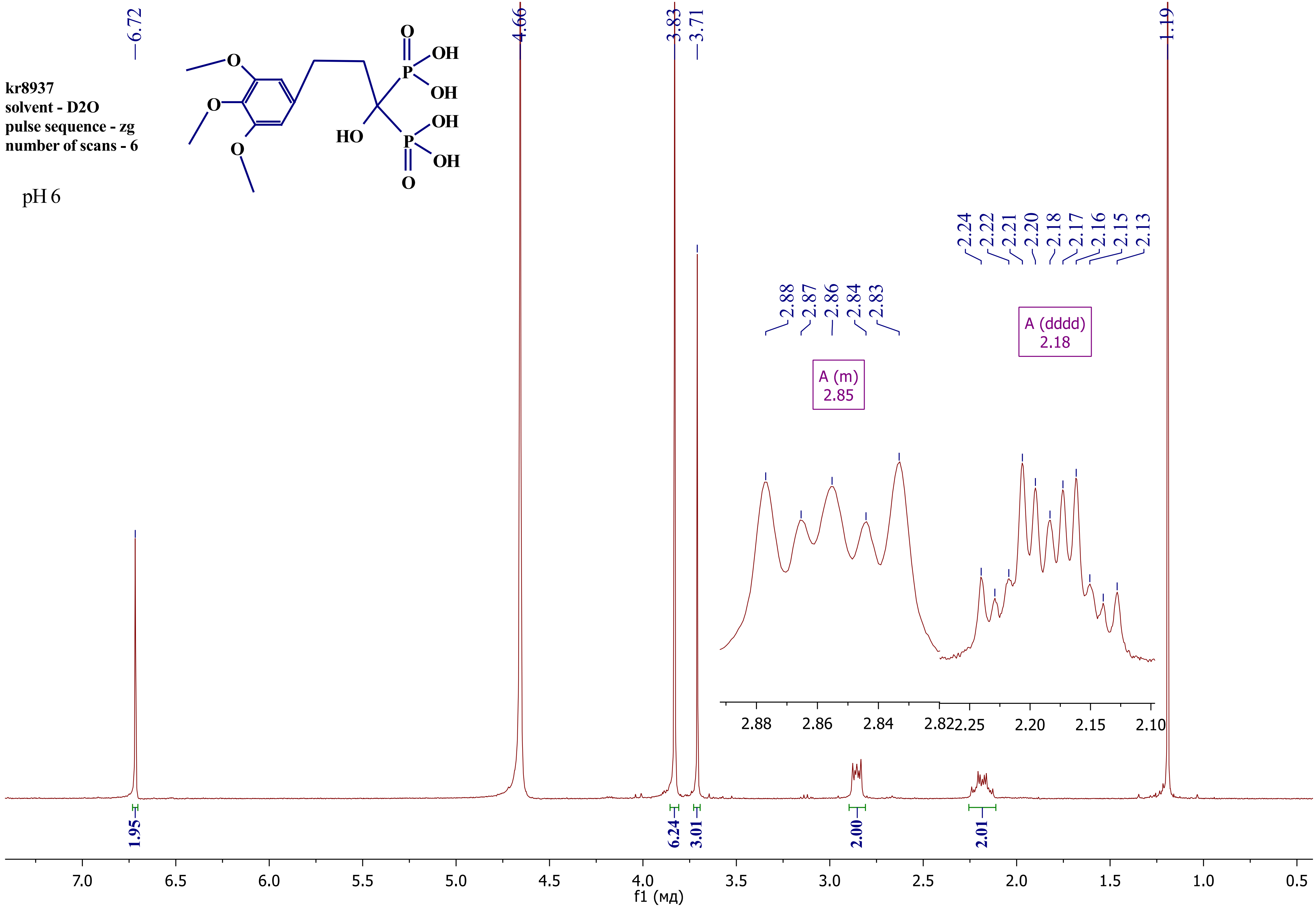
kr8937
solvent - D2O
pulse sequence - zg
number of scans - 6
pH 6
HO
OH
OH
OH
OH
O
O
O
O
O
P
P
6.72
4.66
3.83
3.71
1.19
2.88
2.87
2.86
2.84
2.83
2.24
2.22
2.21
2.20
2.18
2.17
2.16
2.15
2.13
A (m)
2.85
A (dddd)
2.18
1.95
6.24
3.01
2.00
2.01
f1 (мд)

kr8937c13dec
solvent - D2O
pulse sequence - zgpg
number of scans - 2015

pH 6

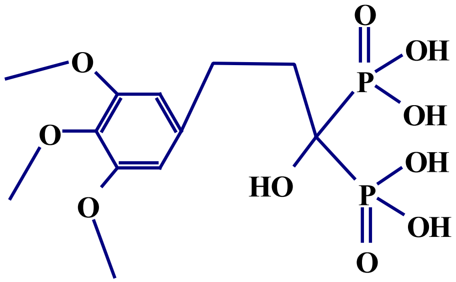

^{13}C NMR (101 MHz, D_2O) δ 76.74 (t, J = 134.0 Hz).

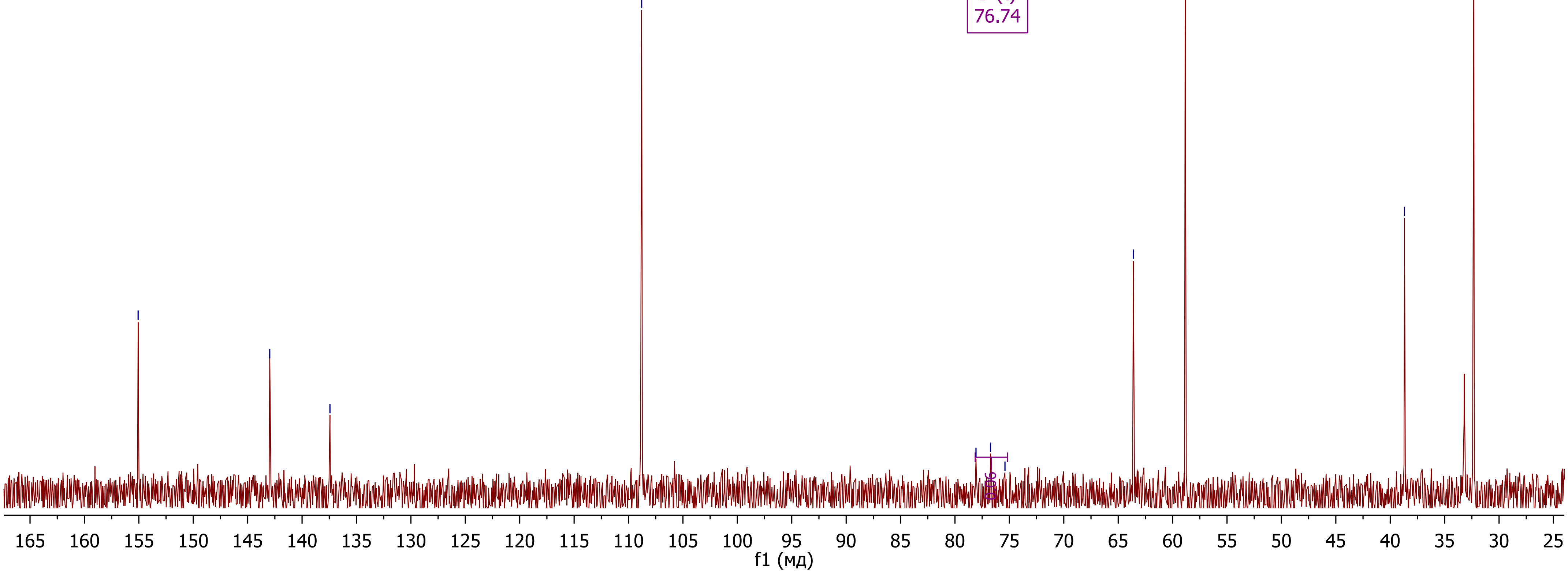

BP-11

pulse sequence - zg
number of scans - 8
Finn_Dima_781
pH 1

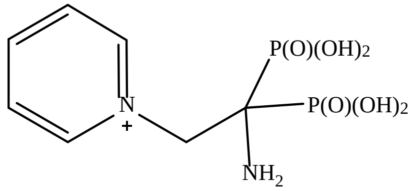

^{1}H NMR (500 MHz, Deuterium Oxide) δ 8.98 (d, J = 6.1 Hz, 1H), 8.62 (t, J = 7.9 Hz, 0H), 8.09 (t, J = 7.2 Hz, 1H), 5.24 (t, J = 9.8 Hz, 1H).

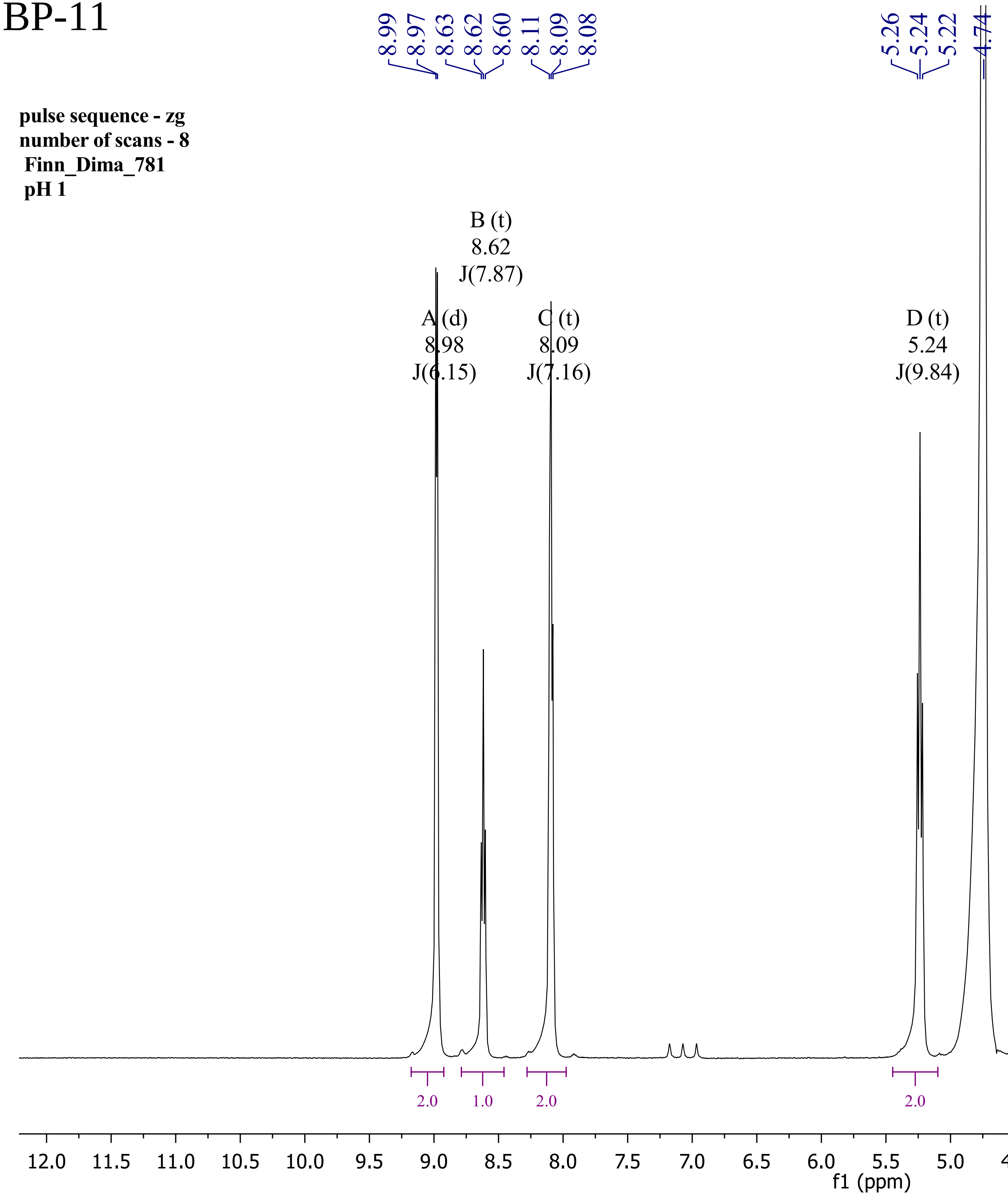

BP-11

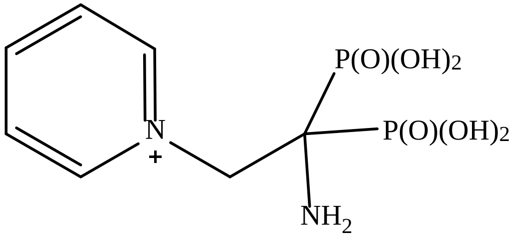

pulse sequence - zgdc
number of scans - 22
Finn_Dima_786
pH 1

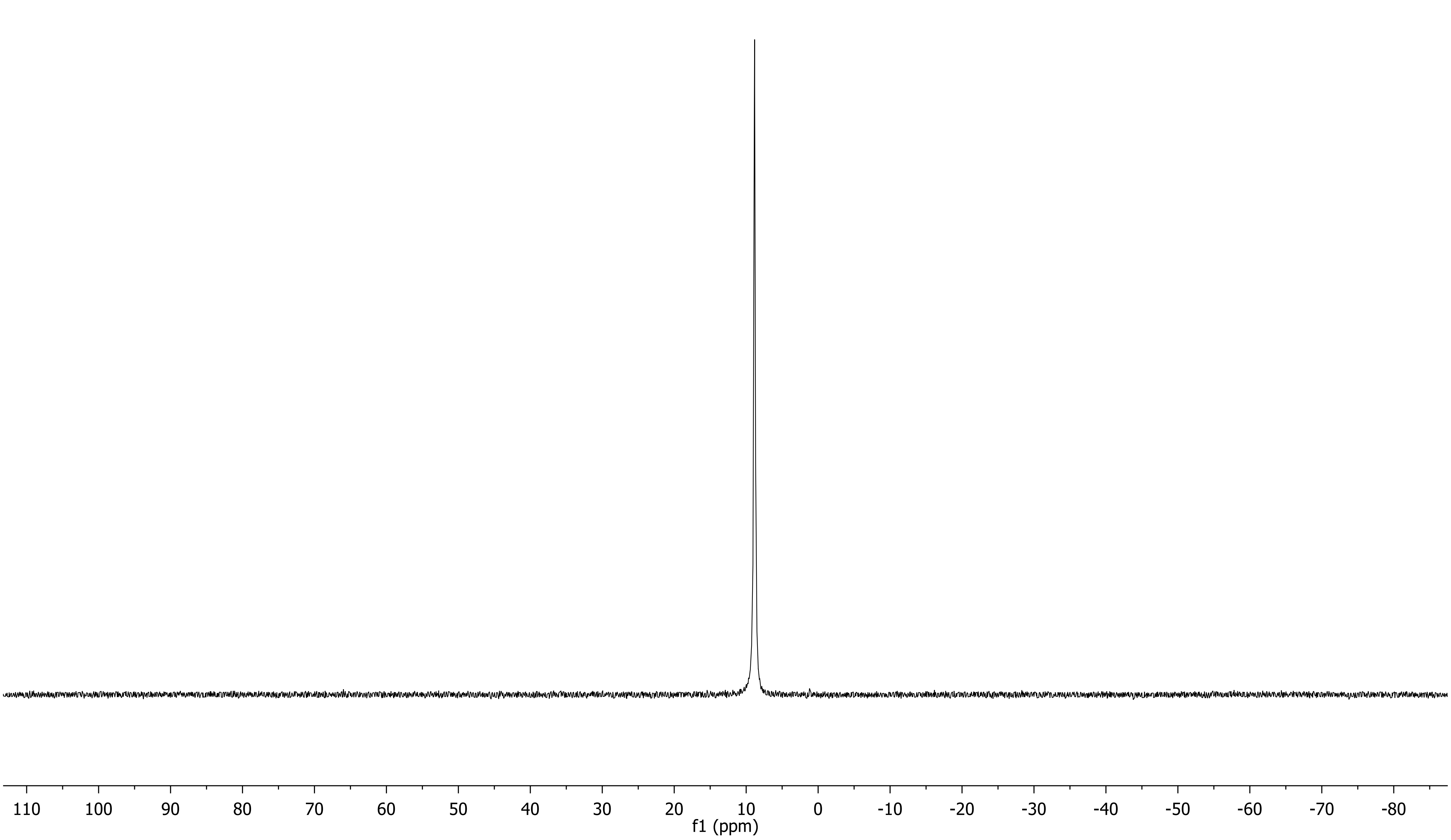

BP-11

pulse sequence - zgdc
number of scans - 1691
Finn_Dima_785
pH 1

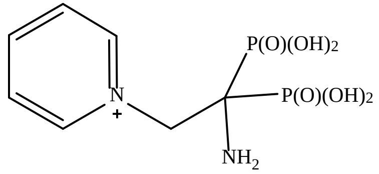

^{13}C NMR (126 MHz, Deuterium Oxide) δ 57.07 (t, J = 122.5 Hz).

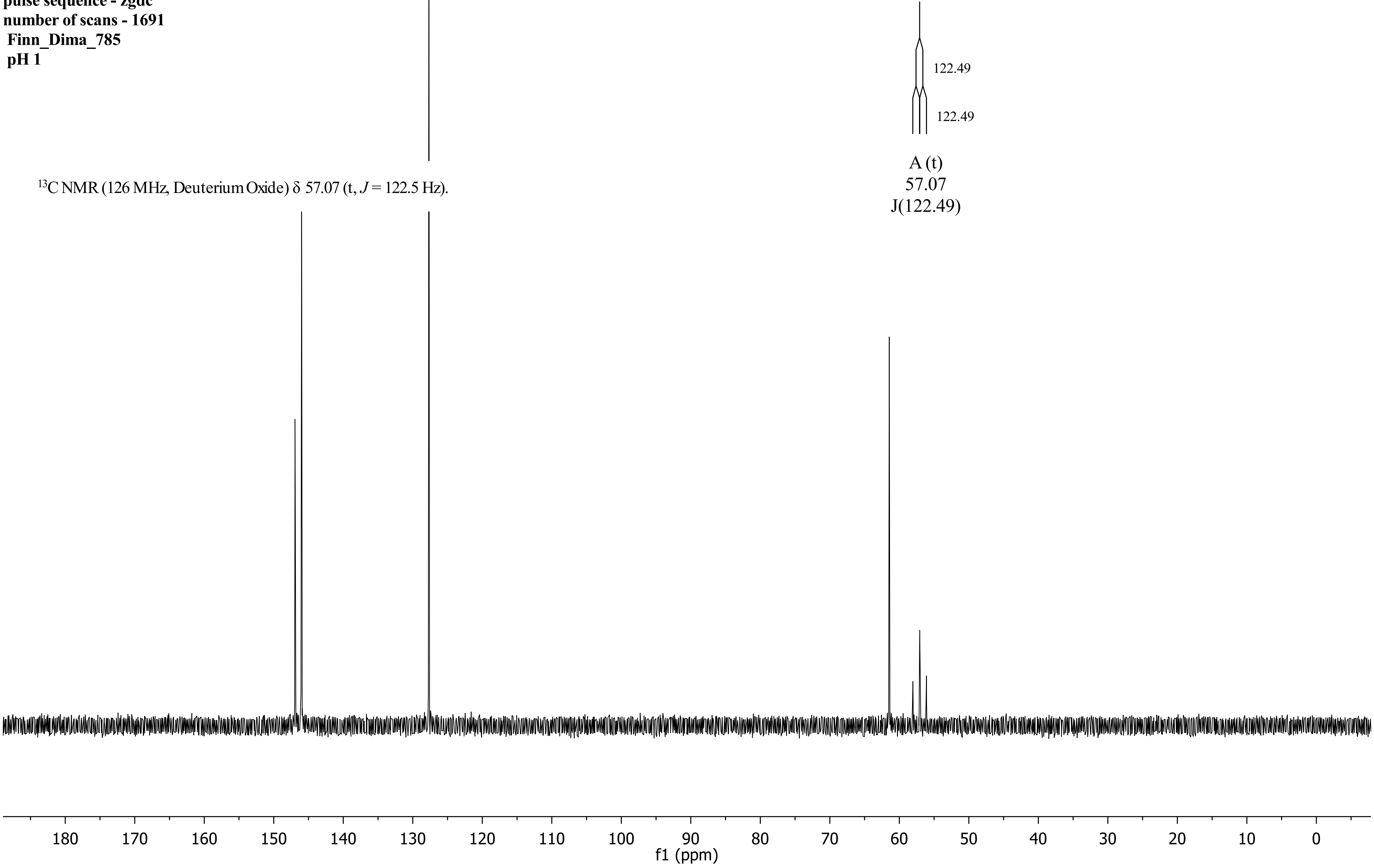

kr9784

^{1}H NMR (400 MHz, $CDCl_3$) δ 1.34 (t, *J* = 7.1 Hz, 12H).

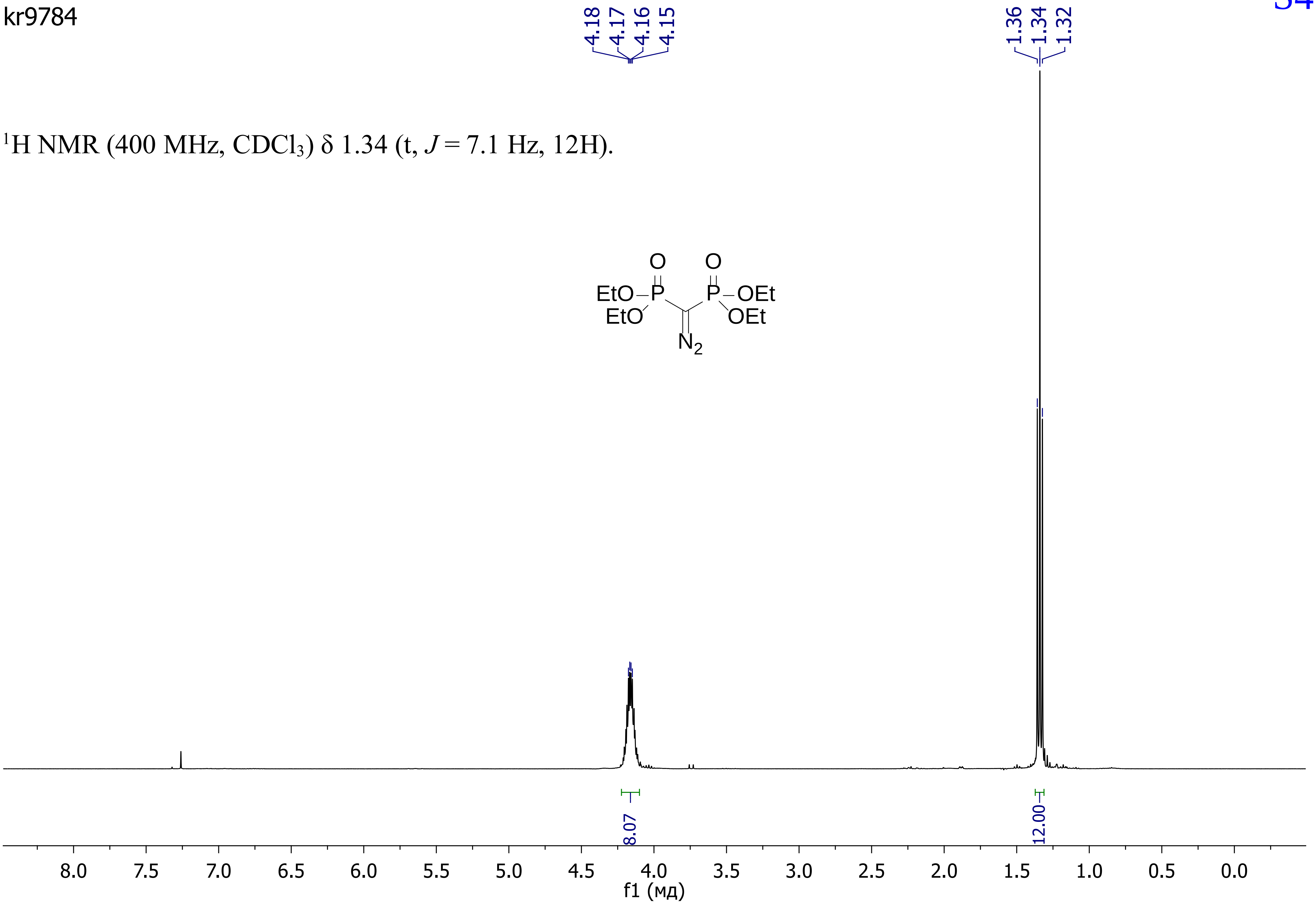

kr9784

^{1}H NMR (400 MHz, $CDCl_3$) δ 1.34 (t, *J* = 7.1 Hz, 12H).

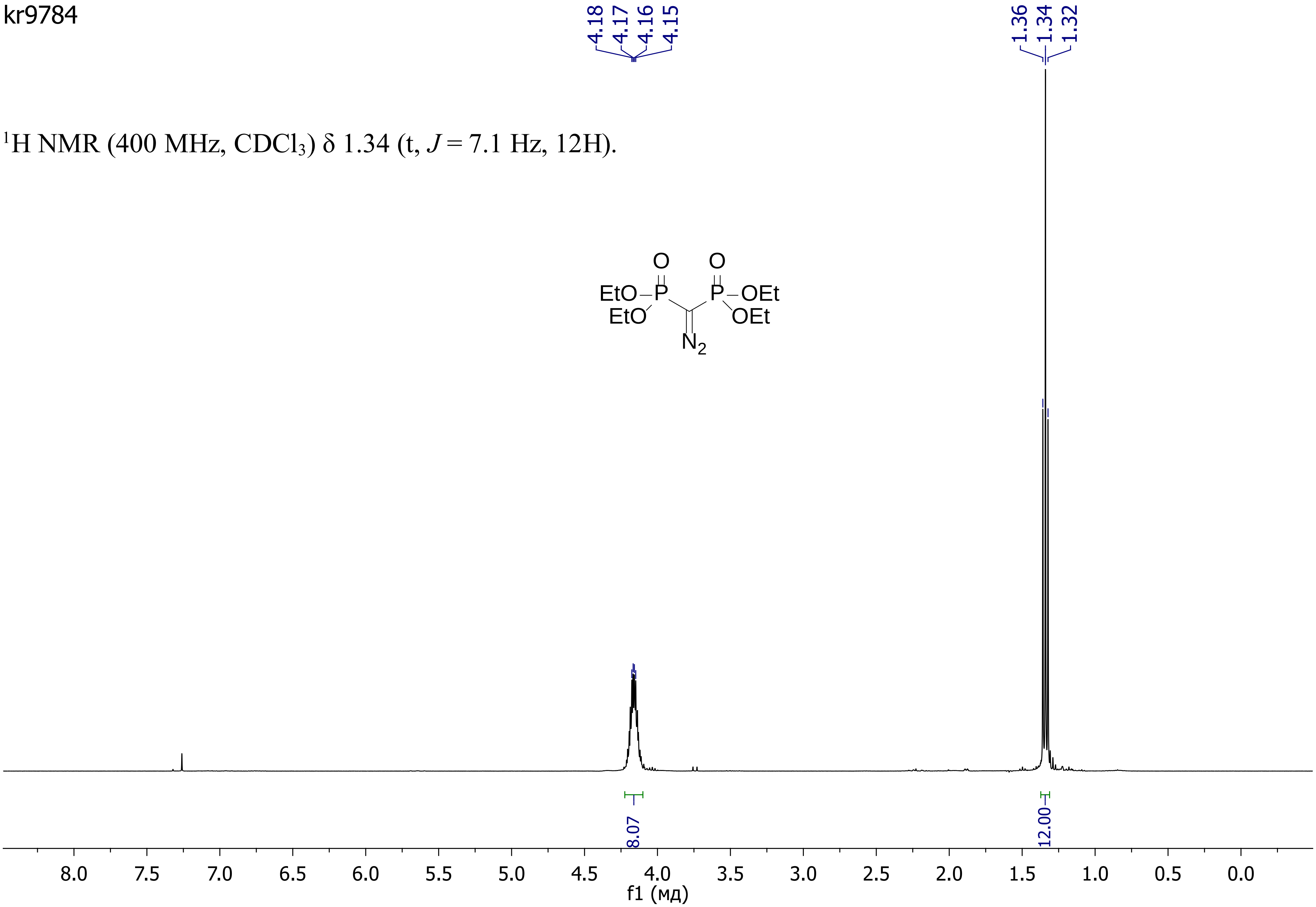

kr9784p31sup

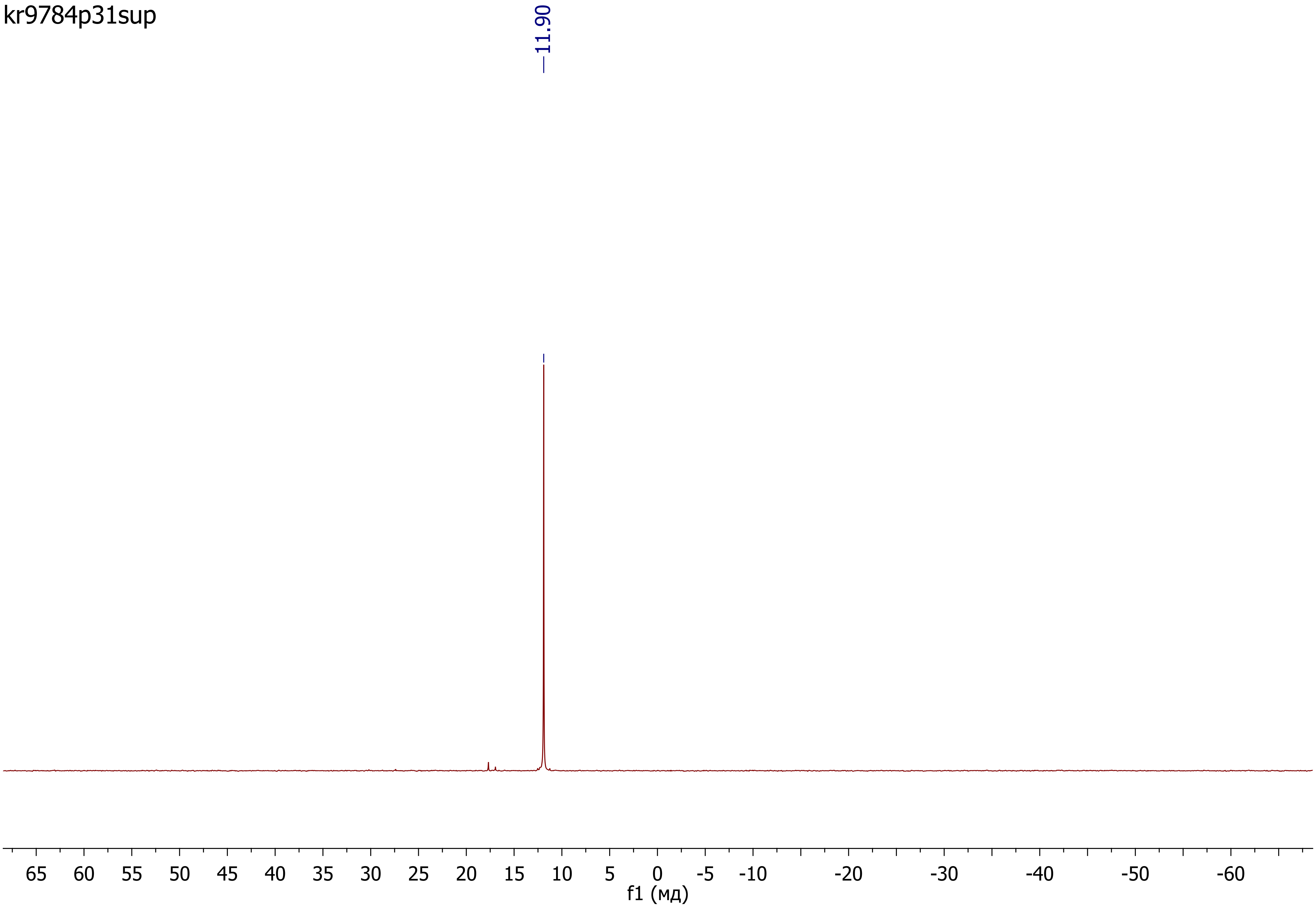

kr9784c13dec-night

^{13}C NMR (101 MHz, $CDCl_3$) δ 63.44 (t, J = 2.7 Hz), 38.84 (t, J = 204.5 Hz), 16.21 (t, J = 3.4 Hz).

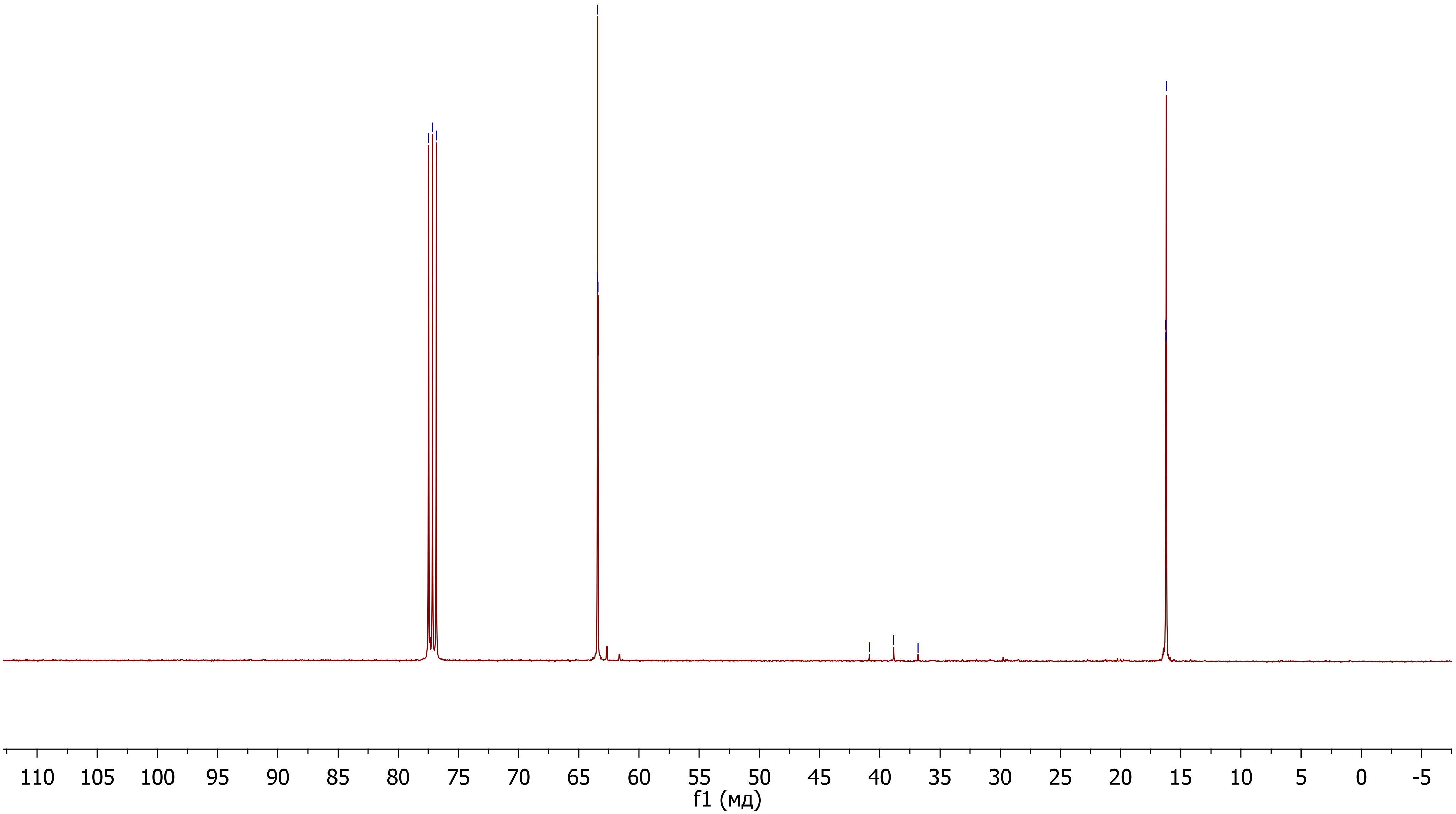

kr9784-N14-2

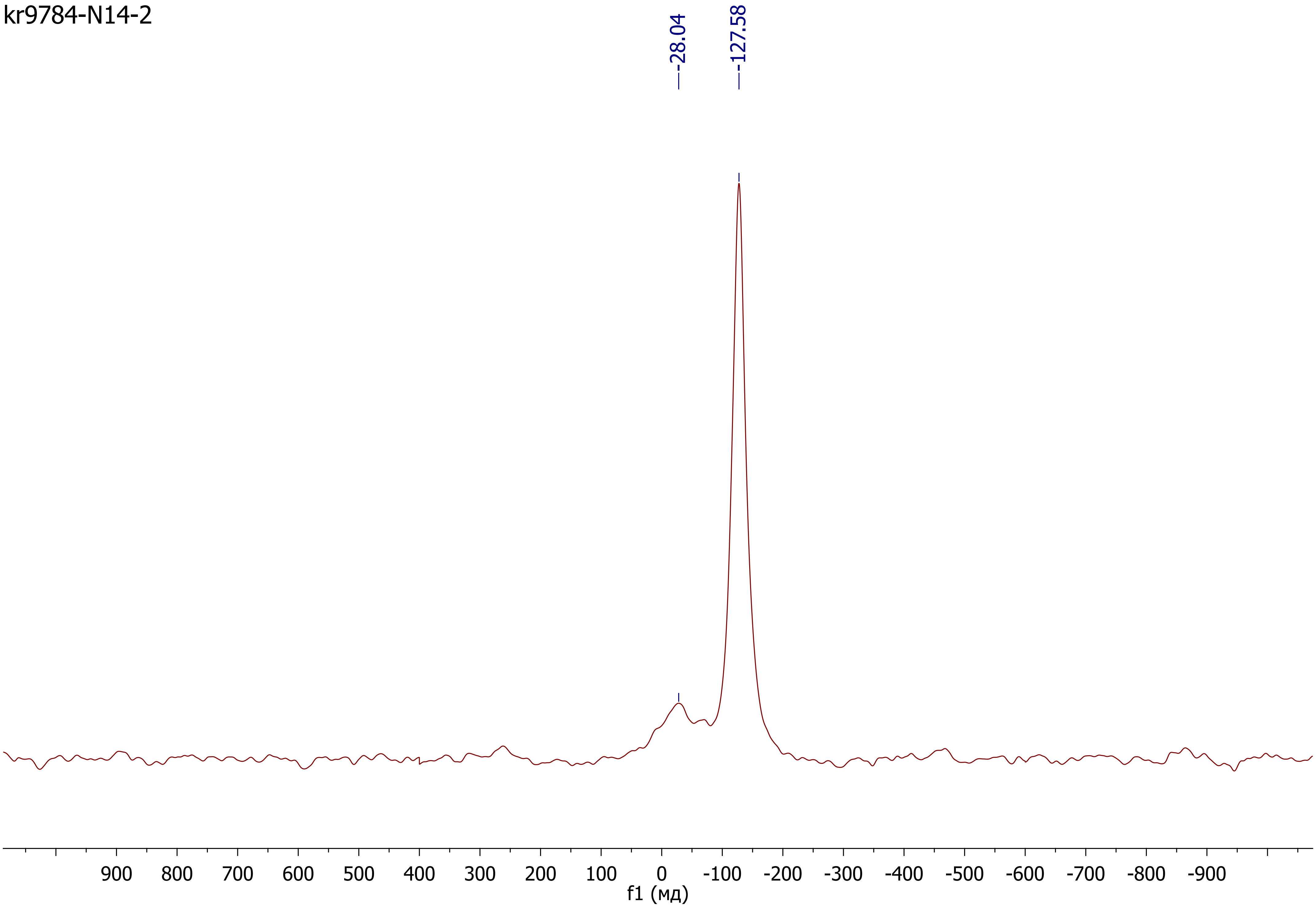

-28.04
-127.58
900
800
700
600
500
400
300
200
100
0
-100
-200
-300
-400
-500
-600
-700
-800
-900
f1 (мд)

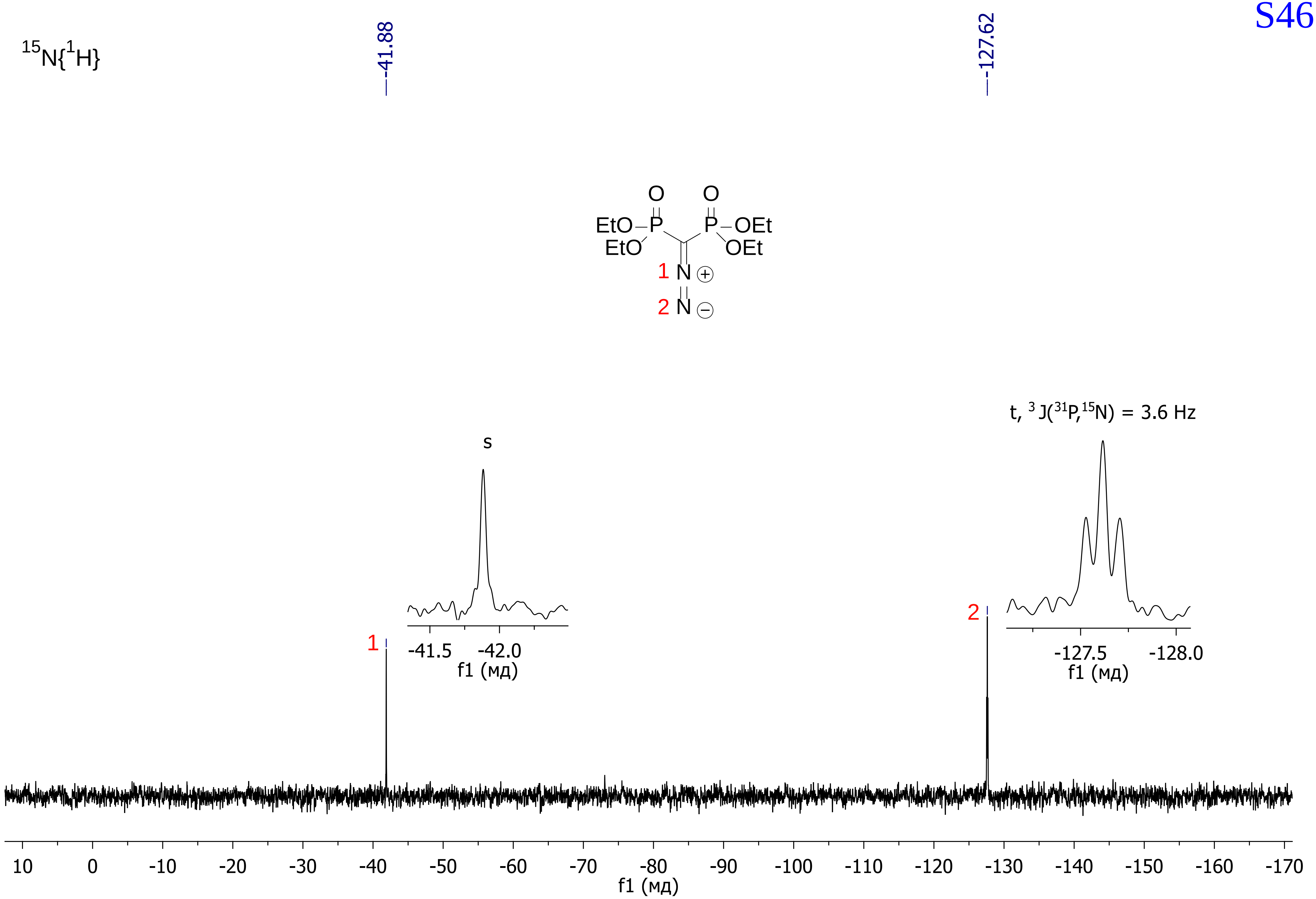

$^{15}N\{^{1}H\}$
-41.88
-127.62
1
2
s
-41.5
-42.0
f1 (мд)
t, $^{3}J(^{31}P,^{15}N)$ = 3.6 Hz
-127.5
-128.0
f1 (мд)
10
0
-10
-20
-30
-40
-50
-60
-70
-80
-90
-100
-110
-120
-130
-140
-150
-160
-170
f1 (мд)

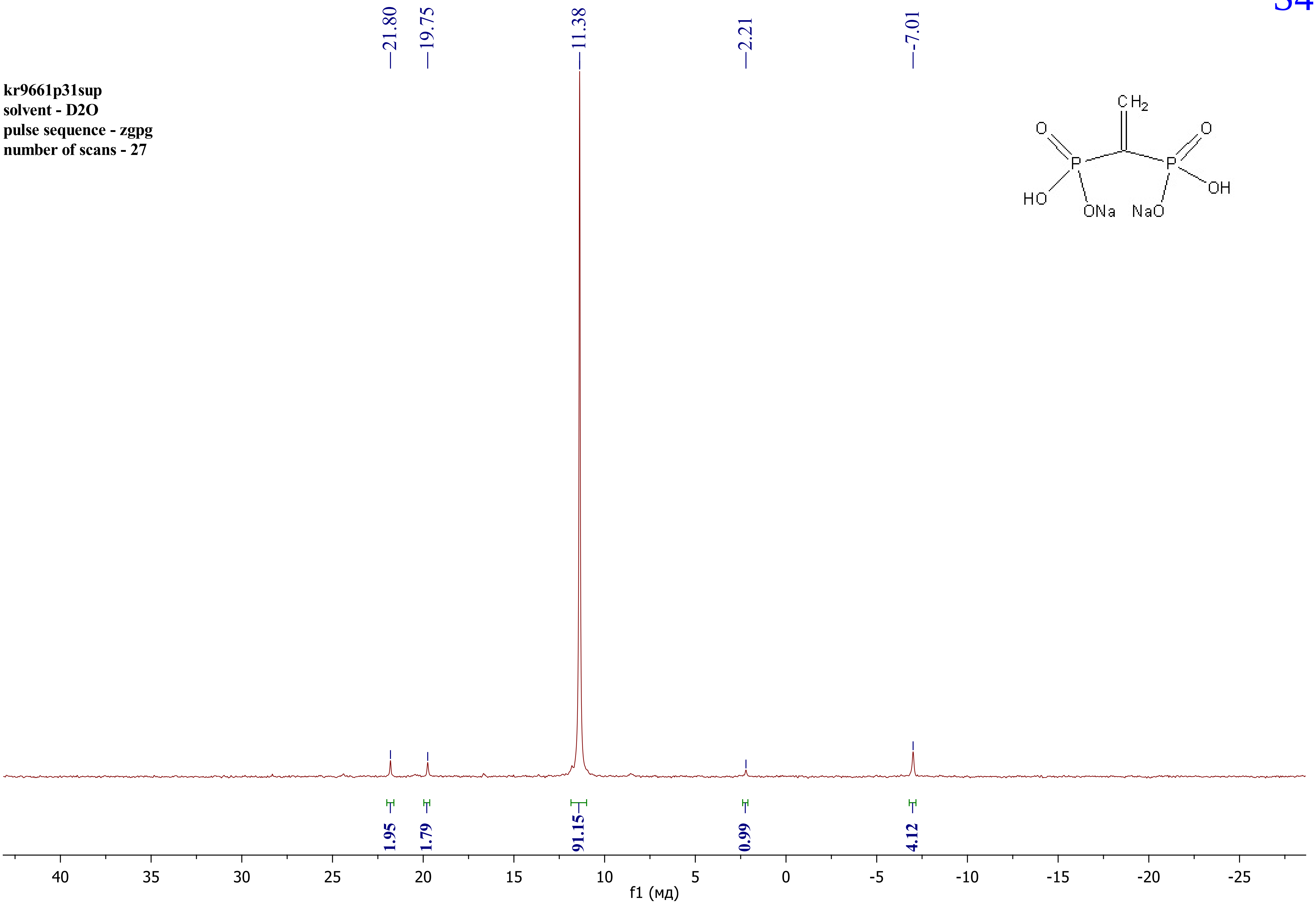
21.80
19.75
11.38
2.21
-7.01
kr9661p31sup
solvent - D2O
pulse sequence - zgpg
number of scans - 27
CH2
O
O
P
P
HO
OH
ONa
NaO
1.95
1.79
91.15
0.99
4.12
40
35
30
25
20
15
10
5
0
-5
-10
-15
-20
-25
f1 (мд)

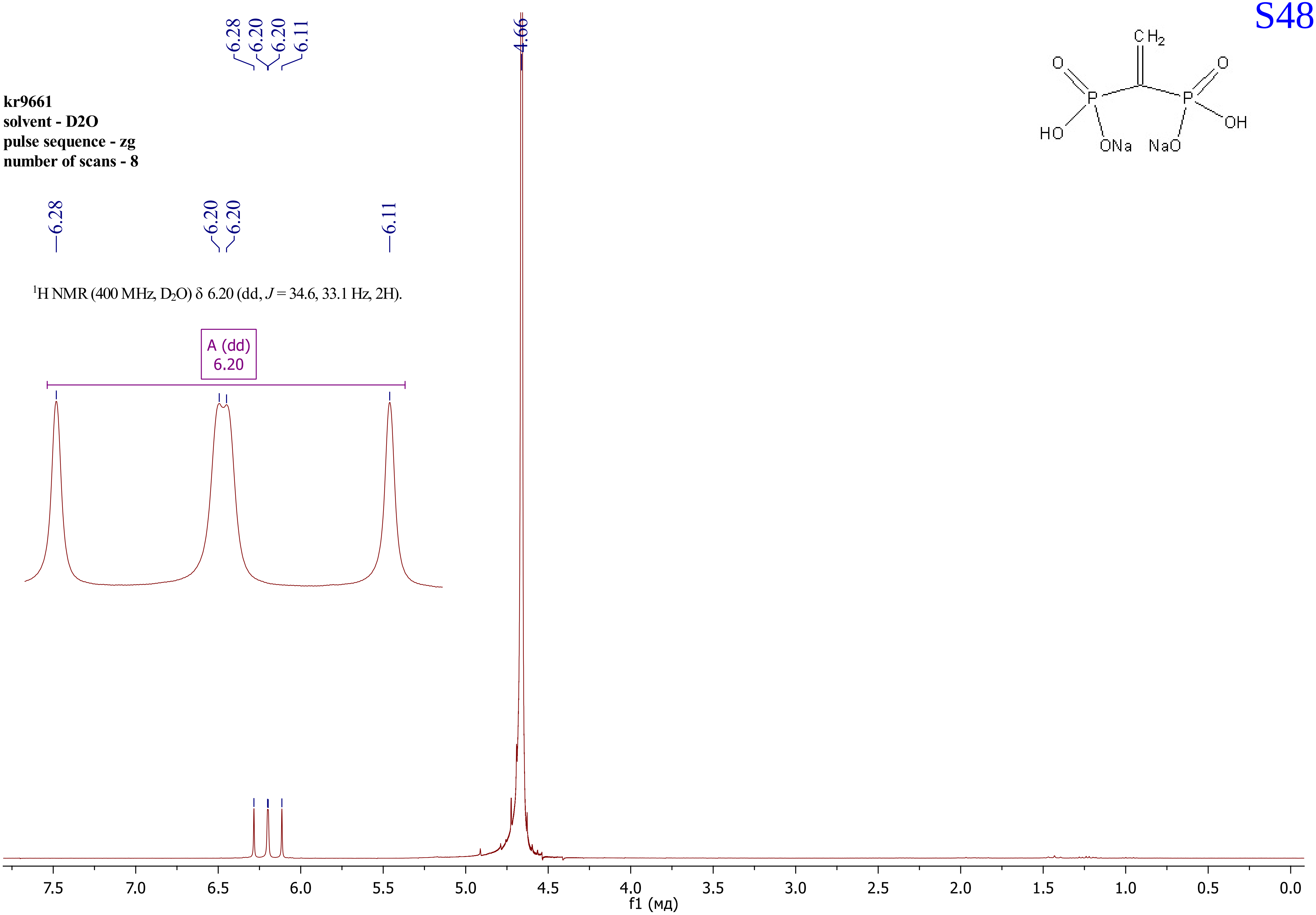
kr9661
solvent - D2O
pulse sequence - zg
number of scans - 8
6.28
6.20
6.20
6.11
4.66
¹H NMR (400 MHz, D₂O) δ 6.20 (dd, J = 34.6, 33.1 Hz, 2H).
A (dd)
6.20
CH2
O
O
P
P
HO
OH
ONa
NaO
7.5
7.0
6.5
6.0
5.5
5.0
4.5
4.0
3.5
3.0
2.5
2.0
1.5
1.0
0.5
0.0
f1 (мд)
